# Abstracts of the Total Body PET 2022 conference

**DOI:** 10.1186/s40658-023-00574-3

**Published:** 2023-09-28

## O1 Fast Monte Carlo simulation for optimizing axially extended time-of-flight PET systems

### Stephen C. Moore^*^, Margaret E. Daube-Witherspoon, Scott D. Metzler, Joel S. Karp

#### Department of Radiology, University of Pennsylvania School of Medicine, Philadelphia, Pennsylvania, USA

##### **Correspondence:** Stephen C. Moore (scmoore1@pennmedicine.upenn.edu)

*EJNMMI Physics* 2023, **10(1):**O1

**Background**

Existing Monte Carlo (MC) programs, e.g., GATE [1], accurately simulate PET scanner performance, but are slow. Because total-body (TB) PET systems with long axial fields-of-view have many lines-of-response, there is a need for fast MC programs that model relevant aspects of PET imaging for optimizing data acquisition and/or estimating scatter and spurious coincidences. We developed a fast MC (fMC) that combines algorithms described earlier for PET [2] and SPECT [3] and includes variance reduction to increase the detected photons per unit time. We evaluate here the accuracy of fMC and its utility for optimizing PET acquisitions by comparing noise-equivalent count rates (NECR) for different coincidence-sorting policies, axial coverage, and energy discriminator settings.

**Methods**

The combination of weighted forced decay, forced Compton patient scatter, forced detection, and “Delta scattering” [2] during photon propagation yields accurate and fast simulations. The PennPET Explorer [4] system was modelled, with varying axial length (# rings) for an extended (140-cm) NEMA image quality phantom containing positron + gamma emitters, i.e., ^82^Rb and ^89^Zr. ^176^Lu detector background was also simulated. Count rates and NECR values obtained using multi-window (MW) and single-window (SW) coincidence sorting [5] were compared for ^82^Rb, and fMC was used to optimize the upper-level discriminator (ULD) for ^89^Zr.

**Results**

Variance-reduction increased detected coincidences by ~ 3.8 × for ^82^Rb, providing ~ 3 times more coincidences per unit time than simulation without variance-reduction. With MW, simulated random rates agreed well with singles-based randoms, and NECR obtained with MW exceeded that from SW at all activity levels, increasing as expected with axial length of the scanner. fMC studies indicate a ULD of 585 keV is optimal for ^89^Zr imaging.

**Conclusions**

fMC simulation is efficient and accurate for optimizing current TB-PET and designing next-generation TB-PET. Future work will evaluate its performance in estimating scatter and spurious-gamma coincidences for several tracers.

The figures below illustrate just three of many possible uses of fMC for PET system evaluations and optimizations.

**Fig. 1:**
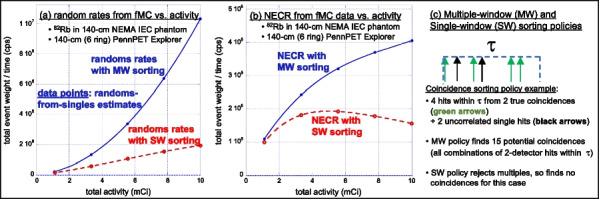
Coincidence policy evaluation shows superior performance using the multi-window (MW) coincidence policy.

**Fig. 2:**
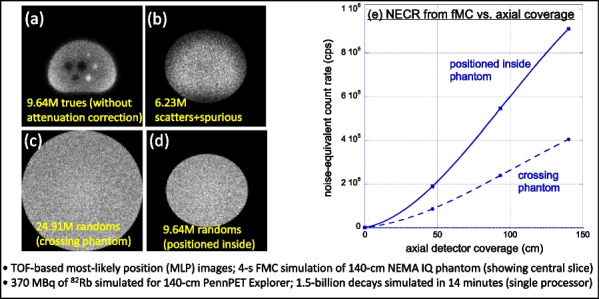
System-design study of increase in NECR with increasing axial detector coverage for ^82^Rb imaging.

**Fig. 3:**
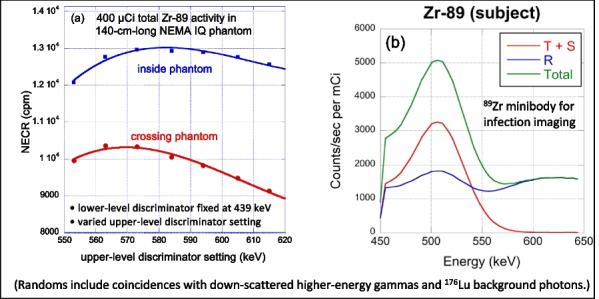
Energy window optimization of NECR for ^89^Zr imaging shows that a ULD setting near 585 keV is optimal.

**Acknowledgements**

Research reported in this publication was supported by the U.S. National Institutes of Health under grant R01-CA113941, by the National Center for Advancing Translational Sciences under Award Number UL1TR001878, and by the Institute for Translational Medicine and Therapeutics’ (ITMAT) Transdisciplinary Program in Translational Medicine and Therapeutics. The content is solely the responsibility of the authors and does not necessarily represent the official views of the NIH.

**References**

1. Jan S, Santin G, Strul D, et al. GATE: A simulation toolkit for PET and SPECT. Phys Med Biol 2004; 49: 4543–4561.

2. Holdsworth CH, Levin CS, Farquhar TH, Dahlbom M, Hoffman EJ. Investigation of accelerated Monte Carlo techniques for PET simulation and 3D PET scatter correction. IEEE Trans Nucl Sci. 2001; 48: 74–81.

3. deVries DJ, Moore SC, Zimmerman RE, Mueller SP, Friedland B, Lanza RC. Development and validation of a Monte Carlo simulation of photon transport in an Anger camera. IEEE Trans Med Imag. 1990; 9: 430–438.

4. Karp JS, Viswanath V, Geagan MJ, et al. PennPET Explorer: design and preliminary performance of a whole-body imager. J Nucl Med. 2020; 61: 136–143.

5. Oliver JF, Rafecas M. Improving the singles rate method for modeling accidental coincidences in high-resolution PET. Phys Med Biol. 2010; 55: 6951–6971.

## O2 Ultra-dense and fast ceramic scintillators for total body PET scanners

### Sun Il Kwon^1*^, Yimin Wang^2^, Urmila Shirwadkar^2^, Steven Onorato^2^, Junghune Nam^2^, Jarek Glodo^2^, Kanai S. Shah^2^, Simon R. Cherry^1^

#### ^1^Department of Biomedical Engineering, University of California Davis, Davis, CA 95616, USA; ^2^Radiation Monitoring Devices, Inc., 44 Hunt St., Watertown, MA 02472, USA

##### **Correspondence:** Sun Il Kwon (sunkwon@ucdavis.edu)

*EJNMMI Physics* 2023, **10(1):**O2

**Background**

Long axial field-of-view PET scanners provide excellent geometric coverage and offer much better detection sensitivity. However, the concern is that these are more expensive. In this study, we introduce novel lutetium-oxide (Lu_2_O_3_) based ceramic scintillators to address this concern.

**Materials and methods**

Lu_2_O_3_ has extremely high density (9.4 g/cm^3^ vs. 7.4 g/cm^3^ for LSO) and its attenuation length at 511 keV (0.93 cm vs. 1.2 cm) is superior to any other scintillator used in PET scanners to date. Due to the high stopping power, the thickness can be significantly reduced with no loss of detection efficiency compared to L(Y)SO. The reduction in thickness will also improve energy and timing performance and reduce cost and depth-of-interaction effect. Importantly, these new scintillators have a cubic crystal structure that allows fabrication using ceramic processing in place of conventional single crystal growth, with the potential for a significant reduction in manufacturing cost compared with L(Y)SO. We have fabricated and evaluated several Lu_2_O_3_-based scintillators with different combinations of ytterbium (Yb^3+^) or lanthanum (La^3+^) dopants. To estimate the detection efficiency changes with the thickness, simulation was performed.

**Results**

Our simulation showed that 15-mm Lu_2_O_3_ can achieve detection efficiency equivalent to 20-mm LSO. As for the scintillation properties, when doped only with Yb it has an exceptionally fast decay time (~ 1.7 ns), however its light yield is low (< 1500 ph/MeV). We have observed that double doping with Yb and La substantially increases the light yield (up to 20,000 ph/MeV) by introducing a slower component (Fig. 1). A good coincidence timing resolution of sub-200 ps from early 2-mm-thick samples was achieved.

**Conclusions**

The properties and evaluation of the new scintillators appear promising for PET applications, including total-body PET. By tuning co-dopant concentrations, we can optimize brightness or pulse shape as desired, depending on the ultimate application needs.

**Fig. 1:**
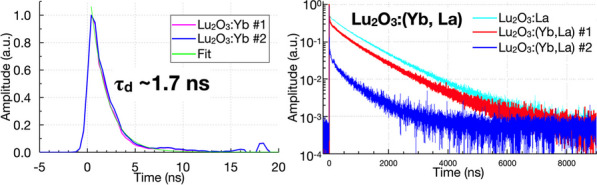
Scintillation time profiles of Lu_2_O_3_:Yb samples (left) and different Lu_2_O_3_:Yb,La samples (right).

## O3 Efficient patient throughput and detector usage in low cost efficient monolithic high resolution walk-through flat panel total body PET

### Stefaan Vandenberghe^1^*, Maya Abi Akl^1,2^, Nadia Withofs^3^, Florence-Marie Muller^1^, Jens Maebe^1^, Meysam Dadgar^4^, Xue Song^5^, Kuangyu Shi^5^, Giancarlo Sportelli^6^, Nicola Belcari^6^

#### ^1^Medical Image and Signal Processing, Ghent University, Ghent, Belgium; ^2^Science Program, Texas A&M University at Qatar; ^3^CHU of Liège, Quartier Hopital & GIGA-CRC in vivo imaging, ULiège, Belgium; ^4^Faculty of Physics, Astronomy and Applied Computer Science, Jagiellonian University, 30-348 Cracow, Poland; ^5^Insel, Bern Switzerland; ^6^Department of Physics, University of Pisa

##### **Correspondence:** Stefaan Vandenberghe (Stefaan.Vandenberghe@UGent.be)

*EJNMMI Physics* 2023, **10(1):**O3.

**Background**

Despite its very high sensitivity [1] high TB-PET throughput is limited by patient handling and shortage of personnel. Monoliths (LYSO and BGO) are valid alternative to pixelated detectors as they have a much better spatial resolution (1–1.5 mm), 6-layer DOI and CTR between 150 and 300 ps [2,3]. Therefore, they can be placed closer with a gain in both sensitivity and spatial resolution (reduced acolinearity). We design a novel monolithic low cost flat panel TB-PET system with patients in upright position (Fig. 1).

**Methods**

Patient width (PW), top head to start of legs and depth from front of the patient to bed (measured from 40 random PET-CT patients) determined flat panel size. Sensitivity and detector surface is compared to Siemens Quadra [4]. In a next phase system simulations and extensive mock-up scanner patient test will be performed to determine scatter, motion and feasible patient-throughput.

**Results**

The average/max width/height/depth of the 40 patients was 52/65, 85/95 and 32/38 cm. This justifies a design of 70 cm wide, 105 cm high and 50 cm gap. The number of detectors (same FOV) is 1.9 × less than in a Siemens Quadra for similar sensitivity. Spatial resolution will be less than 2 mm over the whole FOV (reduced acollinearity from 80 to 50 cm). The estimated component cost for 12 mm thick monolithic BGO/6 mm SiPM/readout is only 1.3 MEuro. DL will be applied on images from 50% sparse BGO detectors to reduce system cost to that of a standard PET scanner. Scatter and attenuation correction can be applied (without CT) to non-attenuation corrected reconstructed using DL [5]. This enables fast, low dose imaging and frequent screening. Personnel costs can be reduced by letting patient start the acquisition via simple touch buttons. The footprint of the scanner is about 1m^2^.

**Fig. 1:**
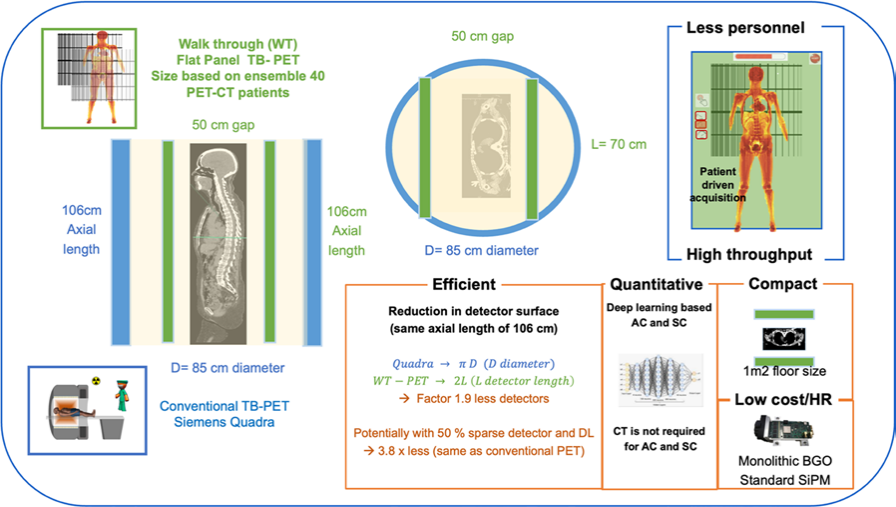
Design of the flat panel walk-through TB-PET.

**References**

1. Vandenberghe S, Moskal P, Karp JS. State of the art in total body PET. EJNMMI Phys. 2020; 7(1):35.

2. Mariele Stockhoff, Milan Decuyper, Roel Van Holen, Stefaan Vandenberghe. High-resolution monolithic LYSO detector with 6-layer depth-of-interaction for clinical PET. Phys Med Biol. 2021; 66(15):10.1088/1361-6560.

3. P. Carra, M.G. Bisogni, E. Ciarrocchi, M. Morrocchi, V. Rosso, G. Sportelli, N. Belcari. Performance of monolithic BGO-based detector implementing a Neural-Network event decoding algorithm for TB-PET applications, presentation at Elba PSMR-TB-PET conference 2022¶

4. Prenosil GA, Sari H, Fürstner M, Afshar-Oromieh A, Shi K, Rominger A, Hentschel M. Performance Characteristics of the Biograph Vision Quadra PET/CT system with long axial field of view using the NEMA NU 2–2018 Standard. J Nucl Med. 2022; 63(3):476–484.

5. Song Xue, Karl Peter Bohn,Rui Guo,Hasan Sari, Marco Viscione,Axel Rominger, Biao Li, Kuangyu Shi, Development of a deep learning method for CT-free correction for an ultra-long axial field of view PET scanner, abstract presented at the PSMR-TBPET conference, Elba 2022.

## O4 CD8-targeted total-body PET imaging of T cells in patients recovering from COVID-19

### Negar Omidvari^1*^, Terry Jones^2^, Pat M Price^3^, Fatma Sen^2^, Barbara Shacklett^4^, Stuart Cohen^5^, Ramsey D Badawi^1,2^, Ian Wilson^6^, and Simon R Cherry^1,2^

#### ^1^Department of Biomedical Engineering, University of California Davis, Davis, CA, USA; ^2^Department of Radiology, University of California Davis Medical Centre, Sacramento, CA, USA; ^3^Department of Surgery and Cancer, Imperial College, London, United Kingdom; ^4^Department of Medical Microbiology and Immunology, School of Medicine, University of California, Davis, CA, USA; ^5^Division of Infectious Diseases, Department of Internal Medicine, University of California Davis Medical Center, Sacramento, CA, USA; ^6^ImaginAb, Inc., Inglewood, CA, USA

##### **Correspondence:** Negar Omidvari (nomidvari@ucdavis.edu)

*EJNMMI Physics* 2023, **10(1):**O4

**Background**

CD8+ T cells are key players in immune response. Following viral infection or vaccination, a small portion of antigen-specific T cells differentiate into memory cells, forming a long-term protective memory against reinfection. However, in vivo information about COVID-19-specific T cell immunity is limited, since 95% of T cells are not in the circulation and tissue sampling has been minimal^[1,2]^. This pilot study aims to provide an in vivo measure of tissue distribution of CD8+ T cells after COVID-19 infection, using total-body imaging of a labeled minibody with high affinity to human CD8.

**Materials and methods**

5 COVID-19-recovered patients and 3 healthy controls were studied (Table 1). Subjects received ~ 0.5 mCi of ^89^Zr-Df-Crefmirlimab-Berdoxam^[3,4]^ and had 60-min total-body PET/CT scans at 6-h and 48-h post-injection. Control subjects and 3 COVID-19 patients had an additional 90-min dynamic scan. Scans of 3 COVID-19 patients were repeated after 4 months. Volume-of-interest were drawn on spleen, liver, lungs, bone marrow, lymph nodes, tonsils, and blood-pool. Two-tissue compartmental modelling was performed on the dynamic data to derive $K_{i} = \frac{{K_{1} k_{3} }}{{k_{2} + k_{3} }}$ and $V_{T} = \frac{{K_{1} }}{{k_{2} }}\left( {1 + \frac{{k_{3} }}{{k_{4} }}} \right)$.

**Results**

In all subjects, activity decrease in bone marrow and spleen between 6 and 48 h was observed with parallel activity increase in lymph nodes and tonsils, suggesting cell-trafficking (Fig. 1). Tissue-to-plasma ratio (0‒7 h), $K_{i}$, and $V_{T}$ were higher in bone marrow of the COVID-19 patients than controls and were the highest in one COVID-19 patient, infected twice with the virus (Fig. 2).

**Conclusions**

Total-body imaging of CD8 + T cells with sub-millicurie levels of ^89^Zr-labeled tracer resulted in the ability to quantify rates of uptake and concentrations of the tracer in lymphoid tissues throughout the body, along with T cell migration over a 48-h period. Current data suggest that the bone marrow T-cell pool in COVID-19-recovered patients is larger or has increased CD8 expression compared to controls.

**Table 1:** Demographics of the 8 subjects scanned on the uEXPLORER total-body PET scanner.

Subject number	Study group	Sex	Age	BMI	Scans	Vaccination and diagnosis times
1	COVID-19	F	51	38	6 h, 48 h (4-month follow up completed)	Infected with COVID-19 before vaccination. First PET scan was 11 days after the first vaccination dose and 7 weeks after the first positive PCR test
2	COVID-19	F	29	29	Dynamic (90 min), 6 h, 48 h (4-month follow up completed)	Infected with COVID-19 13 days after the first vaccination dose. Tested positive again 3 months after first diagnosis. First PET scan was 4 months after the second vaccine dose and 7 weeks after the second positive PCR test
3	COVID-19	F	46	43	6 h, 48 h (4-month follow up completed)	Infected with COVID-19 3.5 months after the second vaccination dose. First PET scan was 4.5 months after the second vaccination dose and 8 weeks after the first positive PCR test
4	Control	M	25	21	Dynamic (90 min), 6 h, 48 h	First PET scan was 6 months after the second vaccination dose
5	Control	M	49	25	Dynamic (65 min), 6 h, 48 h	First PET scan was 6 months after the second vaccination dose
6	Control	F	59	31	Dynamic (90 min), 6 h, 48 h	Not vaccinated
7	COVID-19	F	27	35	Dynamic (90 min), 6 h, 48 h	Infected with COVID-19 3 months after the booster vaccination dose. First PET scan was 4.5 months after the second vaccination dose and 6 weeks after the first positive PCR test
8	COVID-19	F	34	20	Dynamic (90 min), 6 h, 48 h	Infected with COVID-19 8 months after the second vaccination dose. First PET scan was 9.5 months after the second vaccination dose and 5.5 weeks after the first positive PCR test

**Fig. 1:**
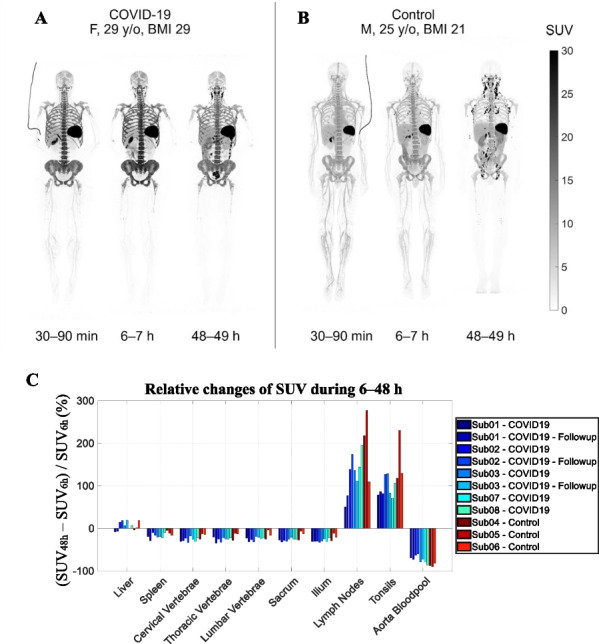
**A**, **B** Example SUV maximum intensity projections of **A** a recovered COVID-19 patient, compared to (B) a healthy subject scanned on the uEXPLORER at three timepoints. **C** Percentage changes of uptake in organs of interest during 6‒48 h timepoints compared in all subjects.

**Fig. 2:**
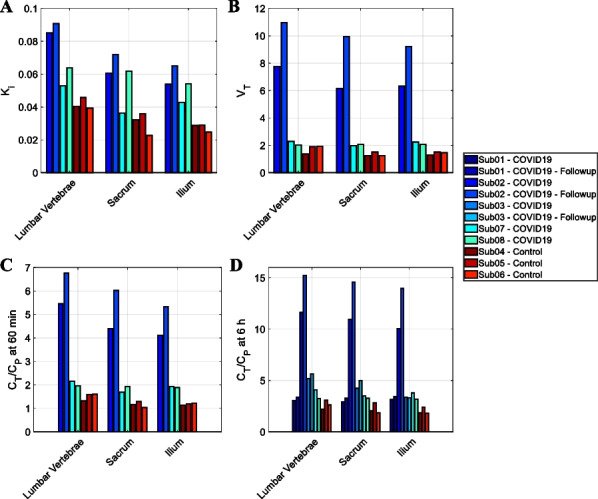
**A** K_i_ and **B** V_T_ of two-tissue-compartmental modelling and tissue-to-plasma ratios at **C** 60–65 min and **D** 6–7 h timepoints of pelvic bone marrow show higher values in COVID-19-recovered patients compared to the controls and are the highest in Subject 2, twice infected with the virus. The 4-month follow-up scans in COVID-19 patients show increased K_i_, V_T_ and tissue-to-plasma ratios compared to the initial scans. The measurements in vaccinated and unvaccinated controls were similar.

**References**

1. Altmann DM, Boyton RJ. SARS-CoV-2T cell immunity: Specificity, function, durability, and role in protection. Sci Immunol 2020; 5(49).

2. Farber DL, Tissues, not blood, are where immune cells act, Nature 2021; Vol 593: 507–509.

3. Pandit-Taskar N, Postow MA, Hellmann MD, et al. First-in-Humans Imaging with (89)Zr-Df-IAB22M2C Anti-CD8 Minibody in Patients with Solid Malignancies: Preliminary Pharmacokinetics, Biodistribution, and Lesion Targeting. J Nucl Med 2020; 61(4): 512–9.

4. Farwell M D, Gamache R F, Babazada H, et al. Journal of Nuclear Medicine, CD8-targeted PET Imaging of Tumor Infiltrating T cells in Patients with Cancer: A Phase I First-in-Human Study of 89Zr-Df-IAB22M2C, a Radiolabeled anti-CD8 Minibody published on August 19, 2021 as https://doi.org/10.2967/jnumed.121.262485

## O5 Total-body dynamic PET/CT imaging of ^11^C-methionine in multiple myeloma

### Jiajin Li^1^, Xiaofeng Yu^1^, Yun Zhou^2^, Yue Gu^2^, Jianjun Liu^1^, Yumei Chen^1,*^

#### ^1^Department of Nuclear Medicine, Ren Ji Hospital, Shanghai Jiao Tong University School of Medicine, Shanghai 200,127, China; ^2^Central Research Institute, United Imaging Healthcare Group Co., Ltd, Shanghai 200032, China

##### **Correspondence:** Yumei Chen (cymrenji@126.com)

*EJNMMI Physics* 2023, **10(1):**O5.

**Background**

uEXPLORER as the first 2-m long total-body PET scanner, has brought tracer kinetics analysis into a new era. [^11^C] methionine (^11^C-MET) has advantages over ^18^F-FDG for PET imaging in multiple myeloma (MM). The study aims to establish a kinetic model suitable for total-body dynamic ^11^C-MET PET/CT and to evaluate its clinical application value in multiple myeloma.

**Methods and material**

Dynamic total-body ^11^C-MET PET/CT was conducted with uEXPLORER in 9 subjects (7 MM patients and 2 controls). The time-activity curves (TACs) of organs and bone marrows were extracted. Model fitting of TACs was operated using PMOD Kinetic Modeling through a reversible two-tissue compartment model (rev2TCM), an irreversible two-tissue compartment model, a patlak plot, and a logan plot respectively. The four models were optimized by (1) visual inspection and (2) goodness-of-fit displayed by the Akaike information criterion (AIC) and Schwartz information criterion (SC). R software was used to analyze the correlation between dynamic parameters and clinical indicators.

**Results**

The rev2TCM has a best fitting with TACs among the four models (AIC = − 12.238, SC = 0.371, MSC = 5.328 for bone marrow). MM patients had higher ^11^C-MET uptakes in bone marrow compared with controls (SUVmax: 14.5 ± 6.6 vs 6.2 ± 0.8 g/mL, *p* = 0.014). The dynamic parameters (Flux: r = 0.848, *p* = 0.016; Vs: r = 0.788, *p* = 0.035 and Vt: r = 0.793, *p* = 0.034) rather than SUVmax (r = 0.703, *p* = 0.078), were found to be correlated with M protein levels in MM patients.

**Conclusion**

This study demonstrated the capability of dynamic total-body ^11^C-MET PET/CT in visualizing MM. The dynamic parameters achieved by the rev2TCM have an advantage in evaluating the progression of MM patients.

**Acknowledgements**

The study was sponsored by the National Natural Science Foundation of China (Grant Nos.82102089). We acknowledge the contributions of all team members from United Imaging Healthcare and Ren Ji Hospital.

## O6 Feasibility of only delayed imaging for ^68^ Ga-PSMA PET/CT: initial clinical experience of total-body PET/CT in patients with prostate cancer

### Xiaofeng Yu^1^, Lian Xu^1^, Lianghua Li^1^, Cheng Wang^1^, Ying Wang^2^, Gang Huang^3^, Jianjun Liu^1^, Yumei Chen^1,*^

#### ^1^Department of Nuclear Medicine, Renji Hospital, School of Medicine, Shanghai Jiaotong University, Shanghai, People’s Republic of China; ^2^United Imaging Healthcare, Shanghai, People’s Republic of China; ^3^Shanghai Key Laboratory of Molecular Imaging, Shanghai University of Medicine and Health Sciences, Shanghai, People’s Republic of China

##### **Correspondence:** Yumei Chen (cymrenji@126.com)

*EJNMMI Physics* 2023, **10(1):**O6

**Background**

Though dual-time point ^68^Ga-PSMA PET/CT increases the detection rate in PCa patients, it remains difficult to perform due to low count statistics in delayed imaging. The aim of this study was to prove the feasibility of only one-time delayed acquisition of ^68^Ga-PSMA on total-body PET/CT and to compare the image quality and lesion detection rate with standard acquisition.

**Materials and methods**

A retrospective study between December 2020 and July 2021 was performed in 56 PCa patients who underwent ^68^Ga-PSMA-11 total-body PET/CT (uEXPLORER), image quality was compared using 5-point Likert scale. All received a standard acquisition at 1 h p.i. and a late acquisition at 3 h p.i., lesions uptakes and TBR were compared to assess lesion detection rate. And all lesions were further analyzed based on uptake values, sizes, types and sites.

**Results**

On 5-point Likert scale, the delayed image quality was assessed as modestly lower compared to the standard image (4.1 ± 0.6 at 3 h p.i. vs 4.9 ± 0.4 at 1 h p.i., *p* < 0.001). The delayed images showed increased PSMA-avid lesion uptake values (SUV_max_) (11.0 [2.3–193.6] vs 7.0 [2.0–124.3], *p* < 0.001), and elevated TBR (3.3 [0.5–62.2] vs 1.7 [0.3–30.7], *p* < 0.001). Delayed images provided additional lesion detection in 14 of 56 patients, impacting management plans in 8 of the 14 patients. Late acquisitions detected 9.7% additional PSMA-avid lesions (22/226), 13 lesions were small LN (D < 10 mm) with higher SUV_max_ and TBR.

**Conclusion**

Compared with standard imaging, delayed imaging with ^68^Ga-PSMA-11 total-body PET/CT offered higher lesion uptake and lesion contrast. The additional lesions provided valuable information in PCa patients. The proposed protocol with only one-time acquisition at 3 h p.i. is feasible on total-body PET/CT with an AFOV of 194 cm.

## O7 ENHANCE-PET: a software framework to explore inter-organ metabolic networks in total-body [18F]FDG-PET

### Daria Ferrara^1,*^, Ramsey D. Badawi^2^, Benjamin A. Spencer^3^, Simon R. Cherry^3^, Marcus Hacker^4^, Thomas Beyer^1^, and Lalith Kumar Shiyam Sundar^1^

#### ^1^Quantitative Imaging and Medical Physics (QIMP) Team, Center for Medical Physics and Biomedical Engineering, Medical University of Vienna, Austria; ^2^Department of Radiology, UC Davis, Sacramento, CA, USA; ^3^Department of Biomedical Engineering California Davis, CA, USA; ^4^Department of Biomedical Imaging and Image-Guided Therapy, Division of Nuclear Medicine, Medical University of Vienna, Austria

##### **Correspondence:** Daria Ferrara (daria.ferrara@meduniwien.ac.at)

*EJNMMI Physics* 2023, **10(1):**O7

**Aim/background**

With the recent introduction of PET systems with an extended axial field-of-view, simultaneous, quantitative imaging of multiple distant organs has become within reach. Here, we propose an automated framework to explore homeostasis and disease-induced inter-organ metabolic perturbations using [18F]FDG total-body (TB) PET images.

**Materials and methods**

The uEXPLORER PET/CT system was used to perform dynamic TB-PET imaging on 15 healthy volunteers (26–78 years, 53–112 kg, 6 M/9F), injected with (372 ± 17) MBq of [18F]FDG. A low-dose CT was acquired for attenuation and scatter correction. A software framework (ENHANCE) was built to explore functional connectivity between different organs in TB [18F]FDG PET data. This framework is made of three components: (1) a segmentation tool based on 3D-UNet to segment 13 different organs (brain, thyroid, aorta, lung, inferior vena cava, heart, liver, pancreas, spleen, kidneys, adrenal glands, bones, bladder) from low-dose CT; (2) an algorithm to perform motion correction using diffeomorphic registration; and (3) a normative functional connectivity module to analyse time-activity-curves in multiple organs and identify group connectivity based on temporal correlations. A Fisher's Z-transformation was used on data derived with the framework to create a group-averaged normative correlation network for both male/female healthy volunteers.

**Results**

Each node of a network plot (Fig. 1) represents an organ, and its length depicts its degree of connectivity to other organs. The thickness of the curves indicates the strength of the correlation. Nodal sizes of brain, heart, liver, bone, and lung were different between the male/female cohorts, thus, indicating different inter-organ connectivities between the two populations.

**Conclusions**

Our framework, ENHANCE, is applicable to PET data from any TB-PET system, and supports the investigation of inter-organ connectivities in an approach to perform whole-person research.

**Fig. 1:**
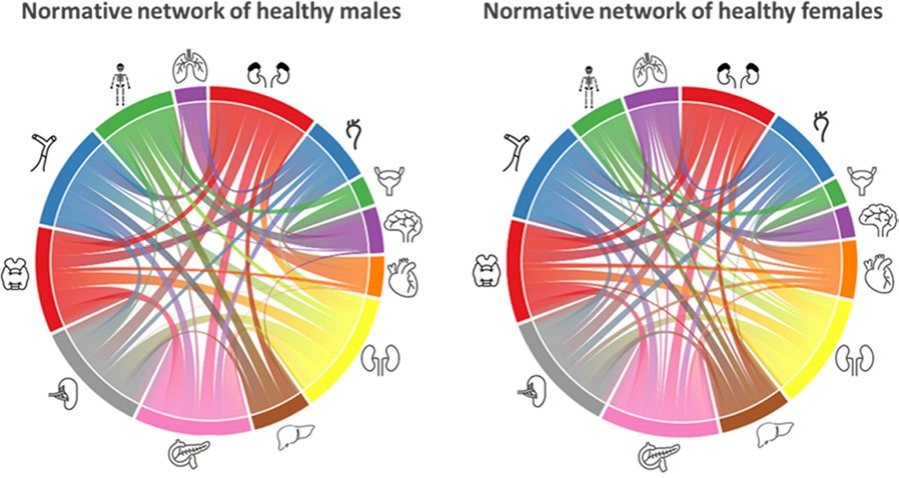
Normative networks of male/female healthy cohorts using 13 reference organs.

## O8 Total-body PET parametric imaging using Deep Patlak: a deep-learning kinetic modeling method inspired by the Patlak plot

### Yiran Wang^1,2,*^, Siqi Li^1^, Benjamin Spencer^2, 1^, Rashmi Verma^3^, Mamta Parikh^3^, Lorenzo Nardo^1^, Ramsey D Badawi^1,2^, Simon R Cherry^2, 1^, Guobao Wang^1^

#### ^1^Department of Radiology, University of California Davis Medical Center, Sacramento, CA, 95817, USA; ^2^Department of Biomedical Engineering, University of California - Davis, Davis, CA, 95616, USA; ^3^Comprehensive Cancer Center, University of California—Davis, Sacramento, CA, 95,817, USA

##### **Correspondence:** Yiran Wang (yrdwang@ucdavis.edu)

*EJNMMI Physics* 2023, **10(1):**O8

**Background**

Compartmental modeling (CM) [1] is a standard method for estimating the tracer influx rate $K_{{\text{i}}}$ but is time-consuming for parametric imaging in total-body dynamic PET. The conventional Patlak (CP) plot [2] is fast but less accurate due to simplification. In this paper, we generalize CP with deep neural networks to form a Deep Patlak (DP) method for both high accuracy and efficiency for total-body PET parametric imaging.

**Materials and methods**

We describe CP using an equivalent input–output network (Fig. 1A). The nonlinear transformations $\Psi_{{\text{X}}}$ and $\Psi_{{\text{Y}}}$ are model-driven with closed-form expressions. In the proposed DP, the two transformations are data-driven as implemented by two neural networks (Fig. 1B). The DP model can be directly trained on a single subject by using a small fraction (e.g., 10%) of body voxels labeled by the $K_{{\text{i}}}$ s estimated by CM (two-tissue irreversible model with time delay correction and model selection) [3, 4]. The trained DP model is then used to predict $K_{{\text{i}}}$ for the rest voxels. We tested the DP method for total-body $K_{{\text{i}}}$ parametric imaging in sixteen subjects (nine healthy and seven cancer patients), each with an ^18^F-FDG dynamic scan on the uEXPLORER PET/CT system. Using the CM $K_{{\text{i}}}$ as the reference, root-mean-squared error (RMSE) was compared between DP and CP for global image quality and ROI quantification. Time costs were also compared.

**Results**

Compared with CM, CP underestimated lesion $K_{{\text{i}}}$ (Fig. 2A) while DP overcame this problem. DP achieved 74% and ~ 80% lower RMSE than CP for total-body $K_{{\text{i}}}$ parametric imaging (Fig. 2B) and ROI estimates (Fig. 3) in the sixteen subjects, respectively. The CM took ~ 3.3 h per subject, while CP and DP took < 0.5 h.

**Conclusions**

The proposed Deep Patlak method achieved higher accuracy than conventional Patlak and higher efficiency than traditional compartmental modeling for total-body $K_{{\text{i}}}$ parametric imaging.

**Fig. 1:**
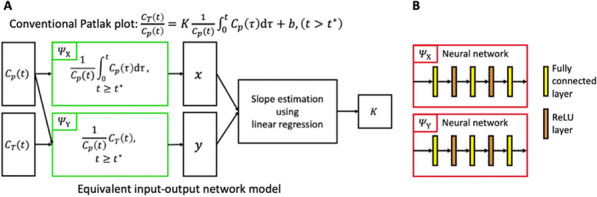
**A** Equivalent input–output network of the conventional Patlak plot with the nonlinear transformations $\Psi_{{\text{X}}}$ and $\Psi_{{\text{Y}}}$ in closed-form expressions. **B** $\Psi_{{\text{X}}}$ and $\Psi_{{\text{Y}}}$ are replaced with neural networks in the proposed Deep Patlak model.

**Fig. 2:**
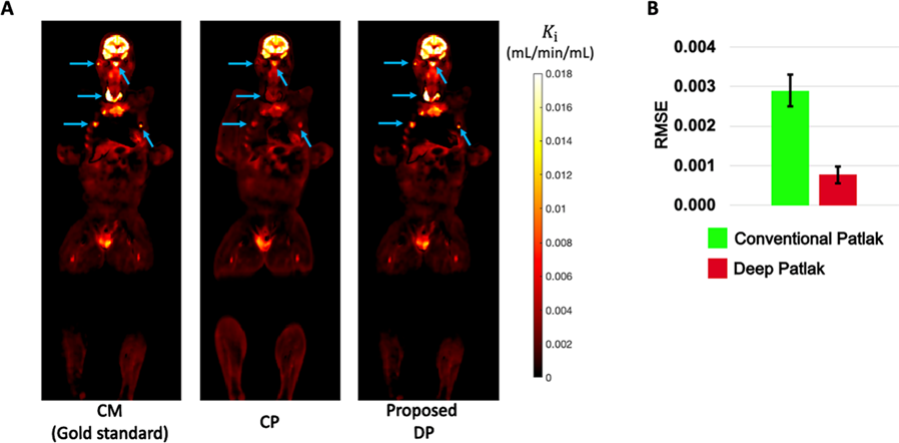
**A** Total-body K_i parametric images of an example cancer subject obtained with compartmental modeling (CM) (gold standard), the conventional Patlak (CP), and the proposed Deep Patlak (DP) methods. **B** Image RMSE of the CP and DP methods for total-body K_i imaging averaged from the sixteen subjects.

**Fig. 3:**
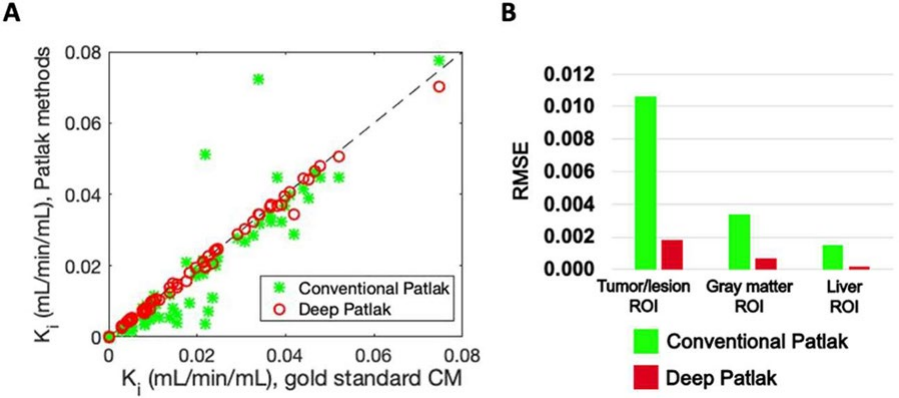
**A** Scatter plot of ROI-based quantifications of the conventional Patlak and the proposed Deep Patlak methods of 67 ROIs from the sixteen subjects. **B** ROI-based RMSE of the subjects using the Patlak methods.

**Ethics approval**

The study was approved by the IRB of University of California, Davis.

**References**

1. Carson RE. Tracer kinetic modeling in PET. In: Bailey D, Townsend D, Valk P, Maisey M, editors. Positron emission tomography. London, UK: Springer-Verlag; 2005. p. 127–59.

2. Patlak CS, Ronald GB, Joseph DF. Graphical evaluation of blood-to-brain transfer constants from multiple-time uptake data. JCBFM. 1983; 3(1): 1–7.

3. Wang Y, Li E, Cherry SR, Wang G. Total-body PET kinetic modeling and potential opportunities using deep learning. PET clinics. 2021; 16(4): 613–625.

4. Wang G, Nardo L, Parikh M, Abdelhafez YG, Li E, Spencer BA, Qi J, Jones T, Cherry SR, Badawi, RD. Total-Body PET Multiparametric Imaging of Cancer Using a Voxel-wise Strategy of Compartmental Modeling. J Nucl Med. 2021.

## O9 A fully-automated motion correction tool for dynamic total-body [18F]FDG-PET/CT studies: FALCON—fast algorithms for motion correction

### Sebastian Gutschmayer^1*^, Otto Muzik^2^, Anna Calabro^3^, Aaron Selfridge^3^, Josef Yu^1^, Lorenzo Nardo^3^, Yasser Abdelhafez^3^, Simon R Cherry^3^, Benjamin A Spencer^3^, Ramsey D Badawi^3^, Thomas Beyer^1^, and Lalith Kumar Shiyam Sundar^1^

#### ^1^Quantitative Imaging and Medical Physics (QIMP) Team, Center for Medical Physics and Biomedical Engineering, Medical University of Vienna, Vienna, Austria; ^2^Department of Pediatrics, Wayne State University School of Medicine, Children's Hospital of Michigan, Detroit, MI, USA; ^3^Department of Biomedical Engineering and Radiology, University of California-Davis, Davis, CA, USA

##### **Correspondence:** Sebastian Gutschmayer (sebastian.gutschmayer@meduniwien.ac.at)

*EJNMMI Physics* 2023, **10(1):**O9

**Background**

A fast, diffeomorphism-based methodology for automated motion correction of dynamic total-body [18F]FDG-PET/CT image volumes is presented.

**Materials and methods**

This is an ongoing study. To date, 10 dynamic, total-body (DTB) [18F]FDG-PET/CT datasets (60 min acquisition) from 2 PET/CT systems were included: 5 healthy controls (HC) acquired on an uEXPLORER and 5 HC on a Siemens Biograph Vision PET/CT system. A multi-scale diffeomorphic registration algorithm was used to align the DTB PET images. The last frame (55–60 min post-injection, p.i) was considered the reference, and all other frames corresponding to time-points > 4 min p.i were aligned to it. To quantitatively evaluate the performance of the motion correction, five volumes of interest (VOIs, brain, kidneys, liver, spleen, lung) were defined manually by a Physician in all time frames of the 10 DTB datasets. Relative overlap was calculated between the VOIs in all frames for each subject before and after motion correction using average symmetric surface distance (ASSD, mm). Likewise, the total activity was measured in the VOIs of all time frames before and after motion correction. The %-change in the ASSD and total intensity were reported as performance metrics.

**Results**

ASSD scores were reduced considerably for all VOIs (brain, kidneys, liver, spleen, lung) after motion correction with an average %-difference of 10, 26, 17, 10, and 2 across all subjects, respectively. The %-difference in the total activity of the VOIs before and after motion correction was < 5%, indicating the reasonable preservation of PET quantitative values. The average time to perform motion correction for a single TB-PET dataset (24 frames) was < 10 min.

**Conclusions**

A fast, fully-automated diffeomorphism-driven motion compensation approach was established and validated for [18F]FDG-PET total-body imaging. The proposed motion correction is clinically viable and can facilitate accurate parametric imaging in clinical routine.

**Ethics approval**

All data utilised in this study were acquired in accordance with the Declaration of Helsinki. Written information consent was obtained from all the subjects prior to examinations. Reference numbers: I1341792-18 and EK1907/2020.

## O10 Motion-frozen and motion-corrected total-body parametric reconstruction with deep learning-based data-driven methods

### Tiantian Li^a^, Xuezhu Zhang^a^, Zhaoheng Xie^a^, Edwin K Leung^a,b,c^, Benjamin A Spencer^a,b^, Guobao Wang^b^, Jinyi Qi^a*^

#### ^a^Department of Biomedical Engineering, University of California, Davis, CA, USA; ^b^Department of Radiology, University of California Davis Medical Center, Sacramento, CA, USA; ^c^UIH America, Inc., Houston, TX, USA

##### **Correspondence:** Jinyi Qi (jqi@lbl.gov)

*EJNMMI Physics* 2023, **10(1):**O10

**Objectives**

Long axial field of view PET scanners provide high sensitivity and total-body coverage for accurate quantification of a wide range of physiological parameters in vivo using dynamic scans. Physiological motions are inevitable during dynamic PET data acquisition and can cause image blurring and reduce quantitative accuracy. Here we present a deep learning-based data-driven method to obtain motion-frozen and motion-corrected total-body parametric images.

**Methods**

A one-hour dynamic ^18^F-FDG scan was acquired on a uEXPLORER PET/CT scanner. The last 30-min of the list-mode data was binned into 6 × 5-min frames. For each 5-min frame, respiratory gating was performed using an unsupervised deep neural network and k-means clustering method to generate 5 respiratory gates. The gate with the highest counts was selected to be the motion-frozen (reference) gate. The motion field between each gate and the reference gate was estimated. The estimated motion fields were used to generate phase-matched CT images for attenuation correction as well as to perform motion-compensated (MC) reconstruction. Finally, the reconstructed time activity curve at each voxel was fitted to the linear Patlak model to obtained parametric images with an input function extracted from a region over the descending aorta.

**Results**

Total-body parametric images showed that the proposed MC method can generate images with less motion artifact and better lesion detectability compared with the ungated image. Quantitatively the K_i_ values of the two liver lesions were 0.013/0.012 min^−1^ (ungated), 0.014/0.013 min^−1^ (MC), and 0.019/0.015 min^−1^ (reference), respectively. The coefficient of variation of a uniform liver region was 0.51 (ungated), 0.52 (MC), and 1.00 (reference), respectively. It demonstrated that the MC method provides a better lesion contrast versus background noise tradeoff than the other methods.

**Conclusions**

We have proposed a data-driven motion-frozen and motion-compensation method for total-body parametric imaging and showed its ability to reduce motion artifacts.

**Fig. 1:**
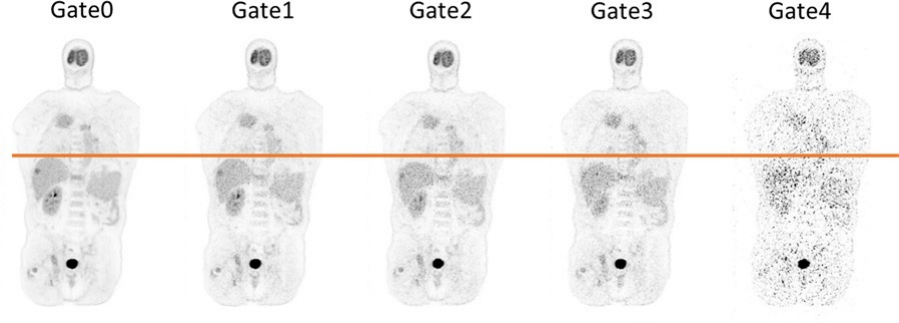
Sampled respiratory gated images. The horizontal line marks the liver dome position in Gate 0 (the reference gate). Substantial liver displacement can be observed in other gates. Gates 0–3 were from normal breathing and Gate 4 was from deep breathing.

**Fig. 2:**
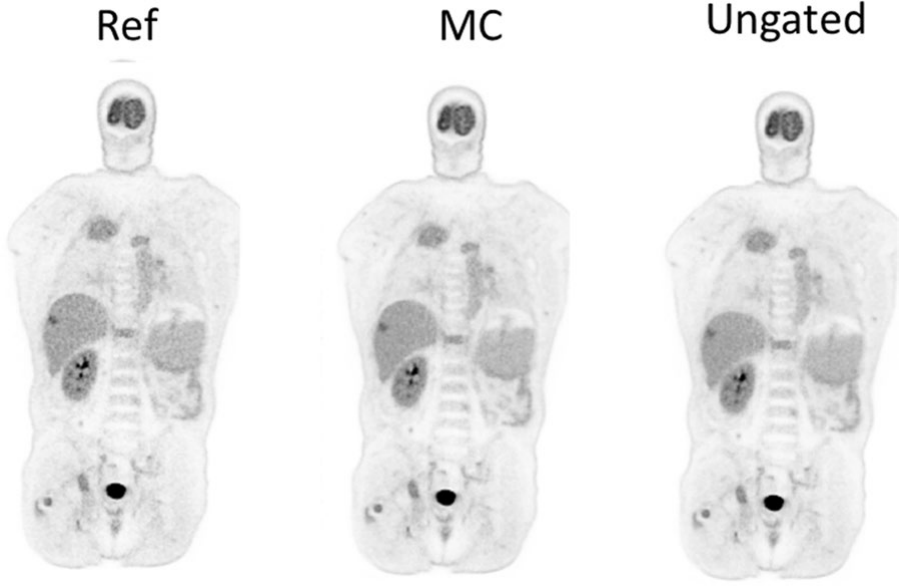
Reconstructed activity images with and without motion compensation (MC).

**Fig. 3:**
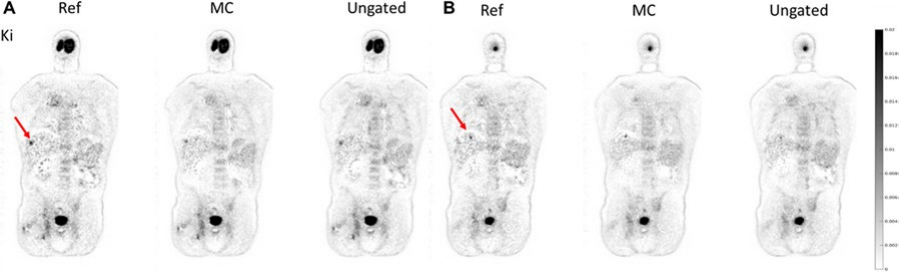
Reconstructed parametric images of Patlak K_i_ with and without motion compensation (MC) for the two lesions in the liver pointed by the red arrows.

## P1 A semi-monolithic DOI-encoding sub-200 ps detector tailored for total-body PET

### Florian Mueller^1*^, Stephan Naunheim^1^, Yannick Kuhl^1^, David Schug^1,2^, Volkmar Schulz^1,2,3,4^

#### ^1^Department of Physics of Molecular Imaging Systems, RWTH Aachen University, Aachen, Germany; ^2^Hyperion Hybrid Imaging Systems GmbH, Aachen, Germany; ^3^Physics Institute III B, RWTH Aachen University, Aachen, Germany; ^4^Fraunhofer Institute for Digital Medicine MEVIS, Aachen, Germany

##### **Correspondence:** Florian Mueller (florian.mueller@pmi.rwth-aachen.de)

*EJNMMI Physics* 2023, **10(1):**P1

**Background**

Clinical total-body PET systems with many detectors call for tailored approaches focusing on scalability regarding calibration effort, data processing, and cost. Compared to whole-body systems, DOI information gains additional importance, improving sensitivity and reducing parallax error [1]. We present a detector concept based on semi-monolithic slabs fulfilling these needs.

**Materials and methods**

The detector consisted of 8 monolithic LYSO slabs of dimensions 3.9 × 32 × 19 mm^3^ read out by a 64-channel digital SiPM photosensor (DPC3200-22-44, PDPC). Calibration data for position estimation were acquired using a fan-beam collimator, here extended to simultaneous irradiation with multiple slits to push the calibration time to minutes. For energy and timing calibration, flood measurements without collimator were performed. The position estimation in the detector’s monolithic and DOI direction was based on the machine learning technique gradient tree boosting (GTB) [2, 3]. We have recently demonstrated a high-throughput implementation of GTB to demonstrate the computational feasibility for large-scale systems [4]. The energy calibration and calculation accounted for the position-dependent detector response. We established an analytical and GTB-based timing calibration and used them for a first-photon trigger. While the analytical timing calibration corrects for electronic and optical time skews, the GTB algorithm was applied on top of the analytical calibration.

**Results**

We achieved a positioning performance measured as mean absolute error (MAE) of 1.27 mm and 1.94 mm for DOI with an energy resolution of 11.3%. The analytical timing calibration led to a CRT of 228 ps which significantly improved to 198 ps utilizing the GTB-based timing calibration. All results are reported around the photopeak without applying any additional filters.

**Conclusions**

The presented detector offers an attractive performance with sub-200 ps CRT including DOI-encoding. The optimized calibration methods and high-throughput GTB-based data processing lay the foundations for application in total-body PET systems.

**References**

1. Schmall JP, Karp JS, Werner M, Surti S. Parallax error in long-axial field-of-view PET scanners—a simulation study. Phys Med Biol. 2016;61:5443–55.

2. Mueller F, Schug D, Hallen P, Grahe J, Schulz V. Gradient Tree Boosting-Based Positioning Method for Monolithic Scintillator Crystals in Positron Emission Tomography. IEEE Trans Radiat Plasma Med Sci. 2018;2:411–21.

3. Mueller F, Schug D, Hallen P, Grahe J, Schulz V. A Novel DOI Positioning Algorithm for Monolithic Scintillator Crystals in PET Based on Gradient Tree Boosting. IEEE Trans Radiat Plasma Med Sci. 2019;3:465–74.

4. Wassermann C, Mueller F, Dey T, Lambertus J, Schug D, Schulz V, et al. High throughput software-based gradient tree boosting positioning for PET systems. Biomed Phys Eng Express. 2021;7:055023.

## P2 Impact of the acceptance angle on image quality for the biograph vision quadra total body PET/CT scanner

### Fabian P Schmidt^1,2^, Ivo Rausch^3^, Mohamad Altllawi^1^, Julia G Mannheim^2,4^, Sebastian v Beschwitz^1^, Jürgen Kupferschläger^1^, Christian la Fougère^1,4^

#### ^1^Department of Nuclear Medicine and Clinical Molecular Imaging, University hospital Tuebingen, Tuebingen, Germany; ^2^Werner Siemens Imaging Center, Department of Preclinical Imaging and Radiopharmacy, Eberhard-Karls University Tuebingen, Tuebingen, Germany; ^3^QIMP Team, Center for Medical Physics and Biomedical Engineering, Medical University of Vienna, Vienna, Austria; ^4^Cluster of Excellence iFIT (EXC 2180) “Image Guided and Functionally Instructed Tumor Therapies”, University of Tuebingen, Tuebingen, Germany

##### **Correspondence:** Fabian P Schmidt (f.schmidt@med.uni-tuebingen.de)

*EJNMMI Physics* 2023, **10(1):**P2

**Background**

The sensitivity of the (clinical) reconstruction of the Siemens Biograph Vision Quadra is currently being limited by the maximum ring difference (MRD) of 85. Aim of this study was to evaluate the impact of MRD322 (maximum angle) on the image quality (IQ) for different isotopes and scan durations.

**Materials and methods**

A NEMA IQ phantom was measured (^18^F, ^64^Cu and ^68^ Ga, SBR 8:1, 2 kBq/ml) at different axial positions. Multiple frame durations (600 s, 300 s, 120 s, 30 s and 15 s) and reconstructions (Siemens e7tools, PSF-TOF, 4i5s, allpass) for MRD85 and MRD322 were evaluated. IQ was qualitatively evaluated (Fig. 1,2), with determining the contrast recovery coefficient (CRC) and the coefficient of variation (CV) for a background VOI and the CV in a VOI placed in the liver of a patient (3 MBq/kg [^18^F]-FDG, 1 h p.i.).

**Results**

Reconstruction with MRD322 improved IQ and CV for the central FOV, e.g. CV changed from 19.8% (MRD85) to 14.4% (MRD322) for a 120 s ^18^F acquisition (Fig. 3). The calculated CRCs remained stable irrespective of the used MRD. A scan time of 120 s resulted in the same CRCs as 600 s for all isotopes, e.g. for the 17 mm sphere: 74.4% (^18^F) and 60.1% (^68^Ga).

For the patient data, the CV ranged from 11.5 to 8.9% (300 s) to 47.4% and 35.2% (15 s) for MRD85 and MRD322, respectively. We found an average improvement of 24.7% for the CV with MRD322 for all scan durations.

**Conclusion**

With MRD322, a reduction in image noise was achieved for the central FOV. Thus, MDR322 enables a reduction in scan time while preserving IQ.

**Fig. 1:**
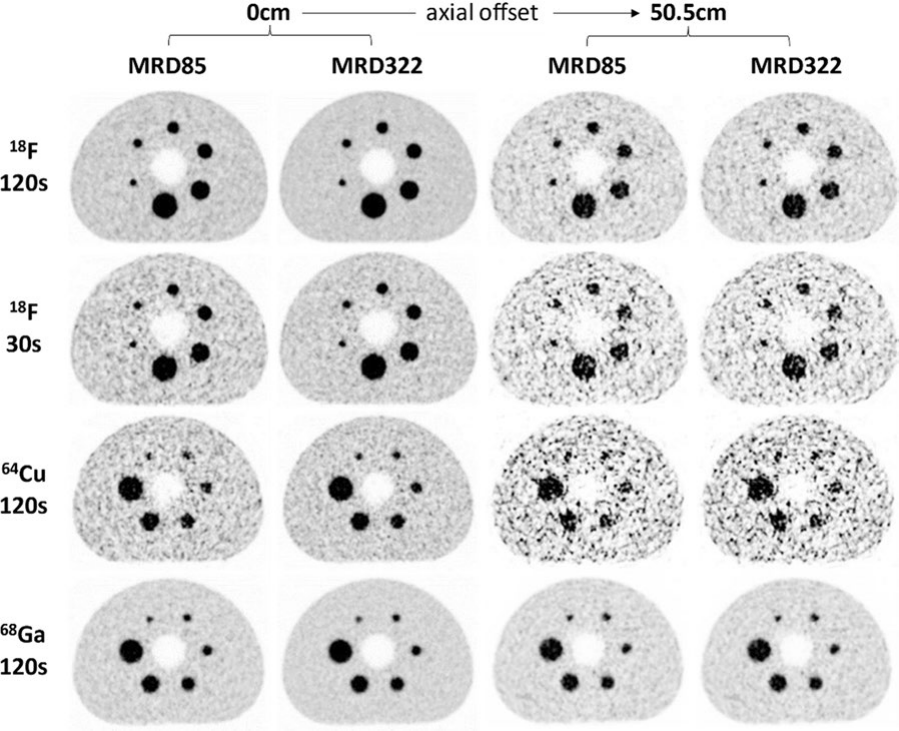
IQ Phantom for different isotopes, scan times, axial positions and acceptance angle.

**Fig. 2:**
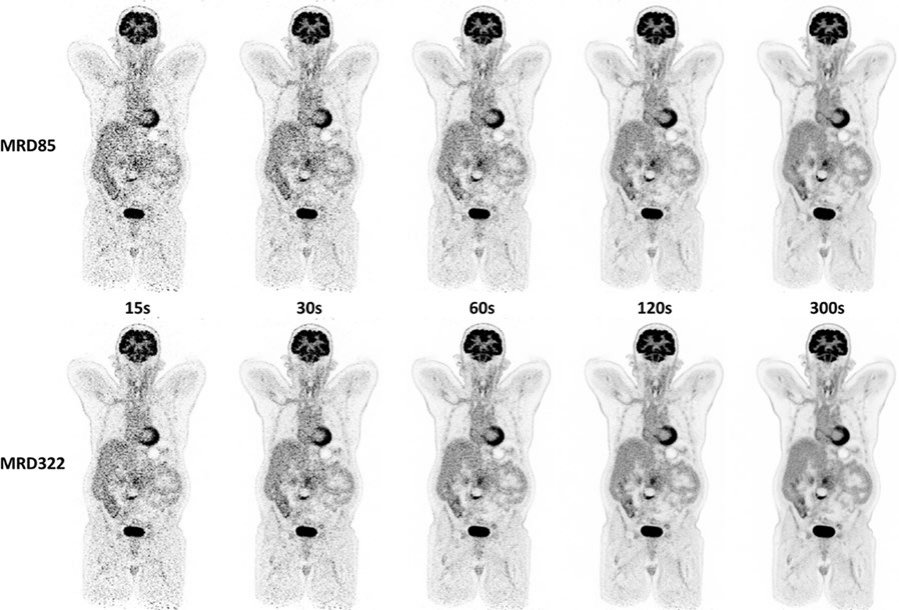
[^18^F]FDG IQ maintained for shorter scan duration for MRD322, e.g. CV of 17.3% (MRD85, 120 s) and 18.0% (MRD322, 60 s).

**Fig. 3:**
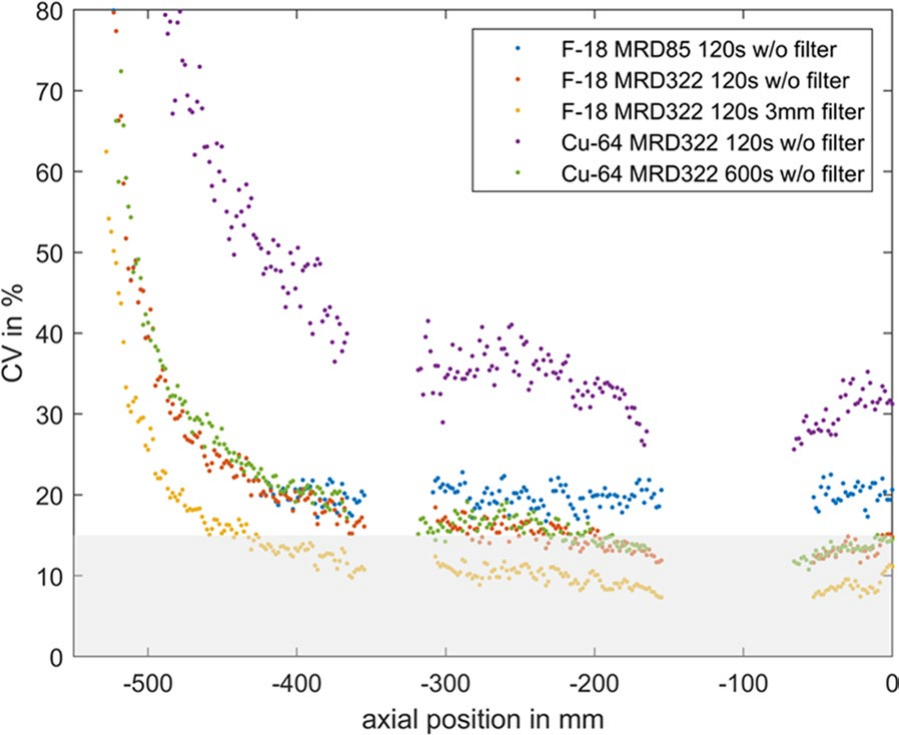
Phantom CV along the axial position.

## P3 High sensitivity molecular imaging: a total-body PET with TOF and DOI capabilities

### Andrea Gonzalez-Montoro^1*^, Marta Freire^1^, David Sanchez^1^, John Barrio^1^, Neus Cucarella^1^, Gabriel Cañizares^1^, Santiago Jimenez-Serrano^1^, Luis F. Vidal^1^, Julio Barbera^2^, Karel Diaz^2^, Constantino Morera-Ballester^2^, Jorge Alamo^2^, Jose M. Benlloch^1^, and Antonio J. Gonzalez^1^

#### ^1^Instituto de Instrumentación para Imagen Molecular (I3M), Centro Mixto CSIC—Universitat Politècnica de València, 46022 Valencia, Spain; ^2^Oncovision S.A., 46022 Valencia, Spain

##### **Correspondence:** Andrea Gonzalez-Montoro (andrea.gm@i3m.upv.es)

*EJNMMI Physics* 2023, **10(1):**P3

**Background**

Total-Body PET scanners have shown potential to provide high sensitivity multiparametric imaging. However, building such a system imposes technological challenges: providing accurate reconstructed images within a short time-frame requires TOF capabilities and accurate DOI.

The present abstract summarizes the preliminary performance of our detector design suitable for the TB-PET with 70 cm axial coverage that is being built at the I3M.

**Material and methods**

The detector design comprises 4 mini-modules, each composed of 8 slabs of 25.8 × 3.1 × 20 mm^3^ LYSO crystals coupled to arrays of 8 × 8 SiPMs [1]. The slab configuration allows to exploit the advantages present in both pixelated and monolithic designs as described in [2].

The detector includes a readout electronics to reduce the number of signals from N^2^ to 2N without impacting performance.

Coincidence data was acquired moving a reference detector together with a slit collimator along the monolithic and DOI directions. The *x*- and *DOI*- photon interaction positions were estimated using a NN. This data was also used to analytically evaluate the energy resolution and the CTR.

**Results**

Figure 1 show examples of the flood maps obtained, with the multiplexed readout already implemented, when homogenously irradiating the detector (left) and when the slit was placed at the center of the module (right). All slabs are identified. Figure 2 left and right, reports the average spatial and DOI resolution values achieved for the module using the NN after and before implementing the multiplexing circuitry, respectively.

An average energy resolution of 13.4 ± 0.9% was reported for the entire module. Figure 3 summarizes the average CTR values for each mini-module (left) and compares the CTR values before and after implementing the reduction readout circuit (right).

**Conclusions**

These promising results prove that our detector meets the requirements for a TB-PET scanner as outlined at the beginning of this abstract.

**Fig. 1:**
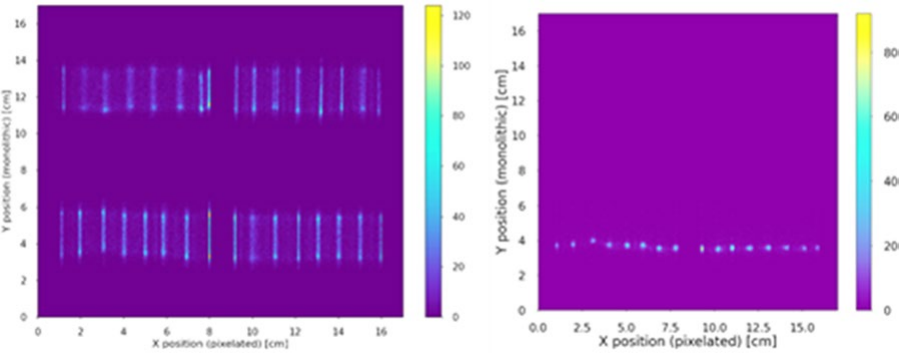
Floodmaps of the module homogenously irradiated, and with the slit centered in the mini-modules.

**Fig. 2:**
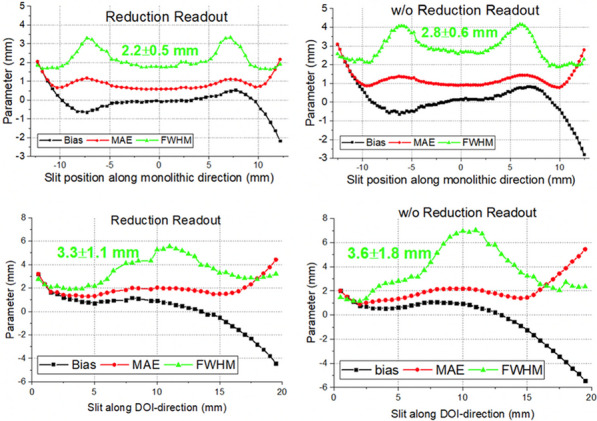
Spatial and DOI resolution values for the module using the NN after and before implementing the multiplexing circuitry.

**Fig. 3:**
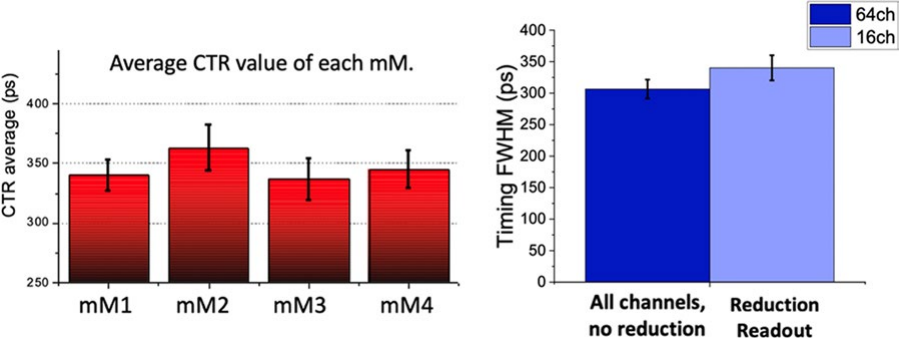
CTR values for the minimodules after and before implementing the multiplexing circuitry.

**References**

1. Cucarella N, Barrio J, Lamprou E, Valladares C, Benlloch J M, Gonzalez A J. Timing evaluation of a PET detector block based on semi-monolithic LYSO crystals. Med. Phy. 2021; 48(12): 8010–8023.

2. Gonzalez-Montoro A, Gonzalez A J, Pourashraf S, Miyaoka R S, Bruyndonckx P, Chinn G, Pierce L A, Levin C S. Evolution of PET Detectors and Event Positioning Algorithms Using Monolithic Scintillation Crystals. IEEE Trans. Rad. Plasma Med. Sci. 2021; 5(3): 282–305.

## P4 Image quality variation along the axial field-of-view of the Biograph Vision Quadra total-body PET/CT system for 18-F

### Ivo Rausch^1^*, Julia G Mannheim^2,3^, Jürgen Kupferschläger^4^, Christian la Fougère^3,4^ and Fabian P Schmidt^2,4^

#### 1. QIMP Team, Center for Medical Physics and Biomedical Engineering, Medical University of Vienna, Vienna, Austria; 2. Werner Siemens Imaging Center, Department of Preclinical Imaging and Radiopharmacy, Eberhard-Karls University Tuebingen, Tuebingen, Germany; 3. Cluster of Excellence iFIT (EXC 2180) “Image Guided and Functionally Instructed Tumor Therapies”, University of Tuebingen, Tuebingen, Germany; 4. Department of Nuclear Medicine and Clinical Molecular Imaging, University hospital Tuebingen, Tuebingen, Germany

##### **Correspondence:** Ivo Rausch (ivo.rausch@meduniwien.ac.at)

*EJNMMI Physics* 2023, **10(1):**P4

**Background**

The nominal axial field-of-view (aFOV) of total-body (TB) PET/CT systems may clinically not be used effectively due to the inherently reduced sensitivity at the axial ends of the PET. This study assessed the noise and image quality across the aFOV of the Vision Quadra TB PET/CT system.

**Methods**

NEMA image quality (IQ) phantom acquisitions were performed across four positions along the aFOV of the Biograph Vision Quadra PET/CT system (Siemens Healthineers). PET raw data were rebinned to simulate 10 min, 2 min, 30 s and 15 s acquisition time and reconstructed using standard settings with different filter settings. Axial noise behaviour was assessed by slice wise calculating the coefficient-of-variation. Axial IQ changes were investigated by means of image contrast. A clinical useful axial FOV was estimated based on a maximum CV of 15%.

**Results**

Image noise and contrast were stable within the central 80 cm of the aFOV. Outside this central area, image contrast variability as well as image noise increased (Fig. 1). This degradation of IQ was in particular evident for short acquisition times. At 10 min acquisition time and without filtering, the useful axial FOV was 100 cm. For a 2 min acquisition time, an aFOV with image noise below 15% was only achievable using Gaussian filtering with axial extents of between 83 to 103 cm when going from 2 to 6 mm FWHM, respectively.

**Conclusion**

Image noise increases substantially towards the ends of the aFOV. However, good IQ in compliance with generally accepted benchmarks is achievable for an axial FOV of > 90 cm. When accepting higher image noise or using dedicated protocol settings, a useful axial FOV of around 1 m can be achieved.

**Fig. 1:**
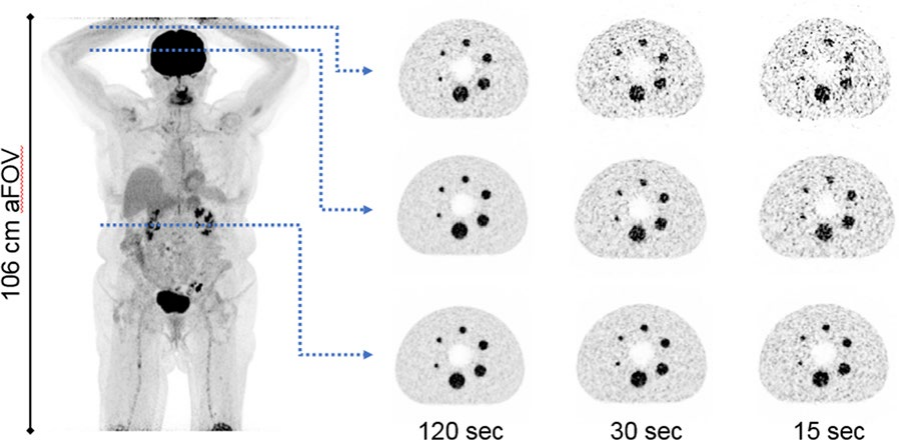
Central slice of the IQ phantom at different axial position in the FOV and for different acquisition times.

## P5 Yttrium-90 post-radioembolization protocol optimization with a long axial field-of-view positron emission tomography

### Konstantinos G. Zeimpekis^1^*, Lorenzo Mercolli^1^, George Prenosil^1^, Hasan Sari^1,2^, Kuangyu Shi^1,3^, Axel Rominger^1^

#### ^1^Dept. Nuclear Medicine, Inselspital, Bern University Hospital, University of Bern, Switzerland; ^2^Advanced Clinical Imaging Technology, Siemens Healthcare AG, Lausanne, Switzerland; ^3^Dept. Informatics, Technical University of Munich, Germany

##### **Correspondence:** Konstantinos G. Zeimpekis (konstantinos.zeimpekis@insel.ch)

*EJNMMI Physics* 2023, **10(1):**P5

**Background**

The purpose of the study was to investigate possible optimization based on number of iterations used for post-radioembolization positron emission tomography (PET) yttrium-90 (Y-90) scans, with a long axial field-of-view (AFOV) scanner.

**Materials and methods**

A whole-body PET scanner with an AFOV of 106 cm was used. The parameters are summarized in Table 1. The ultra-high sensitivity (UHS) mode utilizing the complete ring difference with improved 3D scatter correction was used. A VOI was placed on the liver background and on the lesion. The mean and SD values were extracted.

**Results**

SNR for 2 iterations was 14.1, 16.5, 17.9 for 5, 10 and 20 min while for 4 iterations it was 10.4, 12.7, 14.2 respectively (Fig. 1). SD was 30%, 27% and 23% lower for 2 iterations 5, 10 and 20 min, compared to 4 iterations. The background liver VOI mean did not show differences for 2 and 4 iterations.

**Conclusion**

2 iterations produce lower noise and improved SNR for post-radioembolization Y-90 than 4 iterations in long AFOV PET scanner.

**Table 1:** Acquisition and reconstruction parameters.

Parameter	
Age of patient	73
Y-90 activity (GBq)	1.2
Reconstruction	
Algorithm	3D OSEM
Matrix	220 × 220
TOF + PSF	Yes
Iterations	2, 4
Subsets	5
Gaussian FWHM (mm)	2

**Fig. 1:**
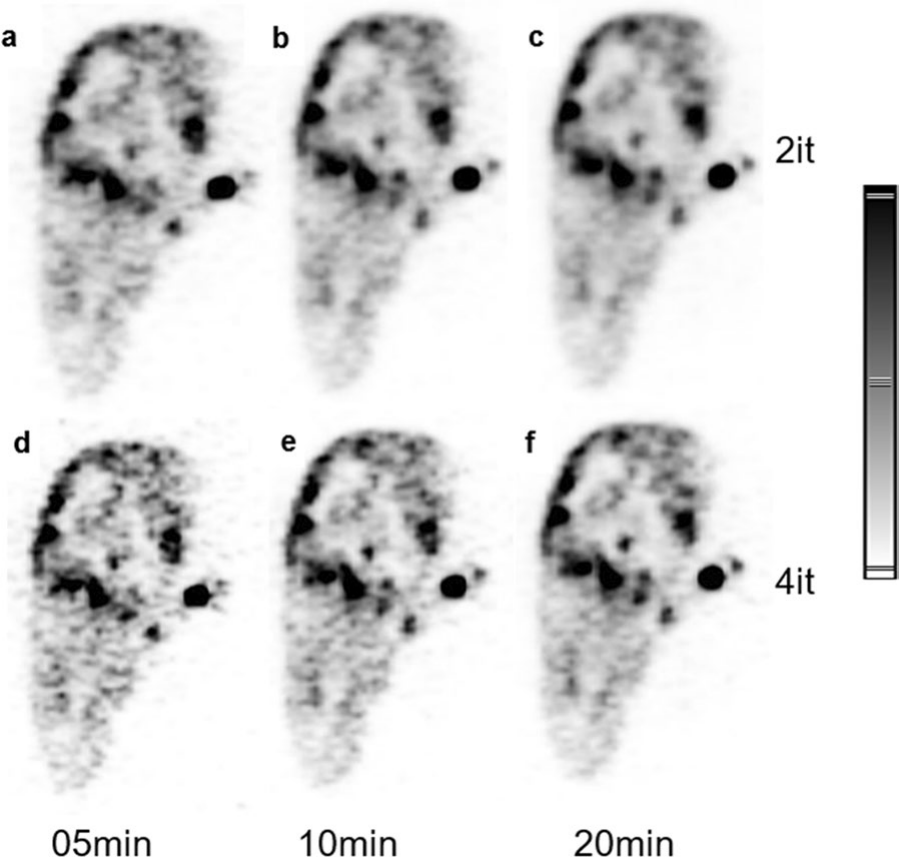
Coronal liver MIPs after Y-90 radioembolization. **a**–**c** reconstructed with 2 iterations, **d**–**f** reconstructed with 4 iterations. After list-mode data rebinning, (**a**, **d**), (**b**, **e**) and (**c**, **f**) reconstructed with 5, 10 and 20 min simulated acquisition times.

## P6 Human skeletal energy networks identified with positron emission tomography imaging

### Karla J. Suchacki^1^*, Rucha Ronghe^1^, David Dye^2^, Catriona Wimberley^2,3^, Simon Cherry^4^, Ramsey Badawi^4^, Lorenzo Nardo^4^, Elizabeth Li^4^, Benjamin Spencer^4^, Adriana A. S. Tavares^1,2^

#### ^1^University/British Heart Foundation Centre for Cardiovascular Science, University of Edinburgh, UK; ^2^Edinburgh Imaging, University of Edinburgh, UK; ^3^Centre for Clinical Brain Sciences, University of Edinburgh, UK; ^4^EXPLORER Molecular Imaging Center, UC Davis, USA

##### **Correspondence:** Karla J. Suchacki (karla.suchacki@ed.ac.uk)

*EJNMMI Physics* 2023, **10(1):**P6

**Background**

Total-body dynamic *positron emission tomography* computerised tomography (PET/CT) lends itself to deciphering complex biological processes and interactions. Recently, we found that different bones within the murine skeleton have a unique glucose metabolism and form a complex metabolic network [1]. Thus we hypothesised that *(1) individual bones have different glucose metabolism and form complex skeletal metabolism networks in humans.* We also wanted to investigate how *(2) changes in multi-bone glucose metabolism in humans associate with cancer disease development and progression*.

**Materials and methods**

(1) 13 (4 male, 9 female) volunteers (age 49.7 ± 13.4y, BMI 29.1 ± 5.9 kg/m^2^) underwent dynamic total-body [^18^F]FDG-PET/CT scanning (uEXPLORER (United Imaging Healthcare, Shanghai, China; ethics:IRB). (2) 27 (19 male, 8 female) stage IIB lung cancer patients (age 59.6 ± 9.2y, BMI 24.9 ± 4.4 kg/m^2^) underwent conventional static [^18^F]FDG-PET/CT scanning (ethics:ACRIN6668) [2,3,4]. Reconstructed PET/CT images were quantified using PMOD 3.117 (PMOD Technologies, Switzerland). Bone volumes of interest were segmented as previously described [1]. (1) Time-activity curves (TACs) were generated and SUVs were calculated for each time point (0–60 min), the average SUV for the last 3 frames were generated. (2) Skeletal energy networks were generated using Graphia (Kajeka, UK)^[1]^. SUV at equilibrium was extracted for static PET images and CT Hounsfield Units were extracted for each bone (1&2).

**Results**

Skeletal [^18^F]FDG uptake was equal or higher in humans compared to mice, with higher uptake in bones of the axial skeleton (Fig. 1). [^18^F]FDG murine networks translated to healthy humans. Skeletal [^18^F]FDG uptake was sex-dependent in healthy volunteers (Fig. 2), however this sex effect was not observed in cancer patients. PET network analysis clustered lung cancer patients into two clusters translating to a two-tiered survival plot (Fig. 3).

**Conclusions**

Network analysis could be used to identify new physiological and pathological tissue interactions beyond individual bones metabolism and to stratify patients with ability to predict patient survival.

**Fig. 1:**
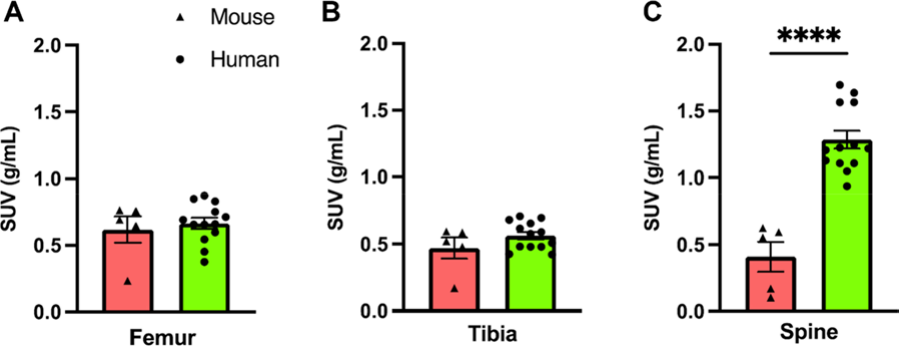
[^18^F]FDG uptake in healthy human subjects versus healthy mice. Data are mean ± SEM * **** (*p* < 0.0001).

**Fig. 2:**
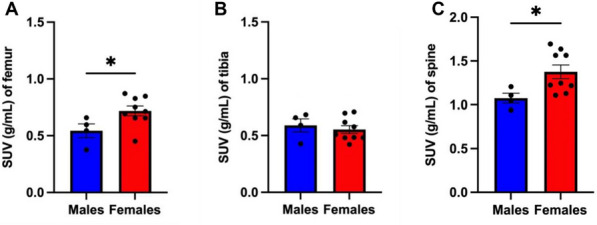
[^18^F]FDG SUV in healthy human males versus healthy human females. Data are mean ± SEM * (*p* < 0.05).

**Fig. 3:**
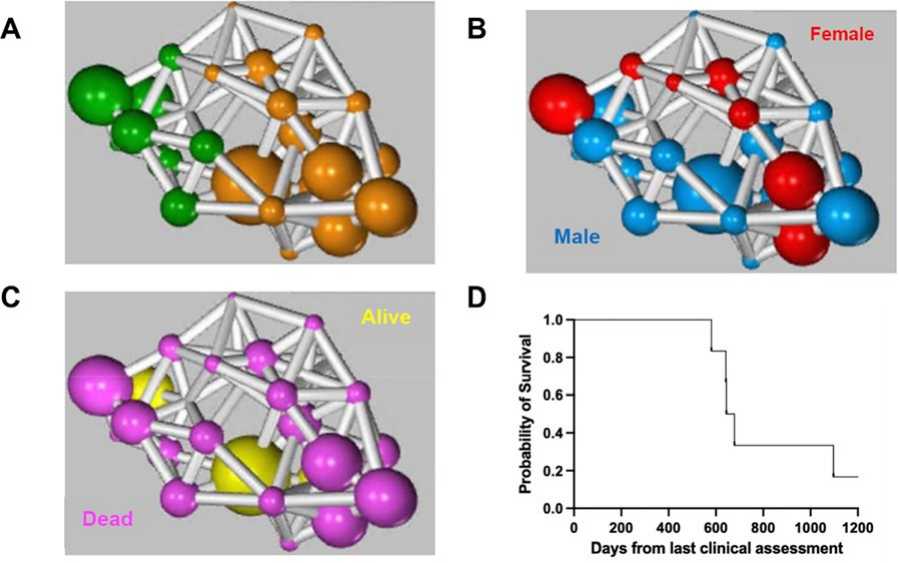
Skeletal energy networks identified with positron emission tomography imaging. Functional networks and survival plot identified by network analysis of lung cancer patients (**A**–**D**). (**A**) Clustered network from PET data where node sizes represent days since last clinical assessment (kNN value of 5). (**B**) Clustered network from PET data where node colours represent males and females (kNN value of 5). (**C**) Clustered network from PET data where node colours represent vital status (kNN value of 5). (**D**) Survival plot of lung cancer patients with days from last clinical assessment on x axis and probability of survival on y axis.

**References**

[1] Suchacki KJ, Alcaide-Corral CJ, Nimale S, Macaskill MG, Stimson RH, Farquharson C, Freeman TC, Tavares AAS. A Systems-Level Analysis of Total-Body PET Data Reveals Complex Skeletal Metabolism Networks in vivo. Front Med (Lausanne). 2021 Sep 20;8:740615.

[2] Machtay M, Duan F, Siegel BA, Snyder BS, Gorelick JJ, Reddin JS, Munden R, Johnson DW, Wilf LH, DeNittis A, Sherwin N, Cho KH, Kim SK, Videtic G, Neumann DR, Komaki R, Macapinlac H, Bradley JD, Alavi A. Prediction of survival by [18F]fluorodeoxyglucose positron emission tomography in patients with locally advanced non-small-cell lung cancer undergoing definitive chemoradiation therapy: results of the ACRIN 6668/RTOG 0235 trial. J Clin Oncol. 2013 Oct 20;31(30):3823–30.

[3] Kinahan, P., Muzi, M., Bialecki, B., Herman, B., & Coombs, L. (2019). Data from the ACRIN 6668 Trial NSCLC-FDG-PET [Data set]. The Cancer Imaging Archive. https://doi.org/10.7937/tcia.2019.30ilqfcl

[4] Clark K, Vendt B, Smith K, Freymann J, Kirby J, Koppel P, Moore S, Phillips S, Maffitt D, Pringle M, Tarbox L, Prior F. The Cancer Imaging Archive (TCIA): maintaining and operating a public information repository. J Digit Imaging. 2013 Dec;26(6):1045–57.

## P7 SAFIR: Total-body small animal PET inserts for quantitative kinetic studies of fast processes

### Pascal Bebié^1*^, Robert Becker^1^, Volker Commichau^1^, Jan Debus^1^, Günther Dissertori^1^, Lubomir Djambazov^1^, Afroditi Eleftheriou^2^, Peter Fischer^3^, Parisa Khateri^1^, Werner Lustermann^1^, Christian Ritzer^1^, Michael Ritzert^3^, Ulf Röser^1^, Charalampos Tsoumpas^4^, Geoffrey Warnock^5^, Bruno Weber^2^, Matthias T Wyss^2^, Agnieszka Zagozdzinska-Bochenek^1^

#### ^1^Institute for Particle Physics and Astrophysics, ETH Zurich, 8093 Zurich, ZH, Switzerland; ^2^Institute for Pharmacology and Toxicology, University of Zürich, 8057 Zurich, ZH Switzerland; ^3^Institute of Computer Engineering, University Heidelberg, 69120 Heidelberg, BW, Germany; ^4^Medical Imaging Center, Department of Nuclear Medicine and Molecular Imaging, University Medical Center Groningen, University of Groningen, 9713 GZ Groningen, GR, The Netherlands; ^5^Centre for Molecular Cardiology, University of Zürich, 8952 Schlieren, ZH, Switzerland

##### **Correspondence:** Pascal Bebié (bebiep@phys.ethz.ch)

*EJNMMI Physics* 2023, **10(1):**P7

The Small Animal Fast Insert For MRI (SAFIR) collaboration is developing a Positron Emission Tomography (PET) insert for preclinical studies inside a Bruker BioSpec 70/30 USR 7T Magnetic Resonance Imaging (MRI) system. It aims at enabling truly simultaneous quantitative total-body PET/MRI acquisitions in mice and rats at injected activities reaching 500 MBq. The high count rate allows to capture fast kinetics in image time frames of $\le$ 5 s in length [1].

The detector design features a dodecagonal arrangement of readout and powering components, all fully incorporated and shielded within an annular cylindrical carbon fiber structure inside the magnetic field of the MRI (see Figs. 1 and 2). The detector head centers on Lutetium-Yttrium-Oxyorthosilicate (LYSO) crystals, one-to-one coupled to the elements of Hamamatsu SiPM-arrays (TVS MPPC13361-2050), which are read out by Application-Specific Integrated Circuits (ASICs).

A first insert, SAFIR-I, using the PETA6SE ASIC [2,3], featuring an axial Field-of-View (FOV) of 54.2 mm and achieving 209 ps and 12.41% Coincidence Resolving Time (CRT) and energy resolution, respectively, has successfullly been commissioned and used for the in vivo study of a rat brain at an injected activity of 315 MBq [1]. A full characterisation of this detector according to the NEMA NU4-2008 standard [4] is currently ongoing. Based on preliminary results, we expect an average system sensitivity of ca. 1.5% and a spatial resolution of 2 mm in the center of the FOV.

Simultaneously, we expect to complete the construction of the SAFIR-II PET insert by the end of 2022. It will feature both an updated detector head with improved PETA ASIC (PETA8) and minimized light crosstalk [5] and a longer axial coverage of 145 mm for total-body rat imaging. This insert is expected to extend on SAFIR-I’s capabilities by reaching an average system sensitivity of ca. 4% and enabling time frames of 1 s duration [*Cf.* 1].

**Fig. 1:**
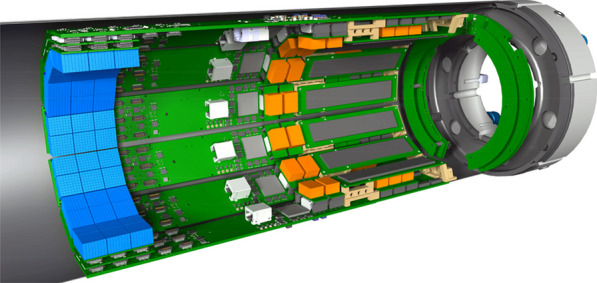
Rendering of the SAFIR-I PET insert showing seven of the twelve sections and one half of the outer carbon fiber shell structure. The entire detector head features 4320 readout channels/LYSO crystals (2.1 mm × 2.1 mm × 13 mm). The inner carbon fiber cylinder & support structure is not depicted to offer unrestricted view on the electronics arangement.

**Fig. 2:**
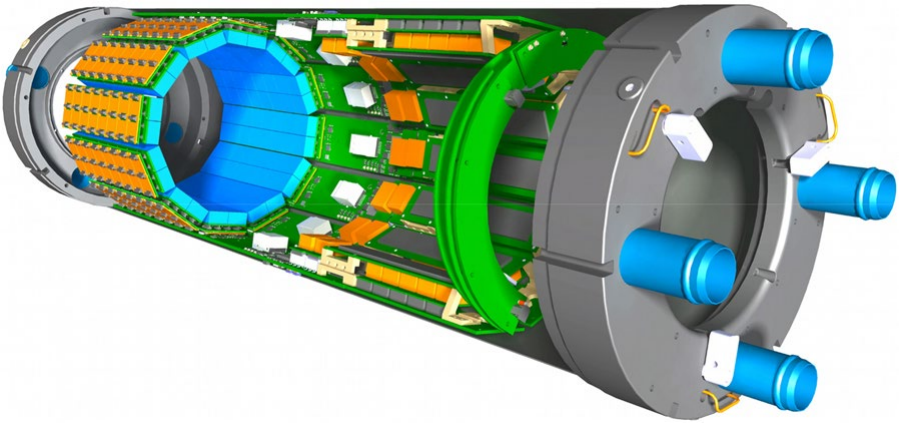
Rendering of the SAFIR-II PET insert showing seven complete detector sections, the entire dodecagonal detector head with 11,520 readout channels/LYSO crystals (2 mm × 2 mm × 13 mm) and the partial outer carbon fiber shell structure. The inner carbon fiber cylinder & support structure is not depicted to offer unrestricted view on the electronics arangement.

**Ethics approval**

The in vivo rat brain study was approved under license ZH228/2019 by the local veterinary authorities, conforming to the guidelines of the Swiss Animal Protection Law, Veterinary Office, Canton Zurich (Act of Animal Protection 16 December 2005, and Animal Protection Ordinance 23 April 2008).

**References**

1. Bebié P, Becker R, Commichau V, Debus J, Dissertori G, Djambazov L, et al. SAFIR-I: Design and Performance of a High-Rate Preclinical PET Insert for MRI. Sensors, 2021 Oct;21(21), 7037.

https://doi.org/10.3390/s21217037

2. Dohle R, Sacco I, Rittweg T, Friedrich T, Henning G, Goßler J, et al. LTCC-Based Highly Integrated SiPM Module with Integrated Liquid Cooling Channels for High Resolution Molecular Imaging. J. Microelectron. Electron. Packag. 2018, 15, 86–94.

3. Sacco I, Dohle R, Fischer P, Piemonte C, Ritzert M. A compact, high-density gamma-detection module for Time-of-Flight measurements in PET applications. Nucl. Instruments Methods Phys. Res. Sect. A Accel. Spectrometers Detect. Assoc. Equip. 2016, 824, 233–236.

4. NEMA. Performance Measurements of Small Animal Positron Emission Tomographs (PETs); NEMA Standards Publication: Rosslyn, VA, USA, 2008.

5. Bebié P, Lustermann W, Ritzer C, Wixinger R, Dissertori G. Effects of inter-crystal optical separation layers on unwanted light crosstalk and on performance parameters of the SAFIR PET/MR scanner. Poster session presented at: IEEE NSS MIC; 2021 Oct 16–23; Yokohama, Japan.

## P8 Positron emission particle tracking (PEPT) for biomedical applications with total-body PET: implementation and characterisation in preclinical PET

### Laurence Vass^1^*, Agathe Bricout^1^, David J Parker^2^, Juan Pellico^1^, Rafael T M de Rosales^1^, Paul K Marsden^1^

#### ^1^School of Biomedical Engineering and Imaging Sciences, King’s College London, London, SE7 1EH, UK; ^2^School of Physics and Astronomy, The University of Birmingham, Edgbaston, Birmingham B15 2TT, UK

##### **Correspondence:** Laurence Vass (Laurence.vass@kcl.ac.uk)

*EJNMMI Physics* 2023, **10(1)**:P8

**Background**

Positron emission particle tracking (PEPT) is a powerful tool for imaging opaque mediums and to date has a myriad of industrial applications. PEPT techniques enable tracking of individually labelled radioactive particles with potential to offer valuable new information not available from traditional PET imaging; yet there have been very few examples in biomedical applications. Total body PET could represent a unique platform to exploit PEPT; for example by enabling tracking individual cells throughout the body, or to measure blood flow throughout the vasculature [1]. Here, we characterise the performance of PEPT on a preclinical PET scanner using a programmable robotic device with point sources of different activity and moving in complex trajectories with varying speeds.

**Materials and methods**

We modified an FLSUN-Q5 3D printer to hold radioactive sources (see Fig. 1) in the field of view of the Mediso NanoScan PET/CT. Various trajectories were programmed using G-code, PEPT trajectories were reconstructed using the Birmingham method [2]. Point sources included a Na-22 sealed source (0.25 mm active diameter) and Ga-68 silica alginate microbeads (diameter–4 mm).

**Results**

Complex trajectories could be visualised at low levels of activity (= 55 kBq) (Fig. 2). Estimated distances had a small underestimation bias (see Fig. 3), the positional uncertainty of a stationary point source was 0.1 mm using 1000 lines of response. The positional error of a 55 kBq Na-22 point source was 1.99 mm when moving at a speed of 0.97cms^−1^, the error increased with point source speed.

**Conclusions**

We implemented a PEPT processing pipeline on a preclinical PET/CT scanner. We were able to accurately track a point source at different speeds with low levels of radioactivity. We speculate that total-body PET will empower a variety of PEPT applications for biomedical research. Advancements in radiolabelling of microparticles will enable further preclinical in-vivo evaluation [3].

**Fig. 1:**
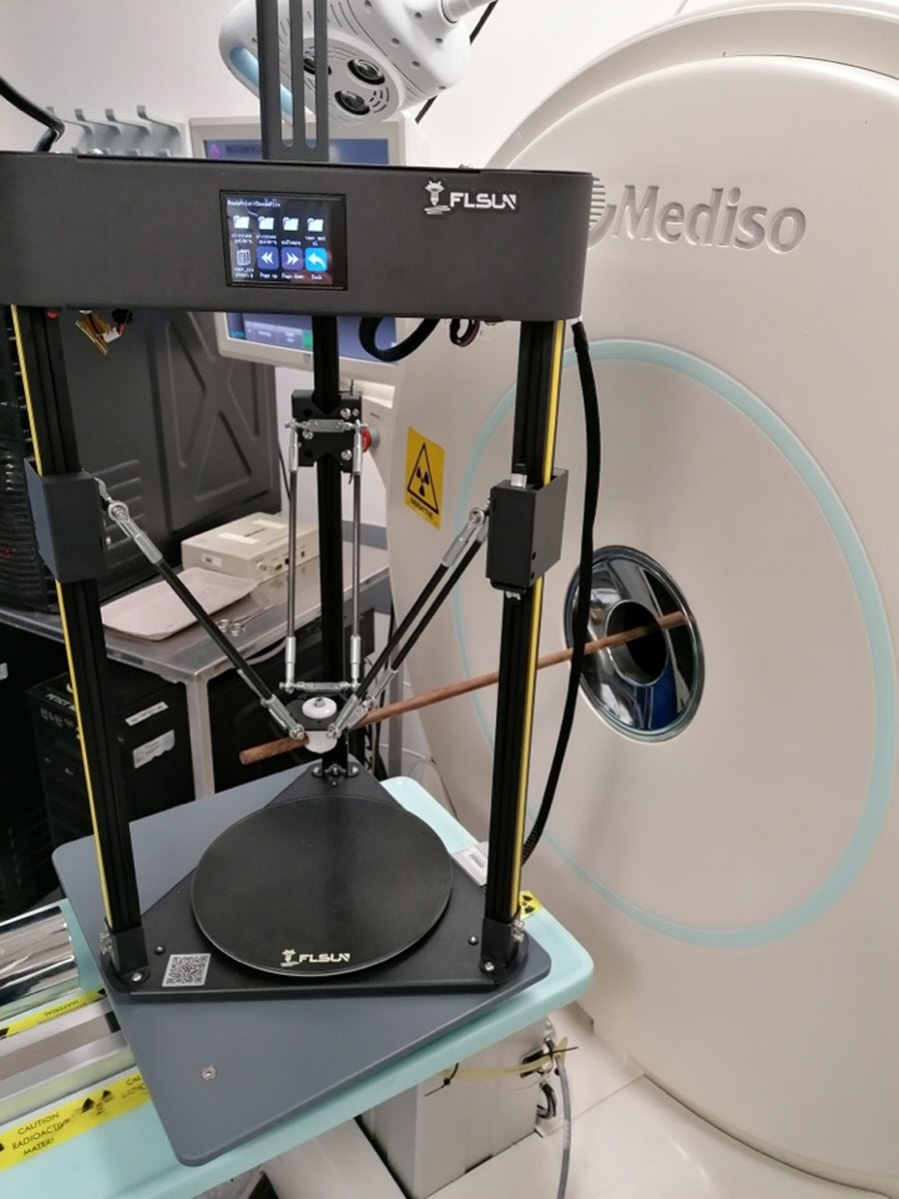
Experimental set-up for PEPT acquisitions with 3D printer. The printer can be programmed to perform different trajectories with accurate positioning.

**Fig. 2:**
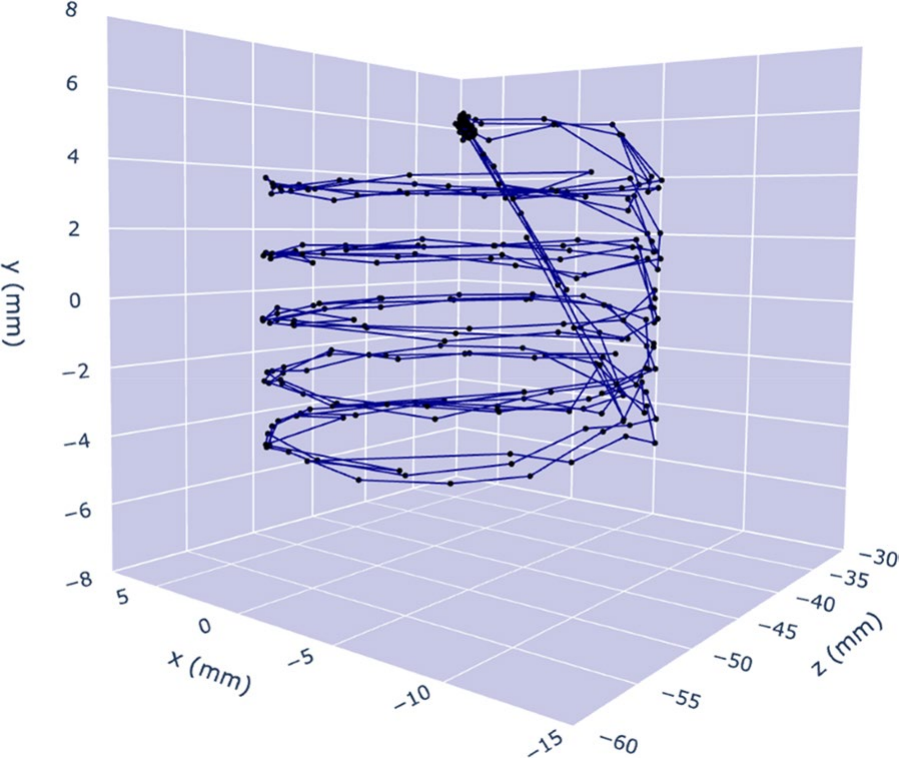
Example of tracking a low activity particle using PEPT. The printer was programmed to perform a coil pattern with a duration of 30 s; during the acquisition the printer repeated the pattern 3 times. The y-direction is in the axial direction of the scanner.

**Fig. 3:**
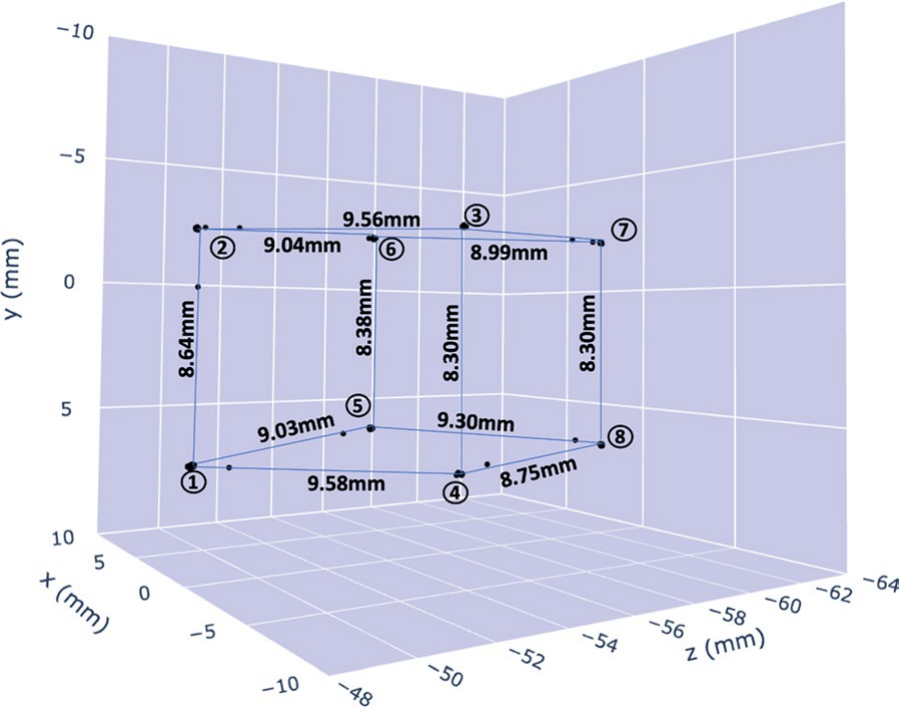
Distance estimation using PEPT. The 3D printer was programmed to hold a point source in the vertices of a 1 cm cube for 30 s in each location. Vertices labelled 1 through 8 with distance estimation shown. Black points represent calculated positions, the majority lie in the vertices, a few points in between can be visualised representing the motion of the point source as it travelled between corners.

**References**

[1] C. R. K. Window-Yule et al. Recent advances in positron emission particle tracking: a comparative review. Rep. Prog. Phys. 2022; 85: 016101.

[2] D.J. Parker, C.J. Broadbent, P. Fowles, M.R. Hawkesworth, P. McNeil. Positron emission particle tracking—a technique for studying flow within engineering equipment. Nucl. Instrum. Methods Phys. Res. A. 1993; 326:592–607.

[3] X. Fan, D.J. Parker, M.D. Smith. Labelling a single particle for positron emission particle tracking using direct activation and ion-exchange techniques. Nucl. Instrum. Methods Phys. Res. Section A. 2006; 562: 345–350.

## P9 Comparison of ultra-low-dose and standard-dose, total-body [18F]FDG-PET/CT acquisitions in healthy volunteers and lung cancer patients

### Daria Ferrara^1*^, Thomas Beyer^1^, Lalith Kumar Shiyam Sundar^1^, Werner Langsteger^2^, Rainer Bartosch^2^, Ingrid Leitinger^2^, Harald Ibeschitz^2^, Barbara Geist^2^, Kilian Kluge^2^, Josef Yu^2^, Marcus Hacker^2^, and Ivo Rausch^1^

#### ^1^Quantitative Imaging and Medical Physics (QIMP) Team, Center for Medical Physics and Biomedical Engineering, Medical University of Vienna, Vienna, Austria; ^2^Department of Biomedical Imaging and Image-Guided Therapy, Division of Nuclear Medicine, Medical University of Vienna, Vienna, Austria

##### **Correspondence:** Daria Ferrara (daria.ferrara@meduniwien.ac.at)

*EJNMMI Physics* 2023, **10(1)**:P9

**Background**

The study investigated the accuracy of ultra-low-dose (ULD) total-body (TB) PET/CT imaging for semi-/quantitative [18F]FDG-imaging of healthy volunteers and lung cancer patients.

**Materials and methods**

A Siemens Biograph Vision 600 system was used to perform dynamic (0–67 min post-injection) TB acquisitions of 20 healthy volunteers (19–62 years, 46–104 kg, 10 M/10F) and 8 lung cancer patients (47–77 years, 50–88 kg, 4 M/4F). All subjects were injected with 28 ± 2 MBq (ULD) and 279 ± 15 MBq (standard-dose, STD) [18F]FDG; the latter at 90 min post ULD injection. In all subjects, low-dose CT was used to automatically segment 12 volumes-of-interest (VOIs): adrenal glands, aorta, brain, heart, kidneys, liver, pancreas, spleen, thyroid, inferior vena cava, skeleton, lung; in cancer patients, FDG-avid lesions were manually delineated on static images (55–67 min post-injection) and standardized-uptake-values (SUVs) were extracted. Patlak parameters were derived from the last 15-min dynamic data acquisition with the vendor’s automatic TB Patlak reconstruction. Parameters were compared for both ULD/STD conditions and tested with paired t-tests.

**Results**

ULD/STD SUVs were similar (mean %-difference < 5%) for healthy volunteers and lung cancer patients in all VOIs (*p* > 0.05), except heart, brain, and liver. Dynamic imaging resulted in significant differences between ULD/STD Patlak parameters in 50% of the VOIs (mean %-difference > 30%). 28 tumours were delineated on STD images, while only 12 were found on ULD images, due to increased image noise. Lesion SUVs were similar in ULD and STD (*p* = 0.17, mean %-difference < 10%).

**Conclusions**

ULD and STD [18F]FDG injections in healthy volunteers and lung cancer patients provided comparable SUVs, except in organs with fast glucose turnover. Patient dose exposure can be reduced in TB screening simple settings using [18F]FDG-PET/CT without a loss in SUV accuracy. However, Patlak parameters were generally not comparable between ULD/STD injections and ULD scans did not allow precise tumour delineations.

**Ethics approval**

All data utilised in this study were acquired in accordance with the Declaration of Helsinki. Written information consent was obtained from all the subjects prior to examinations. Reference number: EK1907/2020.

## P10 Comparison between aorta and carotid artery image derived input functions for kinetic analysis of cerebral glucose consumption

### Laura Providência*, Chris W. J. van der Weijden, Philipp Mohr, Rudi A. J. O. Dierckx, Adriaan A. Lammertsma, Charalampos Tsoumpas.

#### Medical Imaging Center, University Medical Center Groningen, Groningen, Netherlands

##### **Correspondence:** Laura Providência (l.lopes.goncalves.da.providencia@umcg.nl)

*EJNMMI Physics* 2023, **10(1)**:P10

**Background**

Conventional PET scanners, with limited axial field-of-view (FOV), make the internal carotid arteries a popular region for IDIF extraction in quantitative PET brain studies. However, time activity curves (TACs) extracted from the internal carotids (diameter 3.9–6.0 mm) are prone to partial volume effects (PVE) due to relatively low PET resolution (~ 4 mm). Long axial FOV (LAFOV) scanners enable using the aorta (diameter 25–38 mm) to extract the IDIF [1,2]. This study assessed whether an IDIF obtained from the carotids could be used to derive cerebral glucose consumption when the aorta is not within the FOV.

**Materials and methods**

Seven patients underwent a 65 min [^18^F]FDG LAFOV-PET/CT scan. IDIFs were obtained by placing VOIs in the internal carotids (4 highest intensity pixels) and ascending aorta. Kinetic modelling with both IDIFs was performed by extracting the TAC from the whole brain and using an irreversible two-tissue compartment model with additional blood-volume parameter (2T3k_V_B_) and Patlak analysis (t* = 40 min) to derive the model rate constants (*K*_*1*_, *k*_*2*_, *k*_*3*_), net tracer flux (*K*_*i*_), and the Patlak intercept (*Y*_0_). TACs and model parameters were analysed using a Kolmogorov–Smirnov test and a paired t-test, respectively.

**Results**

Significant differences were found between aorta and carotid TACs (p = 0.008). Visual inspection showed that TACs from the carotids underestimated the blood peaks substantially (Fig. 1) when compared with the aorta. The ratio between both TACs showed that this difference was not due to a constant scaling factor (Fig. 2). In addition, significant differences were found for *K*_*1*_, *K*_*i*_ (2T3k_V_B_) and *Y*_0_ (Table 1).

**Conclusions**

An IDIF extracted from the carotids suffers from PVE and leads to significantly biased model parameters and glucose consumption estimates compared with those obtained using an IDIF extracted from the aorta. Future studies need to assess whether PVE correction methods results in more reliable IDIFs from the carotids.

**Fig. 1:**
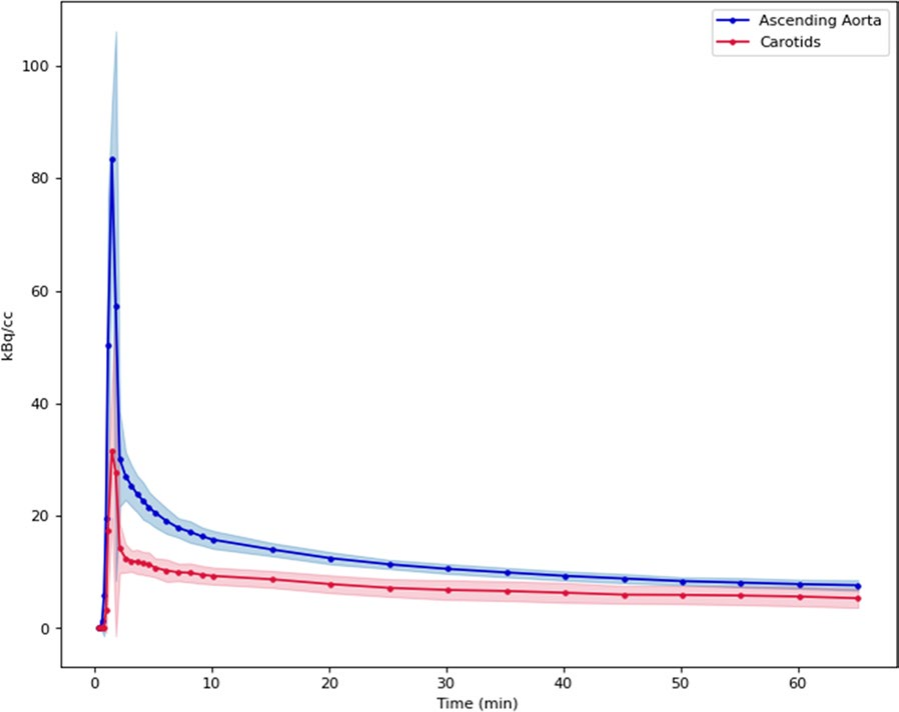
Time activity curves extracted from the VOIs placed in aorta and carotids.

**Fig. 2:**
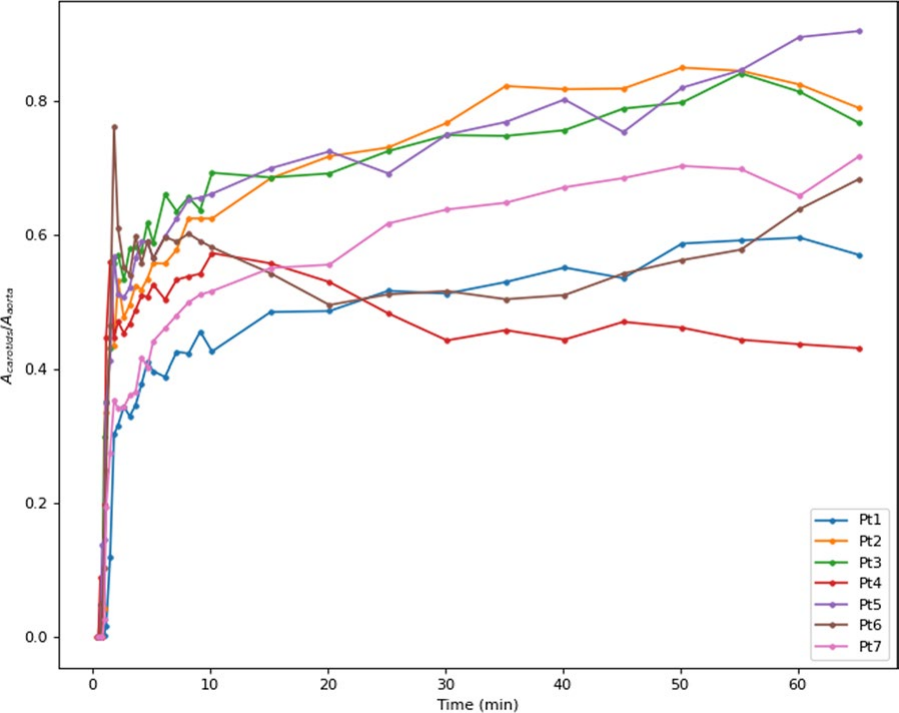
Ratio between aorta and carotids TAC.

**Table 1:** Parameters obtained with 2T3k model and Patlak analysis.

		Aorta	Carotids	*p* value
2T3k_VB	K_1_ (mL∙cm^−3^∙min^−1^)	0.084 ± 0.009	0.204 ± 0.052	< 0.001***
	k_2_ (min^−1^)	0.202 ± 0.027	0.235 ± 0.039	0.10
	k_3_ (min^−1^)	0.076 ± 0.021	0.055 ± 0.010	0.05
	K_i_ (mL∙cm^−3^∙min^−1^)	0.023 ± 0.004	0.039 ± 0.010	0.003**
Patlak	K_i_ (mL∙cm^−3^∙min^−1^)	0.021 ± 0.004	0.028 ± 0.011	0.10
	Y_0_ (mL∙cm^−3^)	0.322 ± 0.055	0.980 ± 0.430	< 0.006**

**References**

1. van der Weerdt A, Klein L, Boellaard R, Visser C, Visser F, Lammertsma A. Image-derived input functions for determination of MRGlu in cardiac ^18^F-FDG PET scans. Journal of Nuclear Medicine. 2001; 42(11): 1622–9.

2. Henriksen A, Lonsdale M, Fuglø D, Kondziella D, Nersesjan V, Marner L. Non-invasive quantification of cerebral glucose metabolism using Gjedde-Patlak plot and image-derived input function from the aorta. NeuroImage. 2022; 253: 119079.

## P11 A fully-automated, tracer independent, diffeomorphic framework for total-body PET/CT normative database generation for system-level assessment of human physiology

### Sebastian Gutschmayer^1*^, Otto Muzik^2^, Marcus Hacker^3^, Daria Ferrara^1^, Sven Zuehlsdorff^4^, Hartwig Newiger^5^, Thomas Beyer^1^ and Lalith Kumar Shiyam Sundar^1^

#### ^1^Quantitative Imaging and Medical Physics (QIMP) Team, Center for Medical Physics and Biomedical Engineering, Medical University of Vienna, Vienna, Austria; ^2^Department of Pediatrics, Wayne State University School of Medicine, Children’s Hospital of Michigan, Detroit, MI, USA; ^3^Department of Biomedical Imaging and Image-Guided Therapy, Division of Nuclear Medicine, Medical University of Vienna, Vienna, Austria; ^4^Siemens Medical Solutions USA, Inc., Hoffman Estates, IL, USA; ^5^Siemens Healthcare GmbH, Erlangen, Germany

##### **Correspondence:** Sebastian Gutschmayer (sebastian.gutschmayer@meduniwien.ac.at)

*EJNMMI Physics* 2023, **10(1)**:P11

**Background**

A fully-automated methodology to establish a preliminary voxel-based normative database (NormDB) using total-body [18F]FDG-PET/CT images is presented. This method is based on CT images and, thus, tracer independent while enabling voxel-wise comparisons of tracer accumulation in selected patients versus the NormDB.

**Materials and methods**

In this ongoing study, 20 whole-body, scatter- and attenuation-corrected PET/CT images from healthy volunteers were used to build a preliminary NormDB. All subjects were stratified based on sex and BMI. A template image for each stratified group is initially required to generate a group-specific NormDB and established by an iterative process. Therefore, an AI-based, automated multi-organ segmentation tool segmented multiple tissue compartments (13 organs, skeleton, fat, muscle) from all subjects. The segmentations of all subjects within a group were then diffeomorphically registered to a randomly chosen reference subject's segmentations from the group. Based on the deformation fields, corresponding CT images were aligned and summed to a mean template image. This process was repeated with the newly created template image as a reference for the next iteration or stopped when no increase in sharpness in the template image was detectable. The template image was used as a reference for registering the subject CT images; corresponding deformation fields were applied to the related PET images. Averaging them results in a cohort-specific total-body normative PET image. Dice score and average symmetric surface distance (ASSD) between the segmentations were used as quality metrics for spatial-normalisation accuracy.

**Results**

The average Dice score and ASSD between the subjects and the template image were 0.8 ± 0.1 and 106 ± 168 mm, respectively.

**Conclusions**

The evaluated metrics show an improved alignment of subject images during template creation and between subjects and the template image. Voxel-wise normative values could be compared to a patient image in future steps to generate patient-specific z-maps.

**Ethics approval**

All data utilised in this study were acquired in accordance with the Declaration of Helsinki. Written information consent was obtained from all the subjects prior to examinations. Reference number: EK1907/2020.

## P12 Development of In-house Stringent Protocols and Phantoms for Quality Assurance of a Clinical and Research Total-Body PET Scanner

### Benjamin A. Spencer^1,2^*, Negar Omidvari^2^, Eric Berg^2^, Enette M. Revilla^2^, Reimund Bayerlein^1^, Yasser G. Abdelhafez^1^, George Burkett^1^, Jeffrey P. Schmall^1,3^, Mike Nguyen^1^, Zhaoheng Xie^2^, Elizabeth Li^2^, Edwin K. Leung^1,2,3^, Aaron Selfridge^2^, Yansong Zhu^1^, Yiran Wang^1,2^, Heather Hunt^1^, Kristin McBride^1^, Lynda Painting^1^, Ofilio Vigil^1^, Denise T. Caudle^1^, Emilie Roncali^1,2^ Terry Jones^1^, Lorenzo Nardo^1^, Simon R. Cherry^2,1^, Ramsey D. Badawi^1,2^

#### ^1^Department of Radiology ^2^Department of Biomedical Engineering, University of California Davis, CA, USA,^3^UIH America Inc., Houston, TX, USA

##### **Correspondence:** Benjamin A. Spencer (benspencer@ucdavis.edu)

*EJNMMI Physics* 2023, **10(1)**:P12

**Background**

With their widespread adoption in routine clinical care and a variety of research applications, total-body PET scanners will play a key role in the future of PET research. This work presents the methodology for a stringent and robust quality assurance program implemented on the world’s first total-body PET scanner at UC Davis [1], including results from phantom and clinical quality assurance methods spanning three years of implementation.

**Methods and results**

A. Daily count-rate monitoring: Average LYSO background singles-rate provides a fast and efficient daily check of the system performance (Fig. 1). B. Quantitative accuracy: Weekly scans of a ^68^Ge uniform cylinder in three axial locations can ensure quantitative accuracy is consistent axially and temporally [2] (Fig. 2A). C. Cross-calibration: Semi-annual scans of a uniform ^18^F-filled phantom will ensure the scanner is accurately cross-calibrated with the dose calibrator (Fig. 2B). This should be done following calibrations or more frequently if needed. D. Image quality investigation: A quantitative investigation of the entire axial field-of-view (AFOV) is performed annually, using multiple NEMA image quality phantoms spanning the AFOV scanned at several activity levels representative of clinical and research protocols in use (following [3]). E. Scatter correction evaluation: An in-house developed large fillable phantom, including a removable fat tissue-equivalent material compartment, is scanned after software updates to ensure robustness of data corrections. F. Long-term temporal monitoring of system-wide performance with real patient data: Total-body scanners uniquely allow utilizing every human subject scan for quality assurance by calculating the ratio of the total human count-rate obtained from each subject scan to the known activity at scan time as an estimate of the temporal stability of the scanner (Fig. 3).

**Conclusions**

These quality assurance protocols ensure thorough and stringent examination of total-body PET scanner performance and stability to facilitate clinical care and research reliability.

**Fig. 1:**
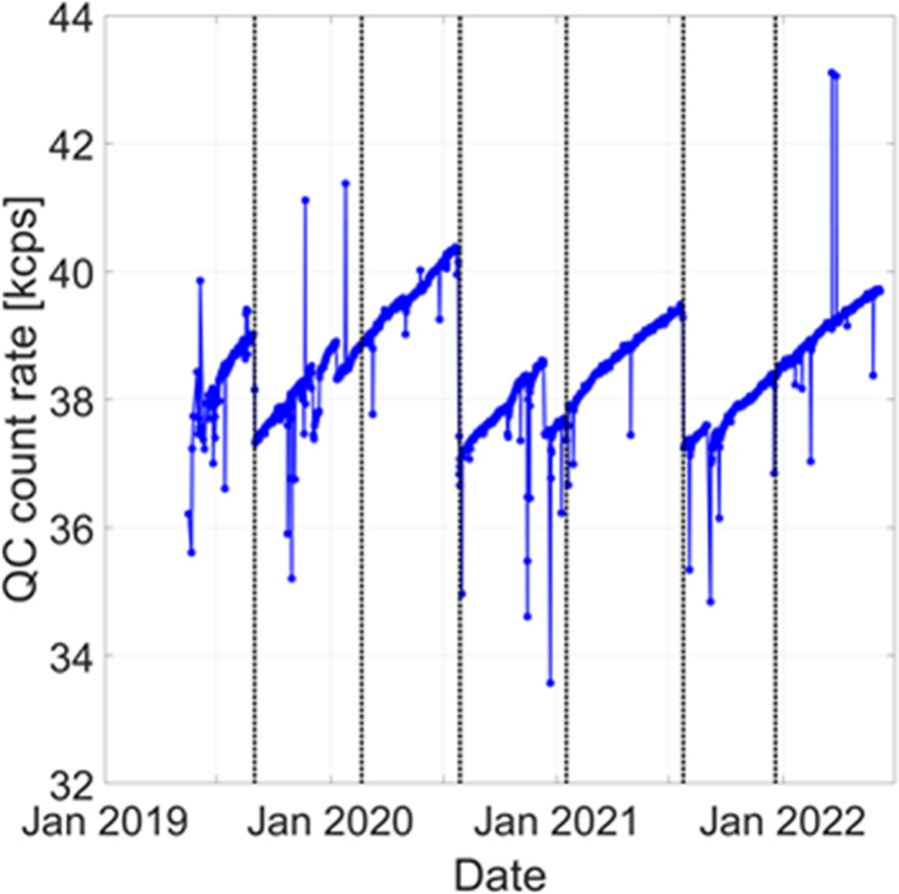
Daily LYSO background count-rate measured over three years, where dotted vertical lines indicate dates of calibration. Background count-rates increase over time due to energy histogram drift and then shift back to baseline after energy calibration. Several anomalous high or low count-rate occurrences are measured, possibly due to detector temperature changes or count-rate measurement in presence of a radioactive source.

**Fig. 2:**
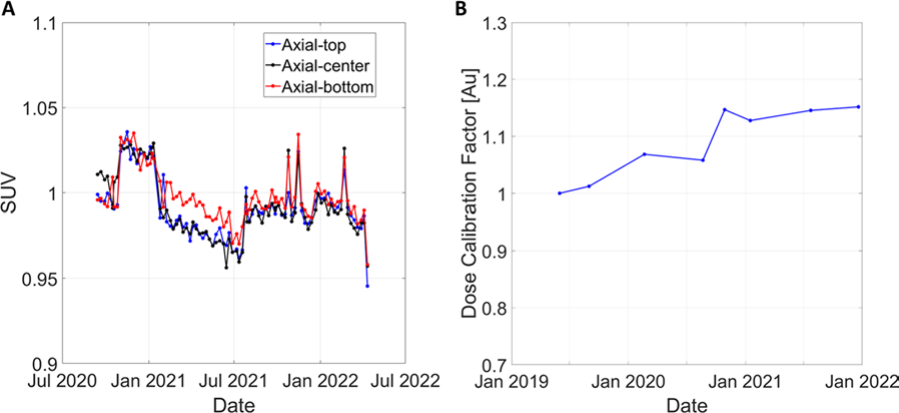
(A) Weekly ^68^Ge uniform cylinder measurement over three years showing quantitative stability within 3% axially and within 5% temporally. (B) Dose calibration factor measured over three years. Changes in dose calibration factor and weekly quantitative accuracy may be due to reconstruction software and data correction updates.

**Fig. 3:**
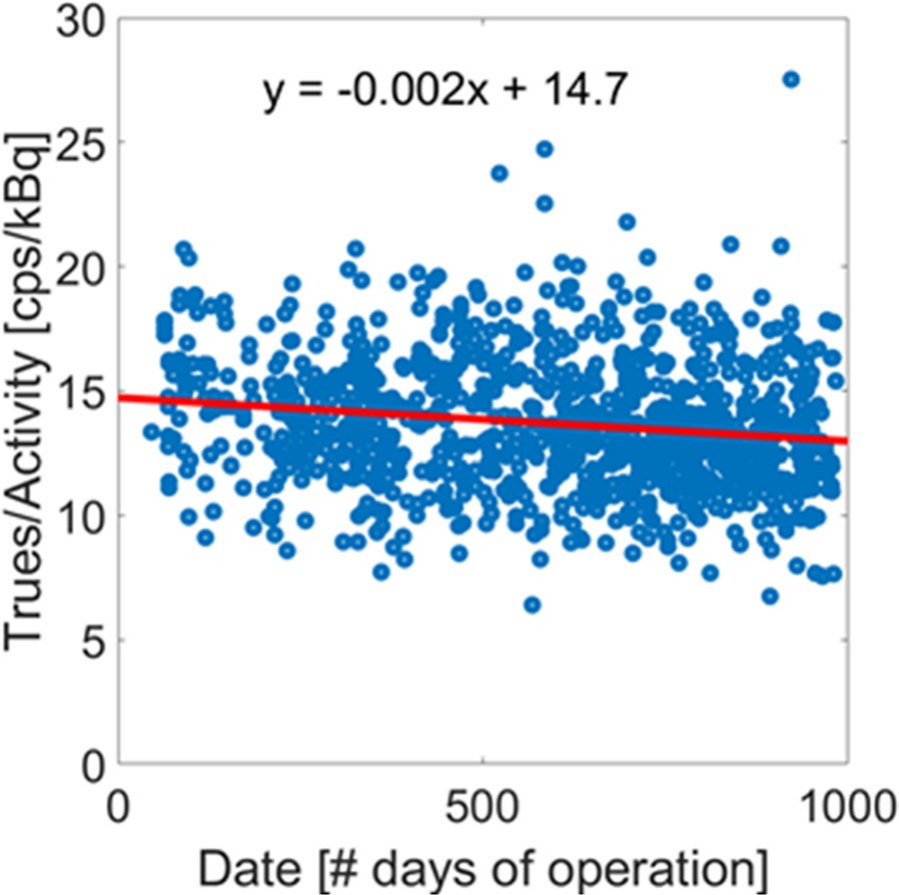
Ratio of trues count-rate over the injected dose at scan time from 942 clinical 18F-FDG exams during a 900-day period. The spread in the data is likely due to physiologic factors such as the size of the patient (attenuation/scatter fraction) and the radioactivity amount voided by the patient. Overall, the close-to-flat trend indicates good scanner performance stability over time.

**Ethics approval**

The study was approved by the IRB of University of California, Davis.

**References**

1. Spencer BA, Berg E, Schmall JP, et al. Performance evaluation of the uEXPLORER Total-body PET/CT scanner based on NEMA NU 2-2018 with additional tests to characterize long axial field-of-view PET scanners. J Nucl Med., 2021;62:861–870.

2. Nguyen M, McBride K, Hunt H, et al. Temporal and Axial Quantitative Uniformity Measurements of Total-body PET Systems. J Nucl Med. 2021;62 (suppl 1):3041.

3. Leung EK, Berg E, Omidvari N, et al. Quantitative accuracy in total-body imaging using the uEXPLORER PET/CT scanner. Phys Med Biol. 2021;66(20).

## P13 Comparative studies of the selected issues of sparse and full geometries of total-body PET scanners

### Szymon Parzych^1,2,3*^ on behalf of the J-PET Collaboration

#### ^1^Faculty of Physics, Astronomy, and Applied Computer Science, Jagiellonian University, Łojasiewicza 11, 30-348 Kraków, Poland; ^2^Total Body Jagiellonian-PET Laboratory, Jagiellonian University, Kraków, Poland; ^3^Center for Theranostics, Jagiellonian University, Poland

##### **Correspondence:** Szymon Parzych (szymon.parzych@doctoral.uj.edu.pl)

*EJNMMI Physics* 2023, **10(1):**P13

**Background**

Despite of one of the main benefits of the Total-Body PET tomographs—their greatly enhanced sensitivity over the significantly extended field of view, their widespread use is being held back due to the construction expenses. The aim of this study is to inspect and compare simulation-based sensitivity of existing and presently developed scanners, while also focusing on their costs and scintillators characteristics.

**Materials and methods**

For the case of this study, J-PET systems with different geometrical configurations, axial lengths, ring systems and scintillator cross-sections were studied. Moreover, PET scanners based on the uEXPLORER geometrical design were inspected, varying in parameters such as: utilized scintillator material (BGO, LYSO), axial length and detector configuration (full and sparse). In addition, the Biograph Vision Quadra was also investigated. All of the simulations were conducted with the GATE software. Furthermore, the examination of the angle- and ring-based acceptance criterion was performed due to its utilization in oblique LOR elimination. Estimation of construction costs was done by taking into account total photomultiplier-covered surface and different scintillator materials.

**Results**

The best sensitivity-wise performance can be achieved with the BGO crystals, however performance of this material is a drawback to its exploitation. The utilization of the sparse geometrical configuration has a direct impact on the construction costs, however it highly degrades the system's sensitivity over the discontinuous field of view. The atypical geometrical design provided with plastic scintillators in J-PET scanners gives an alternative approach to lower the expenses, while maintaining an almost continuous field of view. Nevertheless, it suffers from the relatively low efficiency performance.

**Conclusions**

The presented simulation-based study took into consideration advantages (in a form of sensitivity) and drawbacks (in a form of costs and materials performance) of the existing and presently developed Total-Body PET scanners in the issue of their widespread usage.

**Acknowledgements**

The abovementioned work is presented on behalf of the J-PET Collaboration. This work was supported by Foundation for Polish Science through TEAM POIR.04.04.00-00-4204/17, the National Science Centre, Poland (NCN) through grant No. 2021/42/A/ST2/00423, PRELUDIUM 19, agreement No. UMO-2020/37/N/NZ7/04106 and the Ministry of Education and Science under the grant No. SPUB/SP/530054/2022. The publication has been also supported by a grant from the SciMat and qLife Priority Research Areas under the Strategic Programme Excellence Initiative at the Jagiellonian University. The work has been also supported by the Jagiellonian University via project CRP/0641.221.2020, and via grant from the SciMat and qLife Priority Research Areas under the Strategic Programme Excellence Initiative at the Jagiellonian University. The study was funded by “Laboratories of the Youth” as part of the “Excellence Initiative—Research University” program at the Jagiellonian University in Kraków.

## P14 Y-90 total-body PET system modeling with Monte Carlo simulations

### Gustavo Costa^1^*, Negar Omidvari^1^, Benjamin Spencer^1^, Emilie Roncali^1,2^

#### ^1^Department of biomedical engineering, University of California-Davis, Davis, CA 95616 USA; ^2^Department of radiology, University of California-Davis, Davis, CA 95616 USA

##### **Correspondence:** Gustavo Costa (gccosta@ucdavis.edu)

*EJNMMI Physics* 2023, **10(1):**P14

**Background**

Yttrium-90 (Y-90) PET images are inherently noisy due to the extremely low positron emission rate of Y-90 but other factors such as dead-time can also contribute to the high noise especially with the high bremsstrahlung emission rate from the **β**^−^ emission that occurs with therapeutic activities, much higher than those with typical diagnostic imaging.

**Materials and methods**

Monte Carlo simulations of a total-body PET scanner were performed to investigate the effect of system saturation on Y-90 PET imaging by introducing dead-time into the PET detector response model. Line sources were modeled: (1) the Y-90 full emission spectrum (2.5 GBq), (2) the Y-90 with no bremsstrahlung (2.5 GBq), and (3) an equivalent number of emissions with mono-energetic back-to-back 511-keV gammas (76 kBq). Dead-time values from 0 to 500 ns were simulated in 0.5 s time-frames and the number of singles and coincidences within an energy window of 430–645 keV were evaluated with no reconstruction (data collected from list-mode equivalent output from GATE).

**Results**

The introduction of the detector dead-time decreased both the number of singles and coincidences detected throughout all simulation sets. As expected, the 76 kBq and no-bremsstrahlung simulations show a small decrease of singles and coincidences (Fig. 1) reaching a plateau at 10 ns suggesting the stabilization of the system. With the addition of the bremsstrahlung emission, a steady decrease is observed becoming much faster from 100 ns. Interestingly, the number of coincidences with no-bremsstrahlung is approximately 5% of those with bremsstrahlung suggesting that the larger number of random coincidences may also contribute to image degradation.

**Conclusions**

System saturation due to dead-time and high number of randoms can degrade images. However, with clear understanding and quantification of the effects of these factors, calibration and reconstruction can be tuned for Y-90 imaging to improve the image quality.

**Fig. 1:**
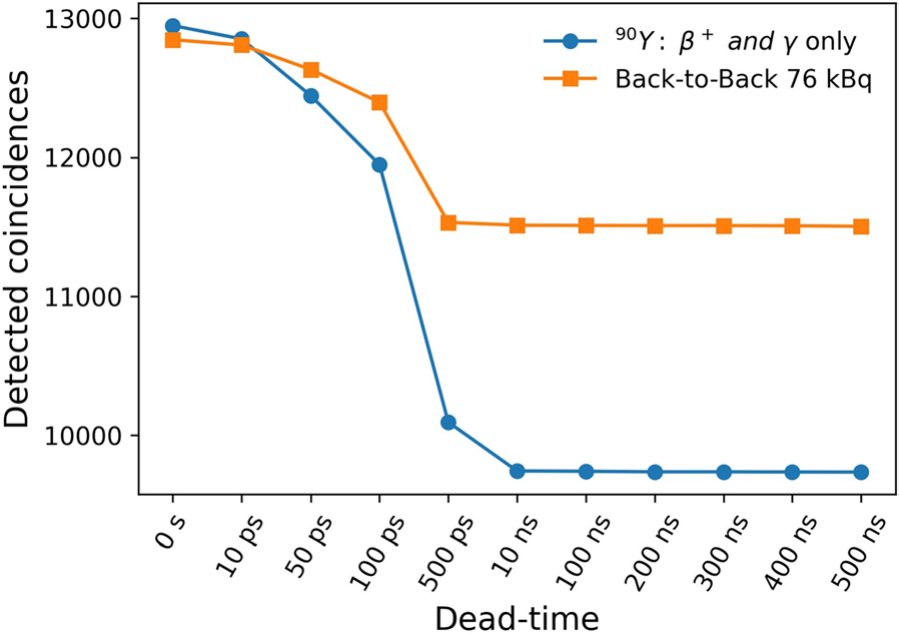
Number of detected coincidences for simulations of Y-90 with no bremsstrahlung (positron and gamma only) compared to simulations of mono-energetic (511 keV) back-to-back gammas for all tested dead-times.

## P15 Monte Carlo simulations of the Siemens Biograph Vision Quadra

### Christian M Pommranz^1,2*^, Jorge Cabello^3^, Julia G Mannheim^1,4^, Andrea Santangelo^2^, Christian la Fougère^5^, Bernd J Pichler^1,4^, Fabian P Schmidt^1,5^

#### ^1^Werner Siemens Imaging Center, Department of Preclinical Imaging and Radiopharmacy, Eberhard Karls University Tuebingen, Roentgenweg 13, 72,076 Tuebingen, Germany; ^2^Institute for Astronomy and Astrophysics, Eberhard Karls University Tuebingen, Sand 1, 72,076 Tuebingen, Germany; ^3^Siemens Medical Solutions USA, Inc., Knoxville, TN, USA; ^4^Cluster of Excellence iFIT (EXC 2180) “Image Guided and Functionally Instructed Tumor Therapies”, University of Tuebingen, Tuebingen, Germany; ^5^Department of Nuclear Medicine and Clinical Molecular Imaging, University hospital Tuebingen, Tuebingen, Germany

##### **Correspondence:** Christian M Pommranz (pommranz@astro.uni-tuebingen.de)

*EJNMMI Physics* 2023, **10(1):**P15

For Monte Carlo simulations of the Siemens Biograph Vision Quadra total-body PET/CT scanner two digitization and analysis workflows (Fig. 1) are currently developed and investigated using the ‘**G**eant4 **A**pplication for **E**mission **T**omography’ (GATE) [1, 2].

Workflow 1 starts with the GATE single event output provided in ROOT data format and uses a coincidence sorter and analysis tool provided by Siemens to produce coincidences in a list-mode data format, that can be used for image reconstruction with the Siemens e7tools. This workflow is designed to simulate the PET scanner with high accuracy by incorporating device-specific analysis and by implementing the same image reconstruction (PSF-TOF 4i5s) that is used in clinical routine.

Workflow 2 uses the GATE coincidence sorter output, which is translated into a ‘**C**ustomizable and **A**dvanced **S**oftware for **To**mographic **R**econstruction’ (CASToR) [3] readable list-mode data format. Image reconstruction is then performed by CASToR using a look-up table containing precise positions and orientations of the individual scintillation crystals. While Workflow 2 is limited in accuracy by using more generic modelling of digitization and coincidence sorting as well as a different image reconstruction toolkit, it offers increased flexibility for adaptations along the entire workflow. For example, Workflow 2 enables to investigate the effects of modifications of the scanner geometry or to include a PET insert with the option to record coincidences between insert and total-body scanner.

Reconstructed non attenuation corrected images of a 10 s simulation of a NEMA image quality phantom with a total activity of 60.9 MBq using Workflow 1 and Workflow 2 are shown in Fig. 2. Future work will include optimization of Workflow 2 and comparison of simulated NEMA PET performance characteristics to measurements.

**Fig. 1:**
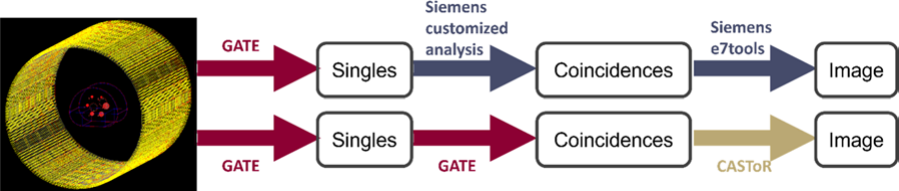
Simulation model of the Biograph Vision Quadra and digitization and analysis workflows: Workflow 1 (upper) and Workflow 2 (lower).

**Fig. 2:**
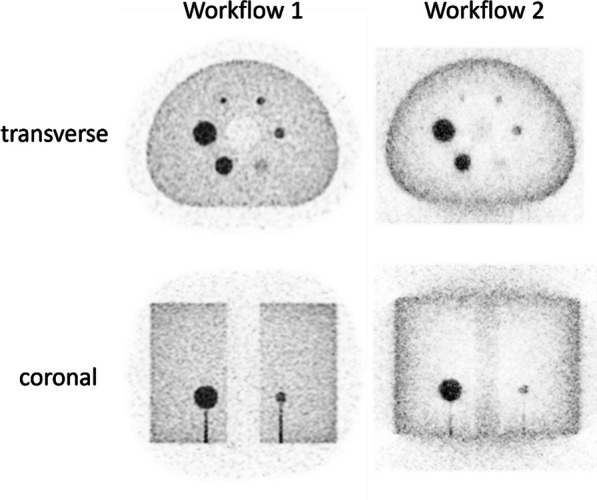
Reconstructed image of the NEMA image quality phantom using the Siemens e7tools (Workflow 1) and using CASToR (Workflow 2).

**References**

1. Jan S, Santin G, Strul D, Staelens S, Assié K, Autret D, et al. GATE: a simulation toolkit for PET and SPECT. Phys Med Biol. 2004;49:4543–61.

2. Jan S, Benoit D, Becheva E, Carlier T, Cassol F, Descourt P, et al. GATE V6: a major enhancement of the GATE simulation platform enabling modelling of CT and radiotherapy. Phys Med Biol. 2011;56:881–901.

3. Merlin T, Stute S, Benoit D, Bert J, Carlier T, Comtat C, et al. CASToR: a generic data organization and processing code framework for multi-modal and multi-dimensional tomographic reconstruction. Phys Med Biol. 2018;63:185005.

## P16 A simulation study to evaluate NEMA characteristics in Modular J-PET

### Faranak Tayefi Ardebili^1,2,3^*, Syzmon Niedzwicki^1,2,3^, Pawel Moskal^1,2,3^

#### ^1^Faculty of Physics, Astronomy, and Applied Computer Science, Jagiellonian University, Łojasiewicza11, 30-348 Kraków, Poland; ^2^Total Body Jagiellonian-PET Laboratory, Jagiellonian University, Kraków, Poland; ^3^Center for Theranostics, Jagiellonian University, Poland

##### **Correspondence:** Faranak Tayefi Ardebili (faranaktayefi.tayefi.ardebili@doctoral.uj.edu.pl)

*EJNMMI Physics* 2023, **10(1):**P16

**Background**

The promising imaging performance of Positron Emission Tomography (PET) made it a suitable imaging modality for malignant lesion localization in oncology studies, drug delivery, physiological studies, etc. [1]. There is ongoing research on a new generation of PET tomographs done by J-PET Collaboration, which would be competitive to other clinically available systems [2].

The currently investigated prototype of Jagiellonian PET is a Modular J-PET [3]. Its structure consists of 24 detection panels, that provide in a total of 50 cm of the AFOV. The modularity of prototype allows for simple construction and deconstruction, portability, and the possibility of assembly with different numbers of panels depending on the patient size and clinical needs [4].

The main aim of this simulation study is to estimate the performance of the Modular J-PET according to the NEMA NU 2-2018 standards.

**Methods**

The presented study was carried out by the GATE software which is a validated simulation toolkit for a medical imaging application 5]. This software is used to simulate recommended procedures by NEMA NU 2-2018 to estimate characteristics such as spatial resolution, sensitivity, scatter fraction, NECR and image quality of Modular J-PET.

**Results**

Achieved results provide appropriate estimation from the operation of the Modular J-PET by considering the different functional aspects of the system. Estimated NEMA parameters will be compared with current clinical tomographs.

**Conclusion**

Performance characteristics of Modular J-PET based on the plastic scintillator were determined according to the NEMA NU 2-2018 by GATE simulation software.

**Acknowledgements**

This work was supported by Foundation for Polish Science through TEAM POIR.04.04.00-00-4204/17, the National Science Centre, Poland (NCN) through grant No. 2021/42/A/ST2/00423, PRELUDIUM 19, agreement No. UMO-2020/37/N/NZ7/04106 and the Ministry of Education and Science under the grant No. SPUB/SP/530054/2022. The publication has been also supported by a grant from the SciMat and qLife Priority Research Areas under the Strategic Programme Excellence Initiative at the Jagiellonian University. The work has been also supported by the Jagiellonian University via project CRP/0641.221.2020, and via grant from the SciMat and qLife Priority Research Areas under the Strategic Programme Excellence Initiative at the Jagiellonian University. This study is on behalf of the J-PET collaboration.

**References**

1. Moskal P, et al. Simulating NEMA characteristics of the modular total-body J-PET scanner—an economic total-body PET from plastic scintillators. Phys. Med. Biol. 2021. 66: 175015.

2. Moskal P, et al. Positronium imaging with the novel multiphoton PET scanner. Science Advances.2021. 7: 4394.

3. Dadgar M, Kowalski P. Gate simulation study of the 24-module J-PET scanner: data analysis and image reconstruction. Acta Physica Polonica B. 2020. 51:309–311.

4. Moskal P, et al. Positronium in medicine and biology. Nature Reviews Physics. 2019. 9: 527–529.

5. Jan S, et al. GATE V6: a major enhancement of the GATE simulation platform enabling modelling of CT and radiotherapy. Phys. Med. Biol. 2011. 56: 881–901.

## P17 A simulation study to investigate the sensitivity of total-body J-PET for various lengths of the plastic scintillator

### Keyvan Tayefi Ardebili^1,2,3^*, Syzmon Niedzwicki^1,2,3^, Pawel Moskal^1,2,3^

#### ^1^Faculty of Physics, Astronomy, and Applied Computer Science, Jagiellonian University, Łojasiewicza11, 30-348 Kraków, Poland; ^2^Total Body Jagiellonian-PET Laboratory, Jagiellonian University, Kraków, Poland; ^3^Center for Theranostics, Jagiellonian University, Poland

##### **Correspondence:** Keyvan Tayefi Ardebili (keyvan.tayefiardebili@doctoral.uj.edu.pl)

*EJNMMI Physics* 2023, **10(1):**P17

**Background**

Total-Body PET scanner due to the larger detection area, provides higher sensitivity which plays a key role in the overall performance of the tomographs. A new generation of Total-Body PET scanners based on plastic scintillators is being developed by J-PET collaboration. One of the approaches in the development of the Total-Body J-PET is the ring wise configuration [1, 2]. The main aim of this study is to investigate the sensitivity of Total-Body J-PET for different numbers of rings with the specific axial field of views (AFOVs).

**Methods**

Total body J-PET scanner comprises of 7 rings, each ring consisting of 24 modules. A single module consists of 32 scintillators of 33 cm length divided into two layers and with additional layer of 50 WLS fibers. For the case of this study, the proposed Total-Body J-PET tomograph is simulated by GATE software (Fig. 1). To evaluate the sensitivity of the aforesaid scanner, the simulations according to the procedure recommended by NEMA-NU-2-2018 is performed [3,4]. The sensitivity of the scanner is investigated with the 70 cm line source for 1 ring and 261.7 cm line source for 7 rings that are located in the center of the scanner and I used the same activity for both of them to calculate the sensitivity.

**Results**

The results of this study would be to perform a comparison sensitivity between 1 and 7 rings tomographs based on the J-PET technology.

**Conclusion**

The investigation of the sensitivity of Total-Body J-PET in various numbers of the detector rings provide the possibility of optimum event selection for image reconstruction.

**Fig. 1:**
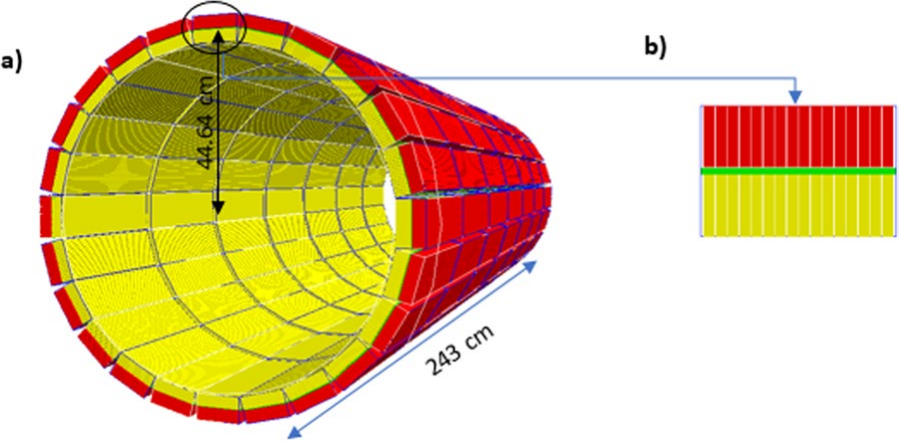
**a** TB-J-PET for 7 rings with AFOV = 243 cm simulated by GATE software. **b** Two scintillator layers (yellow and red color), and the WLS layer (green color) are shown in the zoomed view.

**Acknowledgements**

The abovementioned work is presented on behalf of the J-PET Collaboration.

**References**

1. Moskal P, et al. Simulating NEMA characteristics of the modular total-body J-PET scanner—an economic total-body PET from plastic scintillators. Phys. Med. Biol. 2021. 66: 175015.

2. Niedźwiecki Sz, et al. J-PET: A New Technology for the Whole-body PET Imaging. Acta Phys. Polon. 2017. B48 10: 1567–1576.

3. Kowalski P, et al. Estimating the NEMA characteristics of the J-PET tomograph using the GATE package. Phys. Med. Biol. 2018. 63: 165008.

4. Benjamin A. Spencer, et al. Performance evaluation of the uEXPLORER Total-body PET/CT scanner based on NEMA NU 2-2018 with additional tests to characterize long axial field-of-view PET scanners. Journal of Nuclear Medicine. 2020.

## P18 Full FOV 2 mm monolithic Total Body PET—system simulation based on experimental detector data

### Maya Abi Akl^1,2^*, Othmane Bouhali^2^, Meysam Dadgar^3^, Stefaan Vandenberghe.^1^

#### ^1^Medical Image and Signal Processing, Ghent University, Ghent, Belgium; ^2^Science Program, Texas A&M University at Qatar; ^3^Faculty of Physics, Astronomy and Applied Computer Science, Jagiellonian University, 30–348 Cracow, Poland.

##### **Correspondence:** Maya Abi Akl (maya.abi_akl@qatar.tamu.edu)

*EJNMMI Physics* 2023, **10(1):**P18

**Background**

Total Body PET (TB-PET) systems recently introduced in research and clinical departments provide a multifold increase in sensitivity compared to the standard axial field of view (FOV) PET scanners [1–4]. With spatial resolution limited by crystal pixel width affecting detectability and quantification accuracy, monolithic detectors have proven superior spatial resolution with depth of interaction (DOI) [5]. Our latest measurements show that an intrinsic resolution below 1.3 mm and 6-layer DOI is accomplished [6]. In this work, we simulate monolithic LYSO long axial FOV PET designs and evaluate the system spatial resolution of the NEMA point sources.

**Materials and methods**

The simulation study includes two designs of axial lengths, 36.2 cm and 72.6 cm consisting of 7 and 14 rings respectively, each having 40 detector modules of LYSO 50 × 50 × 16 mm^3^ in size. GATE was used for the NEMA simulation of spatial resolution. Six ^18^F point-like sources were placed at three radial and two axial positions. Post-simulation processing was performed to blur the endpoints based on the distributions obtained from the intrinsic resolution of the monolithic LYSO measurements, while QETIR was used for iterative reconstruction.

**Results**

A flat spatial resolution across the transverse FOV at the central slice with a FWHM below 2 mm (Fig. 1) was obtained for the 36.2 cm long design. The axial resolution was uniform at different radial offsets.

**Conclusions**

Compared to the human PET with the highest resolution (uExplorer, 3 mm in center, 5.6 mm at 20 cm) we obtain 50% improvement at the center of the axial/transverse FOV. A 2–2.5 improvement in system resolution at off-center points is shown as this is the first whole body system with multi-layer DOI resulting in a uniform resolution over the full FOV. This will make quantification accuracy independent of the position in the patient.

**Fig. 1:**
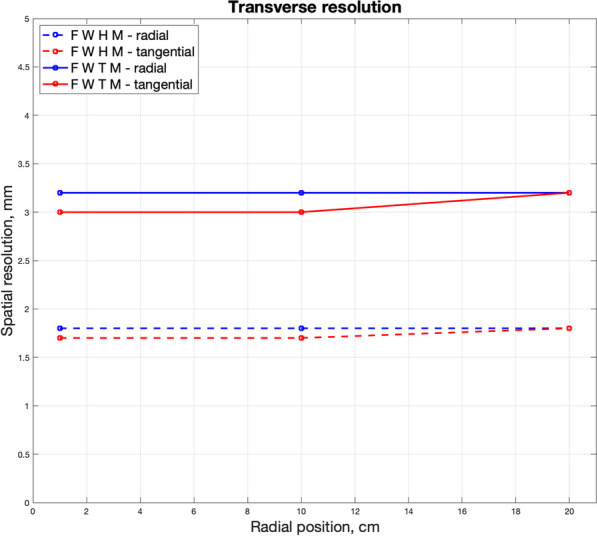
Transverse spatial resolution in the center of the axial FOV at different radial positions.

**References**

1. Vandenberghe S, Moskal P, Karp JS. State of the art in total body PET. EJNMMI Phys. 2020; 7(1):35.

2. Spencer BA, Berg E, Schmall JP, Omidvari N, Leung EK, Abdelhafez YG, Tang S, Deng Z, Dong Y, Lv Y, Bao J, Liu W, Li H, Jones T, Badawi RD, Cherry SR. Performance evaluation of the uEXPLORER Total-body PET/CT scanner based on NEMA NU 2-2018 with additional tests to characterize long axial field-of-view PET scanners with a long axial field-of-view. J Nucl Med. 2021; 62:861–70.

3. Karp JS, Viswanath V, Geagan MJ, Muehllehner G, Pantel AR, Parma MJ, Perkins AE, Schmall JP, Werner ME and Daube-Witherspoon ME. PennPET explorer: design and preliminary performance of a whole-body imager. J Nucl Med. 2020: 61 136–43.

4. Prenosil GA, Sari H, Fürstner M, Afshar-Oromieh A, Shi K, Rominger A, Hentschel M. Performance Characteristics of the Biograph Vision Quadra PET/CT system with long axial field of view using the NEMA NU 2-2018 Standard. J Nucl Med. 2022; 63(3):476–484.

5. Mikhaylova E, Tabacchini V, Borghi G, Mollet P, D’Hoe E, Schaart DR, Vandenberghe S. Optimization of an ultra-dose high-resolution pediatric PET scanner design based on monolithic scintillators with dual-sided digital SiPM readout: a simulation study. Phys Med Biol. 2017; 62:8402–8418.

6. Mariele Stockhoff, Milan Decuyper, Roel Van Holen, Stefaan Vandenberghe. High-resolution monolithic LYSO detector with 6-layer depth-of-interaction for clinical PET. Phys Med Biol. 2021; 66(15):10.1088/1361-6560.

## P19 PET scanners based on pure Cherenkov detectors: a Monte Carlo Study

### Gašper Razdevšek^1*^, Rok Pestotnik^2^, Peter Križan^1,2^, Samo Korpar^2,3^, Andrej Seljak^2^, Andrej Studen^1,2^, Rok Dolenec^1,2^

#### ^1^Faculty of Mathematics and Physics, University of Ljubljana, Ljubljana, Slovenia; ^2^Jožef Stefan Institute, Ljubljana, Slovenia; ^3^Faculty of Chemistry and Chemical Engineering, University of Maribor, Maribor, Slovenia

##### **Correspondence:** Gašper Razdevšek (gasper.razdevsek@fmf.uni-lj.si)

*EJNMMI Physics* 2023, **10(1):**P19

**Background**

The detection of annihilation photons in PET can be based on detecting Cherenkov photons instead of scintillation light. Dense Cherenkov radiators provide an opportunity for high gamma detection efficiency—due to their high stopping power and photofraction—and excellent CTR.

**Methods**

We first performed a simulation study of different Cherenkov detector designs based on PbF_2_ with SiPMs as photosensors (realistic PDE and SPTR, but the noise was omitted), where a 2-sided readout was also considered. Next, we selected a few of these designs to model whole-body Cherenkov PET systems. The performance of Cherenkov PET scanners was evaluated and compared to the reference scanner—our model of Siemens Biograph Vision clinical PET scanner. We assessed and compared count rates and image quality of the PET scanners following the NEMA NU 2–2018 standard. Monte Carlo simulations were performed on a super-computing network using GATE, and CASToR software was used for TOF-OSEM image reconstruction. Normalization, scatter, random, and attenuation correction factors were included in the reconstruction. Finally, we evaluate the image quality achieved by the extended scanners (106 cm AFOV) using the NEMA and XCAT digital phantoms.

**Results**

Cherenkov scanner with 1-sided readout had similar TOF performance (225 ps CTR-FWHM) and achieved very similar image quality as the reference scanner (Fig. 1, 2). By using a 2-sided readout, higher coincidence detection efficiency and better CTR was obtained, resulting in better image quality (Fig. 3).

**Conclusion**

Our Monte Carlo simulations show that even though pure Cherenkov scanners have basically no energy resolution (but have some intrinsic suppression), the scatter fraction of around 50% is not prohibitively large, and images comparable to a modern clinical PET scanner can be achieved if the noise of the photodetector is sufficiently low. Low-cost Cherenkov detectors (PbF_2_ ~ 1/9 LSO price) could become especially interesting for total-body scanners.

**Fig. 1:**
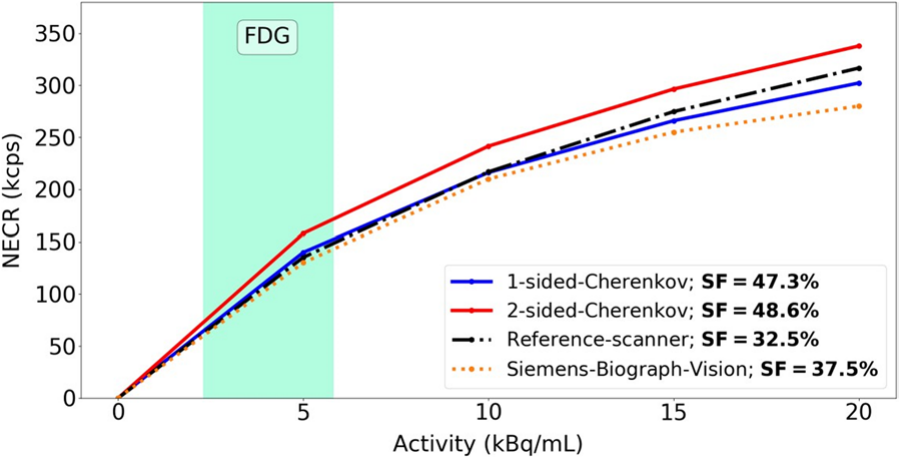
NECR values as a function of the activity: a comparison between the Cherenkov scanners, the simulated reference scanner, and measurements performed on Siemens Biograph Vision. The scatter fractions (SF) of different scanners are shown in the legend. The shaded rectangle shows the typical range of activities used at the start of clinical FDG whole-body scans.

**Fig. 2:**
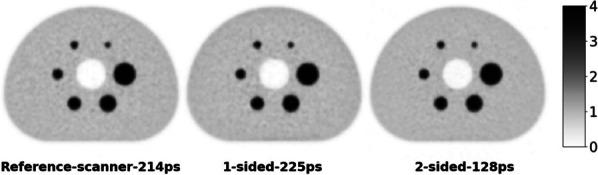
Transverse views of the reconstructed images of the NEMA image quality phantom for different scanners. The ratio between the hot spheres and the background was 4:1. A Gaussian post-filter with 5 mm FWHM was applied on all images.

**Fig. 3:**
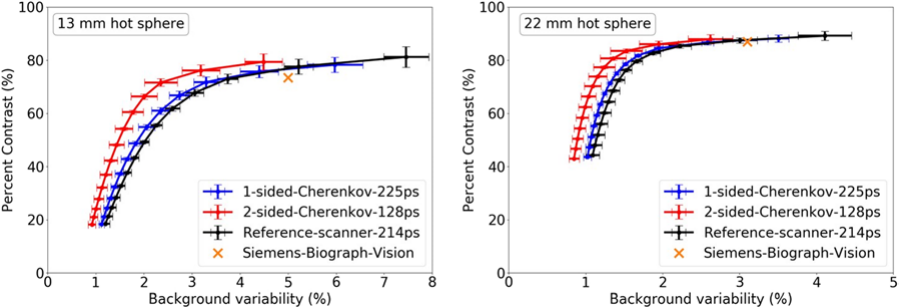
Percent contrast vs background variability for a 13 mm and 22 mm diameter hot sphere. Gaussian post-filters with different widths were used to vary the background variability. The measured value of the Siemens Biograph Vision scanner is added for reference.

## P20 Exploration of the physical limits for Cherenkov positron emission tomography using tiny crystals and a large cube

### Sofiia Forostenko^1*^, Werner Lustermann^1^

#### ^1^Institute for Particle Physics and Astrophysics, ETH Zurich, Zurich, 8093, Switzerland

##### **Correspondence:** Sofiia Forostenko (sofiiaf@ethz.ch)

*EJNMMI Physics* 2023, **10(1):**P20

This study is performed within the scope of the project “Microelectromechanical systems-based gamma-ray detectors for time-of-flight positron emission tomography (PET)”, which aims at developing a new photo-sensor with 10 picoseconds full width at half maximum single-photon timing resolution, capable of exploiting the prompt nature of Cherenkov radiation. We performed Monte Carlo simulation study to estimate the possible improvement in the overall performance of the whole-body PET scanner built out of the photo-sensors developed by our project collaborators.

Using Geant4, we estimated the physical limits of timing resolution for two possible detector geometries: a radiator of 3 × 3 × 3 mm^3^ with one photo-sensor attached opposite to the side the gamma enters, and a 25 × 25 × 25 mm^3^ cube with photo-sensors fully covering all six sides. The 25 × 25 × 25 mm^3^ cube required reconstruction of the gamma interaction position that is performed using arrival times and the Cherenkov photons detection positions, minimizing a cost function for the gamma interaction position. Monte Carlo information confirmed the reconstruction of the gamma interaction position inside the cube. We simulated a whole-body PET system with GATE (Geant4 Application for Tomographic Emission). As a result, the spatial resolution in three dimensions of the point source was obtained reconstruction-less.

## P21 A deep learning method for the recovery of standard-dose imaging quality from ultra-low-dose PET

### Song Xue^3*^, Hong Zhu^5^, Rui Guo^2^, Hasan Sari^3,4^, Clemens Mingels^3^, Konstantinos Zeimpekis^3^, George Prenosil^3^, Marco Viscione^3^, Raphael Sznitman^6^, Axel Rominger^3^, Biao Li^1,2^, Kuangyu Shi^3,5^

#### ^1^Department of Nuclear Medicine, Ruijin Hospital, Shanghai Jiao Tong University School of Medicine, Shanghai, China; ^2^Collaborative Innovation Center for Molecular Imaging of Precision Medicine, Ruijin Center; ^3^Department of Nuclear Medicine, University of Bern, Switzerland; ^4^Advanced Clinical Imaging Technology, Siemens Healthcare AG, Lausanne, Switzerland; ^5^Department of Informatics, Technical University of Munich, Germany; ^6^ARTORG Center, University of Bern, Switzerland

##### **Correspondence:** Song Xue (song.xue@dbmr.unibe.ch)

*EJNMMI Physics* 2023, **10(1):**P21

**Introduction**

Recent development in positron emission tomography (PET) dramatically increased the effective sensitivity by increasing the geometric coverage leading to total-body PET imaging. This encouraging breakthrough brings the hope of ultra-low dose PET imaging equivalent to transatlantic flight with the assistance of deep learning (DL)-based methods. A critical bottleneck for conventional DL-based methods is their limited capability in the application in the heterogeneous domain of PET imaging. We aim to develop a DL method that can recover high-quality imaging from ultra-low-dose PET.

**Methods**

Total-body PET images of 550 patients using ^18^F-FDG, ^18^F-PSMA, ^68^Ga-DOTA-TOC, ^68^Ga-DOTA-TATE, acquired using total-body PET scanners, including Biograph Vision Quadra (Siemens Healthineers), uEXPLORER (United Imaging) in Shanghai and Bern, were included for the development and testing of the proposed method. A conditional generative adversarial network (GAN) was customized for cross-scanner and cross-tracer optimization. The effectiveness and robustness of our proposed approach was verified in tests of different imaging tracers on different scanners.

**Results**

The developed DL method can achieve a good preliminary physical performance with an average whole-body normalized root mean squared error (NRMSE) of 0.09% ± 0.02%, peak signal-to-noise ratio (PSNR) of 60.9 ± 2.3, and structural similarity index measurement (SSIM) of 0.99 ± 0.001, when comparing the DL enhanced PET images to full dose images at dose reduction factor (DRF) = 10. Exemplary test results are shown in Fig. 1.

**Conclusion**

The proposed DL approach for low-dose PET image enhancement had the potential to be applied on different scanners and tracers, which can improve the performance and robustness of image quality recovery on ultra-low-dose PET imaging. It may improve the trustworthiness and clinical acceptability of DL-based dose reduction.

**Fig. 1:**
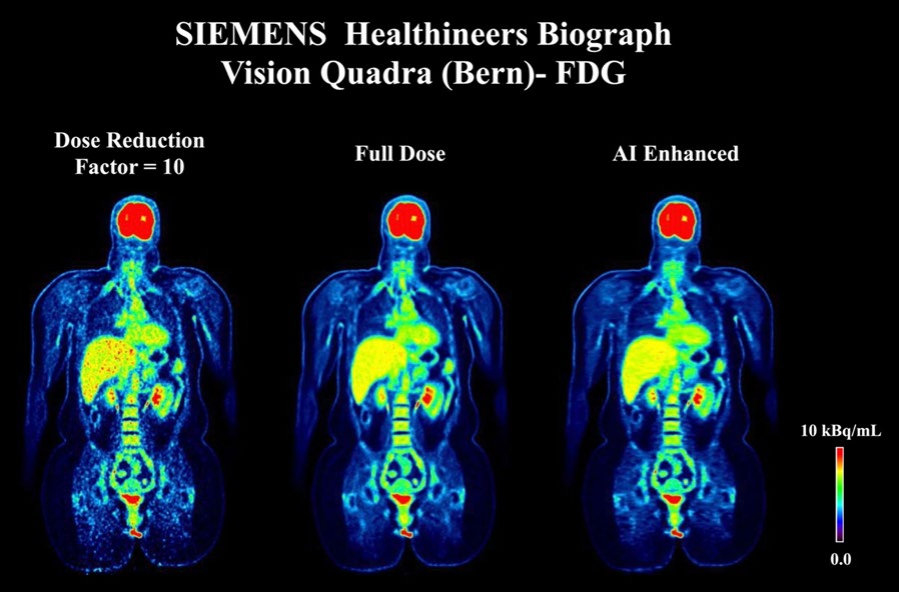
Exemplary test results of ^18^F-FDG imaging from SIEMENS Healthineers Biograph Vision Quadra.

## P22 Application of machine learning methods for coincidence classification in the large field-of view J-PET scanner

### Wojciech Krzemień^1*^, Konrad Klimaszewski^2^ on behalf of the J-PET Collaboration

#### ^1^High Energy Physics Division, National Centre for Nuclear Research, PL-05-400 Otwock, Swierk, Poland; ^2^CIS, National Centre for Nuclear Research, PL-05-400 Otwock, Swierk, Poland

##### **Correspondence:** Wojciech Krzemień (wojciech.krzemien@ncbj.gov.pl)

*EJNMMI Physics* 2023, **10(1):**P22

**Background**

Recent PET development is the introduction of total-body PET scanners. The J-PET collaboration has elaborated a concept of a cost-effective, long scanner built from axially arranged plastic scintillators^[1]^.

The reconstruction of the spatial distribution of the radiotracer in patient’s body is based on the photon pairs grouped into time coincidences. Due to the limited resolution the selected coincidences contain a fraction of events with a photon scattered in the patient or photons accidentally registered in a coincidence. Scatters and accidentals deteriorate the final image quality. To mitigate this, corrections are incorporated into the image reconstruction. Alternatively, a pre-correction procedure can be applied.

For a total-body scanner, the background level becomes a challenge. First, the accidentals statistics increase roughly quadratic with the scanner axial length. Also, the multiply scattered photons fraction is more pronounced. In J-PET scanner the signal registration is based on the Compton scattering process, which makes the inter-detector scatters harder to discriminate.

**Materials and methods**

We apply supervised learning models to estimate the background contribution. In particular, boosted decision trees and deep learning neural networks are considered.

The training and test samples are based on GATE MC simulations. Both the NEMA-IEC and XCAT phantoms are simulated with several scanner models. Selection of optimal feature set and feature transformations is performed.

**Results**

Performance of XGBoost, AdaBoost and NN classifiers is compared with cut-based selection criteria. The estimated distributions are compared to the standard estimation methods e.g. delayed time window. Considered models are compared based on efficiency metrics. Finally, comparison of reconstructed image quality is provided.

**Conclusions**

The application of ML methods can efficiently improve the signal-to-noise ratio. Too restrictive selection leads to the reduction of statistics and deterioration of image quality. Future research includes studies of model generalization with respect to different detector geometries and phantom shapes.

**Acknowledgements**

The aforementioned work is presented on behalf of the J-PET Collaboration.

**References**

[1] P. Moskal, K. Dulski, et al., “Positronium imaging with the novel multiphoton PET scanner,” Sci. Adv., vol. 7,42, eabh4394, 2021.

## P23 Commissioning of 50 cm AFOV modular plastic J-PET scanner

### Szymon Niedźwiecki*

#### Faculty of Physics, Astronomy and Applied Computer Science Jagiellonian University, 30-348 Kraków, Poland

##### **Correspondence:** Szymon Niedźwiecki (szymon.niedzwiecki@uj.edu.pl)

*EJNMMI Physics* 2023, **10(1):**P23

**Background**

PET imagining community is in progress of developing total-body PET systems in order to increase the sensitivity of detection system by enlarging the axial field-of-view (AFOV). Achieving higher sensitivity can decrease the time of scan or the injected dose. One of the approaches to increase AFOV is to increase the amount of detection rings while another, cost-effective possibility is to detect gamma quanta by means of plastic scintillators, orientated along the patients body [1, 2]. Such solution maintains the size of readout system, independently on AFOV. Here a prototype with 50 cm AFOV will be presented, which is the first step towards the total-body PET based on plastic strips.

**Materials and methods**

A mobile, modular prototype of the J-PET detector, a PET scanner based on plastic scintillators, is being commissioned at the Jagiellonian University this year. The system is built out of 24 modules, composed of 50 cm long 13 scintillator strips, read out by a SiPMs (Fig. 1).

Each module converts an analogue response to the digital data independently. Acquisition system is based on trigger-less FPGA technology divided into few steps, maintaining the modularity of the system: TDC conversion based on MVT by means of LVDS buffers [3], concentration of signals from each end of the module and final aggregation of data streams with possibility to produce initial image in real-time [4].

**Results**

General characterization of whole system performance will be discussed, as well as preliminary time and spatial resolutions of the setup along with the precision of time measurement of electronic boards.

**Conclusions**

Commissioning of the 50 cm FOV modular plastic J-PET scanner has been completed at the Jagiellonian University and preliminary checks show that it is ready to perform tests on clinical patients. First in-vitro images using the J-PET prototype have been recently demonstrated [5, 6].

**Fig. 1:**
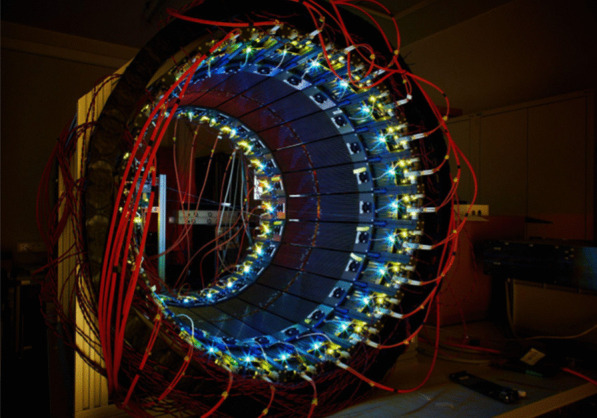
Mobile, modular J-PET prototype, built out of 24 modules. Each module is composed of 13 scintillator strips, read out by a SiPM array from both sides.

**Acknowledgements**

The above mentioned work is presented on behalf of the J-PET Collaboration. This work is supported by the Foundation for Polish Science under Grant TEAM/2017–4/39 and “Laboratories of the Young” as a part of “Excellence Initiative—Research University” program at the Jagiellonian University in Krakow LM/6/NS.

**References**

1. P. Moskal, et al. “Simulating NEMA characteristics of the modular total-body J-PET scanner—an economic total-body PET from plastic scintillators”, Phys. Med. Biol. 66 (2021) 175015.

2. S. Niedźwiecki, et al., “J-PET: A New Technology for the Whole-body PET Imaging”, Acta Phys. Polon. 2017; B48 10: 1567–1576.

3. M. Pałka, et al., “Multichannel FPGA based MVT system for high precision time (20 ps RMS) and charge measurement”. JINST; 2017: 12 P08001.

4. G. Korcyl, et al. “Evaluation of Single-Chip, Real-Time Tomographic Data Processing on FPGA–SoC Devices”. IEEE Transactions On Medical Imaging. 2018; Vol. 37, No. 11: 2526–2535.

5. P. Moskal, et al., “Positronium imaging with the novel multiphoton PET scanner”, Science Advances 7 (2021) eabh4394.

6. P. Moskal, et al., “Testing CPT symmetry in ortho-positronium decays with positronium annihilation tomography”, Nature Communications 12 (2021) 5658.

## P24 Evaluation of CT dose reduction scanning a whole-body phantom with the Biograph Vision Quadra PET-CT

### Samaneh Mostafapour^1^, James Hamill^2^, Adriaan A. Lammertsma^1^, Charalampos Tsoumpas^1^

#### ^1^Medical Imaging Center, University Medical Center Groningen, Groningen, Netherlands; ^2^Siemens Medical Solutions, USA

##### **Correspondence:** Samaneh Mostafapour (s.mostafapour@umcg.nl)

*EJNMMI Physics* 2023, **10(1):**P24

**Background**

Low-dose CT can provide about 1–3 mSv of the total delivered radiation dose during a typical PET-CT scan. Although this may not be a substantial amount of the total dose received by the patient in conventional PET-CT scans. The development of long-axial field-of-view scanners can allow for PET imaging with much lower injected dose, therefore reduction of dose delivered from the CT become more essential [1,2]. In this study, we made use of the Siemens Quadra PET-CT and investigated how to modify the CT protocol for ultra-low dose. We evaluated the amount of CT dose reduction using the extra available CT tin filter and by changing the tube current.

**Materials and methods**

An anthropomorphic whole-body phantom underwent a CT study by a Biograph Vision Quadra PET-CT scanner three times using different CT parameters. Two scans were conducted using the same tube current (30mAs) although one of them used the Tin filter. Also, one scan was done using a different tube current (7mAs) and without the Tin filter. The Contrast to noise ratios (CNRs) and Signal to noise ratios (SNRs) were calculated. Also, the effective dose for each scan was calculated based on the reported CTDIvol.

**Results**

The lowest effective dose (0.175 mSv) is related to the CT scan with the Tin filter (Table1). The CT image with the tube current of 7mAs showed a higher CNR (26.22) and SNR (10.19) (Fig. 1) and a better correlation (R^2^ = 0.98) to the normal-dose CT compare to the CT scan with the Tin filter (R^2^ = 0.87) (Fig. 2).

**Conclusions**

Using the Tin filter during the PET-CT studies can reduce the delivered radiation dose to the patients; still, the image quality decreases significantly compared to other dose reduction techniques. Future studies are required to assess the effect of this dose reduction on PET quantification in the clinical datasets.

**Fig. 1:**
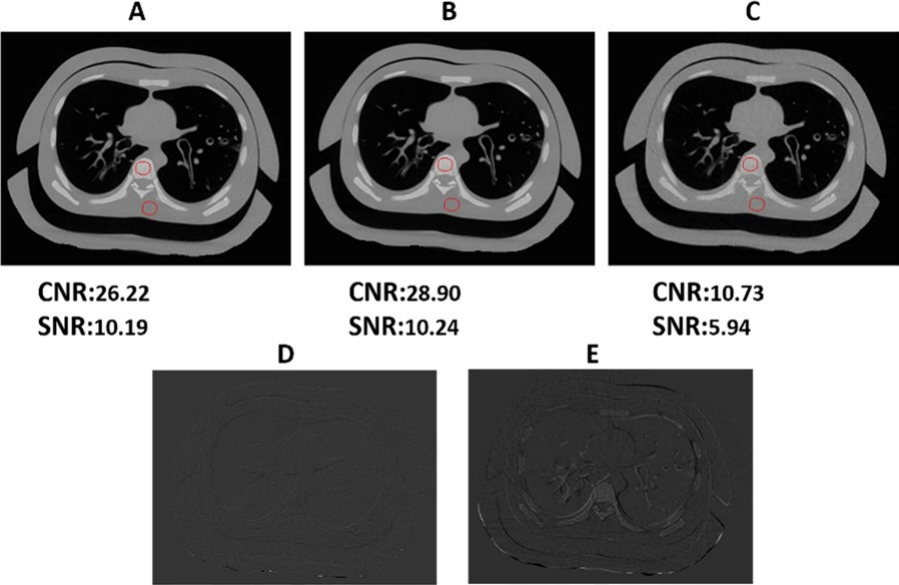
Representative axial views of CT image using **A** mAs 7, **B** mAs 30, **C** mAs 30, and Tin filter along with the subtracted images with respect to the reference CT image; **D** the subtraction of CT mAs-30, CT mAs-7, and **E** the subtraction of CT mAs-30, CT mAs-30-Tin filter. The CNR and SNR values for each slice are available.

**Fig. 2:**
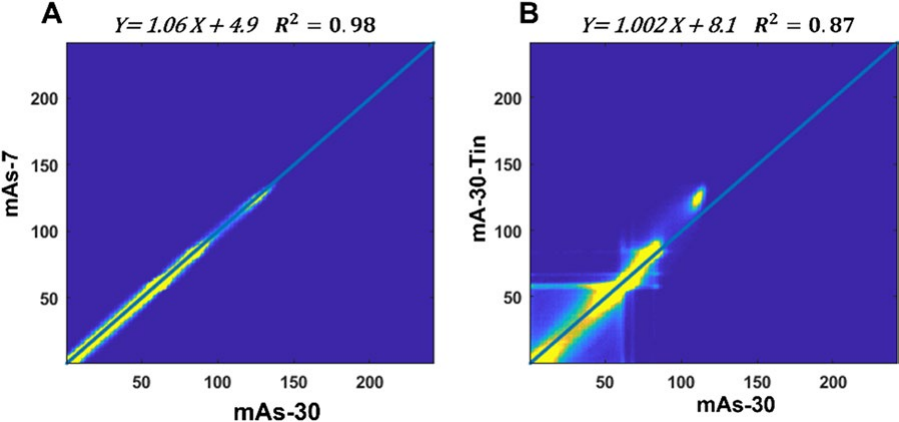
Voxel-wise joint correlation histogram analysis of the two low-dose CT scans versus the normal-dose CT image: CT scan using the (A) mAs 7, (B) mAs 30, and Tin filter.

**Table 1:** Used parameters for each CT scan and their calculated Dose, CNR, and SNR values.

	mAs-30	mAs-7	mAs-30-Tin
ref mAs	30	7	30
CARE kV	100	100	100
Pitch	1.1	1.1	1.1
Collimation (mm)	32 × 1.2	32 × 1.2	32 × 1.2
Tin filter	No	No	Yes
CTDIvol (mGy)	1.471	0.353	0.114
DLP (mGy.cm)	155.82	37.418	11.66
Effective dose (mSv)	2.33	0.561	0.175
SSDE (mGy)	1.77	0.43	0.13
CNR	28.90	26.22	10.73
SNR	10.24	10.19	5.94

**References**

1. White paper. Dose reduction techniques for CT-based PET attenuation correction. White Paper – Siemens Healthcare GmbH, Germany. 2021

2. Suntharalingam S, Mikat C, Wetter A, Guberina N, Salem A, Heil P, Forsting M, Nassenstein K. Whole-body ultra-low dose CT using spectral shaping for detection of osteolytic lesion in multiple myeloma. European Radiology. 2018 Jun;28(6):2273–80.

## P25 Exploiting AI-enhanced BGO detectors for total-body rat preclinical imaging

### Luigi Masturzo^1,2^, Pietro Carra^1,2^, Nikos Efthimiou^3^, Matteo Morrocchi^1,2^, Edoardo Pasca^4^ Andrew J Reader^5^, Giancarlo Sportelli^1,2^, Kris Thielemans^6^ and Nicola Belcari^1,2*^

#### ^1^Department of Physics “E.Fermi”, University of Pisa, Pisa, Italy; ^2^National Institute of Nuclear Physics (INFN), Pisa Section, Pisa, Italy; ^3^Athinoula A. Martinos Center for Biomedical Imaging, Massachusetts General Hospital, Harvard Medical School, Charlestown, MA, USA; ^4^Scientific Computing Department, STFC, UKRI, Rutherford Appleton Laboratory, Didcot, UK; ^5^School of Biomedical Engineering and Imaging Sciences, King’s College London, UK; ^6^Institute of Nuclear Medicine, University College London, UK

##### **Correspondence:** Nicola Belcari (nicola.belcari@gmail.com)

*EJNMMI Physics* 2023, **10(1):**P25

**Background**

This work presents a Monte Carlo characterization of a total-body rat preclinical scanner obtained through experimental detector characterization and simulated phantom acquisitions.

The PET modules are based on the UTOFPET BGO-based detectors in which the timestamp and event position are obtained via a neural network. The aim is to combine both the state-of-the-art detector specifications and the excellent sensitivity given by the long axial coverage to obtain good performance in terms of image quality.

**Materials and methods**

The scanner has a diameter of 112.8 mm and an axial field of view of 357 mm. It is composed of 7 rings of 8 monolithic detectors. Each detector consists of a 51 × 51 × 12 mm^3^ BGO crystal read by 64 SiPMs of 6 mm pitch. The simulations were performed using GATE. Simulation parameters were obtained through experimental characterization of the UTOFPET detector. An event position blurring of 1.1 mm FWHM (X,Y) and 1.8 mm FWHM (DOI) inside the crystal was applied in order to model the experimentally measured intrinsic spatial resolution of the detector and an energy resolution of 15.8% was applied. Reconstruction by an ordered-subset expectation–maximization (OSEM) algorithm, as well as backprojection/histoimage deep-learned reconstruction, are both under active consideration.

**Results**

The scanner provides a peak of sensitivity of 31.9% (energy window = 350–650 keV) and a noise equivalent count rate (NECR) peak of 717 kcps at 14 MBq. Early characterization results with limited angle reconstruction proved a spatial resolution of about 1.2 mm FWHM.

**Conclusions**

Exploiting the AI-enhanced positioning events algorithm on BGO crystals, it is possible to obtain state-of-the-art results in terms of spatial resolution and count rate capabilities together with an unprecedented sensitivity opening new possibilities for dynamic/quantitative preclinical PET imaging.

## P26 A pathway to studying pulmonary and nasal drug delivery using total-body PET

### Avshalom Offner^*^, Jacques Vanneste

#### School of Mathematics and Maxwell Institute for Mathematical Sciences, The University of Edinburgh, Edinburgh, EH9 3FD, UK

##### **Correspondence:** Avshalom Offner (avshalom.offner@ed.ac.uk)

*EJNMMI Physics* 2023, **10(1):**P26

**Background**

Visualisation of fluid flow in the human body provides critical data for diagnosis of various diseases, e.g., gallstones, kidney failure, and CVDs. Existing diagnostic methods (ultrasound, MRI, PET, and others) visualise the flow of liquids only, while tools to visualise the flow of air and matter suspended in it are currently not available. This limits our understanding of pulmonary and nasal drug delivery, which is crucially dependent on the fluid dynamics of drug aerosols in the respiratory tract and nasal cavity, respectively. In the present work, we propose the use of Positron Emission Particle Tracking (PEPT)—a variation of PET in which radiolabelled particles serve as tracers of fluid motion—to study flows through these routes.

**Methods**

Given the small scale of the respiratory and nasal passages, high accuracy is required for the reconstruction of suspended particulate trajectories. To ensure this accuracy is reached, we formulate a probabilistic mathematical model and use it to quantify this uncertainty by employing Bayesian inference – a well-known method to solve high-dimensional inverse problems.

**Results**

Our analysis quantifies the relative contribution of scanner geometry to the uncertainty, revealing that increasing the field of view (FOV) from L/D = 0.3 (Siemens Biograph) to L/D = 2.5 (uExplorer), where L and D are the scanner length and diameter, dramatically decreases the uncertainty as shown by the solid angle calculation in Fig. 1.

**Conclusions**

The increased FOV dramatically increases the number of lines of response recorded, allowing us to infer each momentary position using much shorter time intervals, over which particles only slightly move. The results suggest that total-body PET scanners can greatly improve our understanding of pulmonary and nasal drug delivery.

**Fig. 1:**
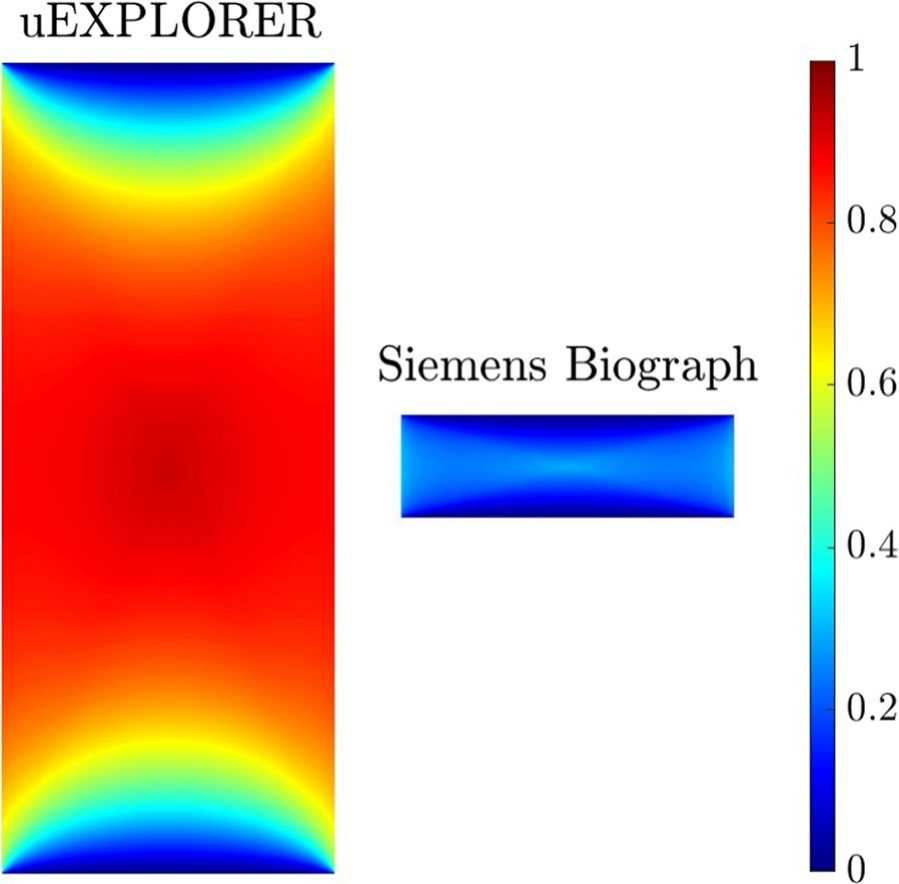
Solid angle over a cross-section of a total-body (left) and a standard (right) PET scanners, as calculated using our mathematical model. The total-body PET scanner efficiency is 2–10 times higher than its counterpart.

## P27 Practice of domain knowledge towards robust and generalizable deep learning-based CT-free attenuation and scatter correction for an ultra-long axial field of view PET scanner

### Song Xue^3*^, Rui Guo^2^, Hasan Sari^3,4^, Clemens Mingels^3^, Konstantinos Zeimpekis^3^, George Prenosil^3^, Marco Viscione^3^, Raphael Sznitman^6^, Axel Rominger^3^, Biao Li^1,2^, Kuangyu Shi^3,5^

#### ^1^Department of Nuclear Medicine, Ruijin Hospital, Shanghai Jiao Tong University School of Medicine, Shanghai, China; ^2^Collaborative Innovation Center for Molecular Imaging of Precision Medicine, Ruijin Center; ^3^Department of Nuclear Medicine, University of Bern, Switzerland; ^4^Advanced Clinical Imaging Technology, Siemens Healthcare AG, Lausanne, Switzerland; ^5^Department of Informatics, Technical University of Munich, Germany; ^6^ARTORG Center, University of Bern, Switzerland

##### **Correspondence:** Song Xue (song.xue@dbmr.unibe.ch)

*EJNMMI Physics* 2023, **10(1):**P27

**Introduction**

The possibility of low-dose positron emission tomography (PET) imaging using high sensitivity long axial field of view (FOV) PET/computed tomography (CT) scanners makes CT a critical radiation burden. Deep learning (DL)-based methods have been proposed to substitute CT-based PET attenuation and scatter correction to achieve CT-free PET imaging. A critical bottleneck for these DL-based methods is their limited capability in the application in the heterogeneous domain of PET imaging, i.e. a variety of scanners and tracers. This study employs a simple way to integrate domain knowledge in deep learning for CT-free correction for a long axial FOV PET scanner.

**Methods**

In contrast to conventional direct deep learning methods, we simplify the complex problem by a domain decomposition so that the learning of anatomy-dependent attenuation correction can be achieved robustly in a low-frequency domain while the original anatomy-independent high-frequency texture can be preserved during the processing. The effectiveness and robustness of our proposed approach was verified in tests of external imaging tracers on different scanners. Whole body PET images of 829 patients using ^18^F-FDG, ^18^F-PSMA, ^68^Ga-DOTA-TOC, ^68^Ga-DOTA-TATE, ^68^Ga-FAPI, acquired using clinical PET scanners, including Biograph Vision (Siemens Healthineers), United Imaging uMI 780 (United Imaging), Discovery MI (General Electric Healthcare) in Shanghai and Bern, were included for the development and testing of the proposed method.

**Results**

Although the method was developed using one tracer and one scanner, it achieved an average whole-body normalized root mean squared error (NRMSE) and peak signal-to-noise ratio (PSNR) of 0.3% ± 0.2% and 51.5 ± 6.4 respectively for different scanners, and 0.6% ± 0.4% and 47.5 ± 7.4 for different tracers, which have significantly improved over conventional deep learning methods.

**Conclusion**

The proposed decomposition-based method provides a simple approach to incorporating domain knowledge in deep learning, which can significantly improve the performance and robustness of CT-free PET correction.

**Fig. 1:**
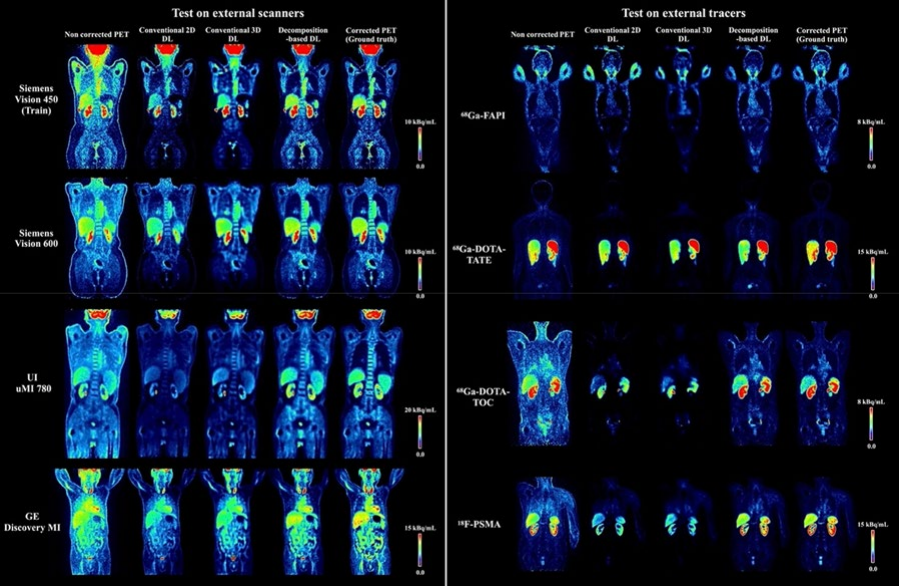
Exemplary test results of external imaging tracers on different scanners.

**Fig. 2:**
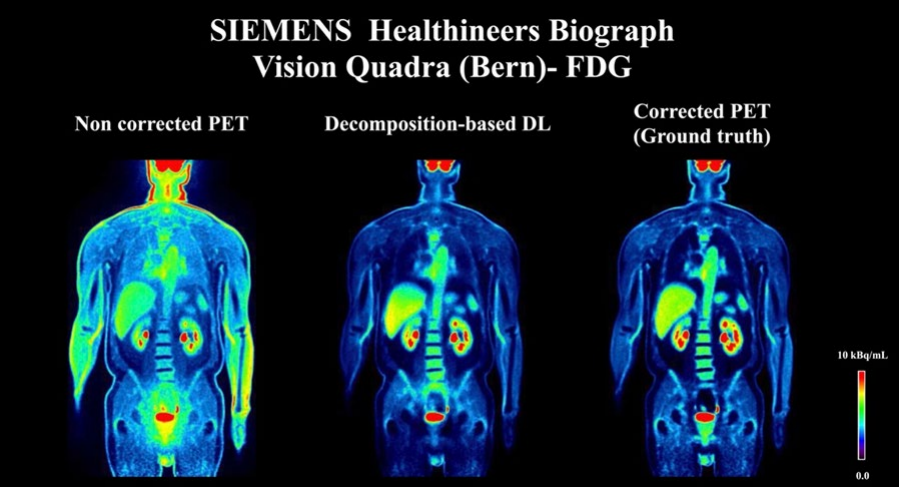
Exemplary test results of ^18^F-FDG imaging from SIEMENS Healthineers Biograph Vision Quadra.

**Fig. 3:**
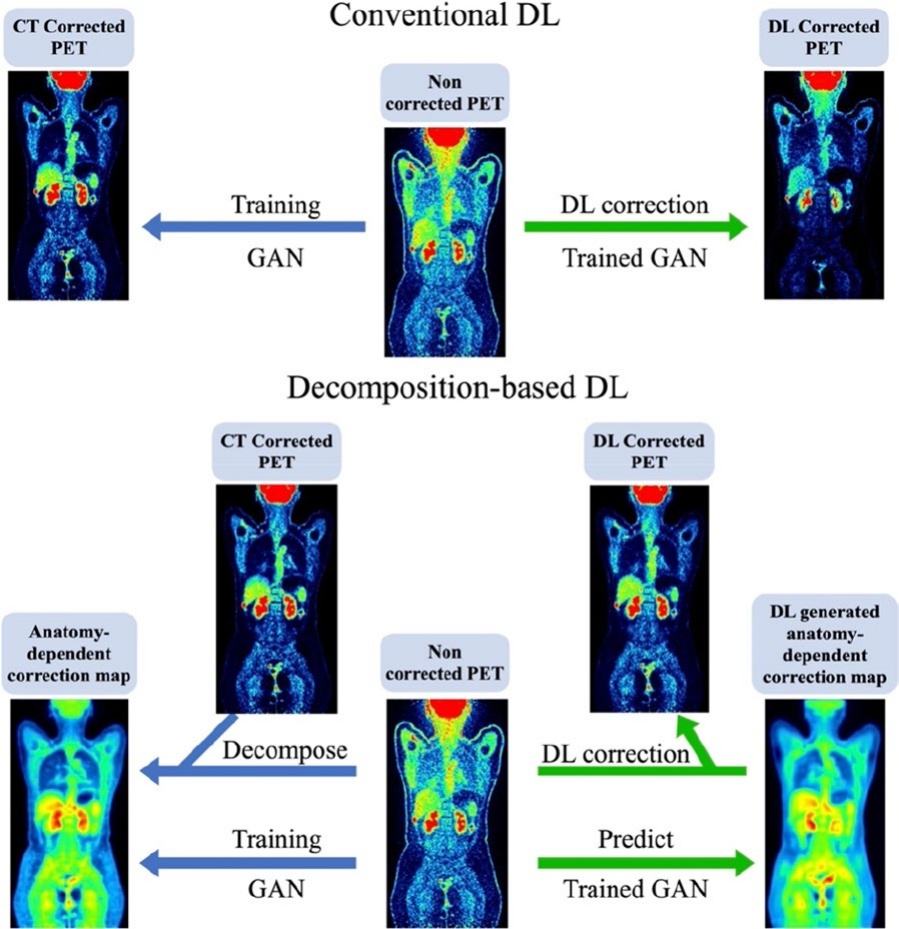
General protocol of our proposed domain knowledge integrated deep learning method.

## P28 Design of a generic method for single acquisition dual-tracer PET imaging

### Nasrin Taheri^1,2^*, Benjamin Le Crom^2^, Caroline Bouillot^3^, Michel Chérel^1^, Nicolas Costes^3^, Sébastien Gouard^1^, Séverine Marionneau-Lambot^1^, Thibaut Merlin^4^, Dimitris Visvikis^4^, Simon Stute^1,2^ and Thomas Carlier^1,2^

#### ^1^CRCINA, INSERM, CNRS, Université d’Angers, Université de Nantes, Nantes, France; ^2^Nuclear Medicine Department, University Hospital, Nantes, France; ^3^CERMEP, INSERM, Lyon, France; ^4^LaTIM, INSERM, Brest, France

##### **Correspondence:** Nasrin Taheri (Nasrin.taheri@chu-nantes.fr)

*EJNMMI Physics* 2023, **10(1):**P28

**Context**

Total body Positron Emission Tomography (PET) opens an area of feasible large axial single PET acquisition with quasi-simultaneous dual-tracer injection. Dual-tracer acquisitions allow to eliminate physiological changes and external or internal movements that would occur between two independent scans separated by several days. The main goal of this study is to develop a robust technique to reconstruct two separate PET dynamic images from a single dataset regardless of any clinical scenarios with the least number of assumptions and parameters.

**Methodology**

The Arterial *Input Function (AIF)* can be extracted from Image-derived Input Function (IDIF) and modeled linearly using three exponential terms.

The generic and linear spectral model in combination with the NNLS algorithm are used to quantify the dynamic PET. The net uptake of the tracer ($K_{net}$) is estimated for each realization.

**Simulations**

Realistic synthetic data were simulated in order to evaluate the performance of our approach; Time Activity Curves (TACs) of [18F]-FCH and [18F]-FDG were generated using kinetic parameters from the literature. Two-tissue compartmental model is used to create noise-free tissue TACs and Gaussian noise is added to some extent. Different injection delays were considered within an acquisition of 60 min.

**Results and discussion**

To visualize the performance of the presented approach, the error of estimating $K_{net}$ is provided with single or dual-tracer injection in Fig. 1. Compared to the advantage of the proposed system, the error of dual-tracer is acceptable for an injection delay of 20 min. The essential advantage of this model lies in the fact that the least number of assumptions and tuning parameters is required. Preclinical experiments were performed and are currently being processed. The results will be presented at the conference.

**Fig. 1:**
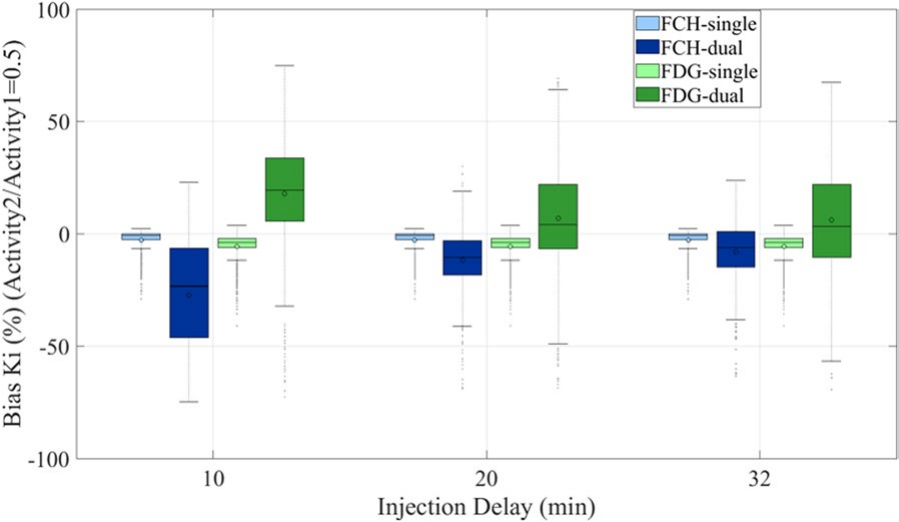
Bias of $K_{net}$ estimations for the case of single and dual-tracer in different injection delays.

## P29 Generation of a patient-specific arterial input function from late dynamics at the mCT and Vision Quadra

### Sebastian von Beschwitz^1*^, Dominik Blum^1^, Fabian Schmidt^1,2^, Jürgen Kupferschläger^1^, Christian la Fougère^1^

#### ^1^Department of Nuclear Medicine and Clinical Molecular Imaging, University Hospital Tuebingen, Tuebingen, Germany; ^2^Werner Siemens Imaging Center, Department of Preclinical Imaging and Radiopharmacy, Eberhard-Karls University Tuebingen, Tuebingen, Germany

##### **Correspondence:** Sebastian von Beschwitz (Sebastian.Beschwitz@med.uni-tuebingen.de)

*EJNMMI Physics* 2023, **10(1):**P29

**Background**

Parametric imaging (metabolic rate and volume of distribution) on the Biograph mCT and on the Vision Quadra (Siemens) currently requires long scan durations up to 80 min. We aim to shorten the the scan duration by deriving the entire patient-specific arterial input function (AIF_id_) from late dynamics.

**Material and methods**

32 patients were dynamically examined with [F-18]-FDG at the mCT using Flowmotion technique over 80 min p.i. Image-derived population-based input functions (PBIF) were generated from these exams. AIF_id_ was calculated by normalizing the patient-specific blood activity concentrations of the last 30 min to the late dynamics of gender-specific PBIF, followed by a weighting. Using the implemented AIF (Siemens Patlak Suite) and AIF_id_ (Fig. 1), Patlak reconstructions were performed and analyzed quantitatively and qualitatively (Fig. 2).

**Results**

A good agreement between AIF and AIF_id_ was found with an area under curve (AUC) difference of 1.7% ± SD 5.3%. We observed a small difference in the comparison of AIF to AIF_id_ in metabolic rate (K_i_) and volume of distribution (V_d_) of 0.3+ − 12.5% and 3.4%+ − 15.8, respectively.

**Conclusions**

We showed that a reduction of the scan time to 30 min is feasible with our method for the mCT. We are currently transferring and evaluating this method for the Quadra using the Patlak reconstruction of the e-7 tools. Based on the better image quality of the Quadra due to higher sensitivity, temporal and spatial resolution, we aim to further shorten the acquisition time and reduce the influence of the AUC by more accurate placement of the VOI in the aorta for derivation of the AIF.

**Fig. 1:**
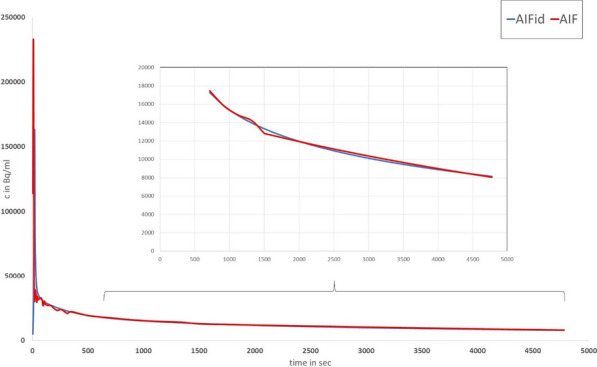
Comparison of implemented AIF and calculated AIF_id_.

**Fig. 2:**
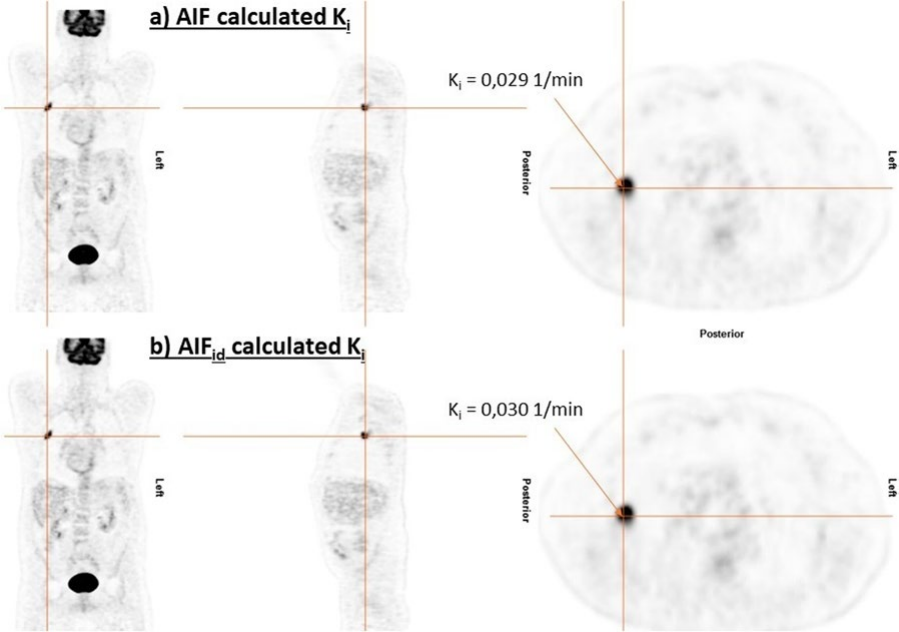
Qualitative comparison of Patlak images based on **a** AIF (K_*i,max*_=0.144 1/min) and **b** AIF_id_ (K_*i,max*_=0.147 1/min).

## P30 Assessment of metastasis lesion detection by means of Total-Body Jagiellonian Positron Emission Tomography scanner

### Meysam Dadgar^1,2^*, Pawel Moskal^1,2^

#### ^1^Faculty of Physics, Astronomy, and Applied Computer Science, Jagiellonian University, Łojasiewicza11, 30-348 Kraków, Poland; ^2^Total Body Jagiellonian-PET Laboratory, Jagiellonian University, Kraków, Poland

##### **Correspondence:** Meysam Dadgar (meysam.dadgar@doctoral.uj.edu.pl)

*EJNMMI Physics* 2023, **10(1):**P30

**Background**

Metastasis which accounts for 90% of cancer-related deaths occurs when cancer cells detach from their primary site in distant organs. Larger lesions can be detected through non-invasive clinical imaging modalities such as MRI, CT, and PET scan [1]. Although these imaging modalities are capable of detecting large lesions caused by metastases, they do not offer the required sensitivity to detect small lesions caused by the early spread of metastatic tumor cells [2]. Nowadays there is an intense development of a novel generation of the Total-Body PET tomographs, able to provide higher sensitivity due to their larger axial field of view. Jagiellonian PET collaboration is developing a new cost-efficient generation of Total-Body PET based on plastic scintillators [3]. In this contribution, a feasibility study of the lesions' detection employing the total-body J-PET scanner is presented.

**Methods**

In this study, to estimate metastasis lesions detectability by Total-Body J-PET with GATE software, a series of digital XCAT phantoms with (as experimental population) and without (as control population) centimeter grades lesions in the liver are used.

**Results**

The comparison of reconstructed images of the XCAT phantoms both from the control and experimental population is used as a metric to determine lesion detectability of Total-Body J-PET. Utilization of the advanced coincidence preselection along with the larger axial field-of-view (AFOV) coverage by Total-Body J-PET, enhance the detection of the centimeter grade lesions. It was established that the lesion detectability depends on the lesion size and the target to background ratio (TBR).

**Conclusions**

It was demonstrated that the Total-Body J-PET tomograph will be capable of detecting centimeter-grade lesions with 2:1 TBR. The achieved results confirmed the influence of large AFOV of Total-Body J-PET in the improvement of the sensitivity and subsequently detection of less aggressive lesions (1 cm with 2:1 TBR).

**Acknowledgements**

The presented study is on behalf of the J-PET collaboration, This work was supported by Foundation for Polish Science through TEAM POIR.04.04.00-00-4204/17, the National Science Centre, Poland (NCN) through grant No. 2021/42/A/ST2/00423, PRELUDIUM 19, agreement No. UMO-2020/37/N/NZ7/04106 and the Ministry of Education and Science under the grant No. SPUB/SP/530054/2022. The publication has been also supported by a grant from the SciMat and qLife Priority Research Areas under the Strategic Programme Excellence Initiative at the Jagiellonian University. The work has been also supported by the Jagiellonian University via project CRP/0641.221.2020, and via grant from the SciMat and qLife Priority Research Areas under the Strategic Programme Excellence Initiative at the Jagiellonian University.

**References**

1. Vandenberghe S, Moskal P, and Karp J. State of the art in total body PET. EJNMMI Phys. 2020. 7:1–33.

2. Moskal P, Dulski K, et al. Positronium imaging with the novel multiphoton PET scanner. Sci. Adv. 2021. 7: eabh4394.

3. Moskal P, Stępień E. Prospects and Clinical Perspectives of Total Body PET Imaging Using Plastic Scintillators. PET Clinics. 2020. 5:: 439–452.

## P31 A physiologically based pharmacokinetic (PBPK) model for dynamic ^18^F-FDG imaging on large axial field of view PET

### Mohamed Kassar^1^*, Jiaxi Hu^2^, Hasan Sari^2,4^, Milos Drobnjakovic^2^, Song Xue^2^, Francesca De Benetti^1^, Thomas Wendler^1^, Nassir Navab^1^, Sibylle Ziegler^3^, Axel Rominger^2^, Kuangyu Shi^1,2^

#### ^1^Computer Aided Medical Procedures and Augmented Reality, Institute of Informatics I16, Technical University of Munich, Munich, 85748, Germany; ^2^Department of Nuclear Medicine, Inselspital, Bern University Hospital, University of Bern, Bern, 3010, Switzerland; ^3^Department of Nuclear Medicine, University Hospital of Munich, LMU Munich, Munich, 81377, Germany; ^4^Advanced Clinical Imaging Technology, Siemens Healthcare AG, Lausanne, Switzerland

##### **Correspondence:** Mohamed Kassar (mohamed.kassar@tum.de)

*EJNMMI Physics* 2023, **10(1):**P31

**Background**

Large axial field of view (LAFOV) PET enables whole-body dynamic imaging and provides the unique opportunity to describe complete pharmacokinetic procedure. Physiologically based pharmacokinetic (PBPK) models are often applied in pharmacology to describe delivery and interaction of pharmaceuticals with multiple organs in the body. This study aims to establish an approach for PBPK modelling on dynamic LAFOV PET.

**Materials and methods**

Twenty-four oncological subjects underwent dynamic 18F-FDG scans for 65 min after intravenous bolus injection of 18F-FDG a LAFOV PET/CT system. Time activity curves (TACs) of multiple organs such as liver, spleen, heart, lungs, pancreas, intestine, kidneys, bladder, artery, and vein were extracted by semi-automatic or manual outline. A novel systematic fitting approach was developed to fit multiple interrelated ordinary differential equations (ODEs) on the measurements and estimate PBPK parameters for the investigated tracer.

**Results**

Our preliminary tests showed the systematic fitting converges. It is able to solve the model’s ODEs numerically and the fitting results generally agree with the generated TACs. The accuracy of fitting is sensitive to the initial conditions of the numerical solution of system’s ODEs. The fitting of liver, lungs and kidneys is still not perfect. The bladder has large deviation and needs further optimizations.

**Conclusions**

The preliminary results demonstrated that it is feasible to establish systemic estimation of PBPK model on the whole-body dynamic PET imaging. The optimization of the tissue curves, fitting and physiological interpretation of the new approach is ongoing.

## P32 Clinical validation of a population-based input function for 20-min dynamic whole-body ^18^FDG multiparametric PET imaging

### André H. Dias^1^*, Anne M. Smith^2^, Vijay Shah^2^, David Pigg^2^, Lars C. Gormsen^1,3^, Ole L. Munk^1,3^

#### ^1^Aarhus University Hospital, Denmark; ^2^Siemens Medical Solutions USA Inc., TN, USA; ^3^Department of Clinical Medicine, Aarhus University, Aarhus, Denmark.

##### **Correspondence:** André H. Dias (andre.dias@auh.rm.dk)

*EJNMMI Physics* 2023, **10(1):**P32

**Aim/introduction**

Multiparametric FDG PET imaging based on the Patlak model requires the late (50–70 min) dynamic whole-body (D-WB) tissue data combined with the full (0–70 min) image-derived input function (IDIF). Our aim was to replace the IDIF with a population-based input function (PBIF) and validate its use in a large cohort of patients, allowing us to develop a clinical feasible 20-min multiparametric scan protocol (Fig. 1).

**Materials and methods**

Invasive 120-min arterial input functions were sampled in 20 patients and used to generate a PBIF by using a multi-exponential model, describing tracer behaviour in the circulation, convolved by the shape of the tracer infusion (Fig. 2). The PBIF was scaled to the patient’s individual IDIF from 50–70 min.

The PBIF’s performance was tested against a cohort of 171 unique patients that had 70-min D-WB FDG scans, divided into 6 pairs of “test” vs “control” groups according to the presence/absence of: diabetes, low cardiac ejection fraction, elevated blood pressure, excess weight, low eGFR and advanced age. Finally, the PBIF was used to generate 20-min D-WB-PET parametric images, which were compared to standard 70-min D-WB images.

**Results**

When testing the 12 reference subgroups based on patient characteristics, the use of a PBIF to replace the IDIF had a bias between 1 and 5%, except for patients with diabetes or with low eGFR, where the biases were marginally higher at 7%. Multiparametric images based on a short 20-min D-WB-PET and the reference PBIF (Fig. 3) were visually indistinguishable from images produced by the full 70-min D-WB-PET and individual IDIF.

**Conclusion**

We have developed and validated a PBIF that enables multiparametric imaging based on a 20-min FDG D-WB PET acquisition on both conventional and total-body PET/CT scanners.

**Fig. 1:**
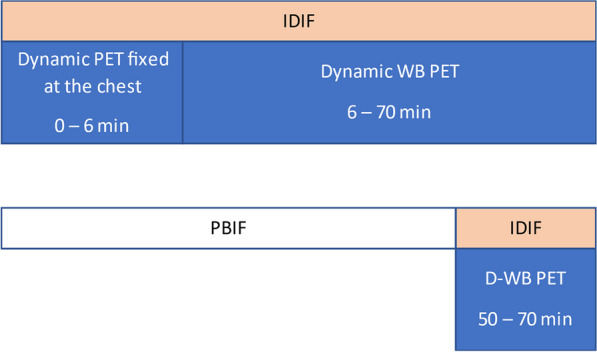
The two scan protocols on Siemens Biograph Vision 600.

**Fig. 2:**
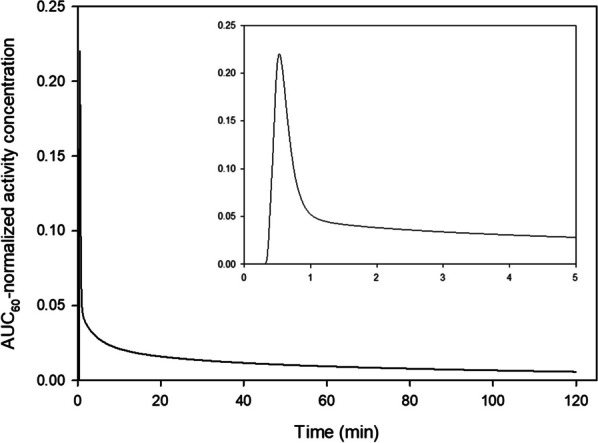
The PBIF.

**Fig. 3:**
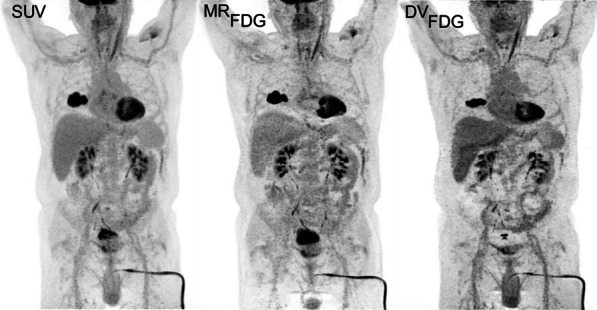
Multiparametric images of a 20-min D-WB PET/CT scan.

## P33 Total-body dual-isotope PET imaging is a useful tool for probing metabolic heterogeneity in lung cancer

### Robert Bielik, Gavin Brown, Agata Mrowinska, Gaurav Malviya, Dmitry Solovyev, David Y. Lewis*

#### Cancer Research UK Beatson Institute, Garscube Estate, Switchback Road, Glasgow, G61 1BD, Scotland, UK

##### **Correspondence:** David Y. Lewis (lewis@beatson.gla.ac.uk)

*EJNMMI Physics* 2023, **10(1):**P33

**Background**

Metabolic heterogeneity is a hallmark of lung cancer [1]. PET imaging can non-invasively detect altered metabolism of tumours but new imaging methods are required to capture the broad spectrum of metabolically diverse lung tumours [2]. Herein, we show that total-body dual-isotope PET imaging with two metabolic probes can be used for probing tumour heterogeneity in lung cancer.

**Materials and methods**

We used a Kras^G12D/+^, p53^−/−^ genetically engineered mouse model of non-small cell lung cancer and evaluated the metabolic heterogeneity in vivo by dynamic, total-body, dual-isotope PET/MRI imaging using [1-^11^C]acetate (ACE) and [2-^18^F]fluorodeoxyglucose (FDG). We developed an imaging protocol for the sequential injections of two metabolic probes and a protocol for a bolus co-injection of both tracers to follow the biodistribution which was resolved based on two-time point radioactivity measurements and the different half-life of C-11 (20.4 min) and F-18 (109.8 min).

**Results**

We found that dual-isotope PET imaging was able to detect two distinct metabolic phenotypes in the Kras^G12D/+^, p53^−/−^ model of lung cancer—one was associated with increased glycolysis (FDG-avid tumours) and the second was linked to enhanced oxidative metabolism and fatty acid metabolism (ACE-avid tumours). We characterized both tumour subtypes and defined optimal protocol for the imaging. We saw that early imaging with [1-^11^C]acetate was useful for the detection of oxidative metabolism whereas late imaging showed increased specificity for de novo fatty acid synthesis in lung tumours. An example of PET images with ACE and FDG-avid tumours is shown in Fig. 1A and time-activity curves are in Fig. 1B.

**Conclusions**

We demonstrated that dual-isotope PET/MR imaging can be a useful tool for probing metabolic heterogeneity in a model of non-small cell lung cancer. This method could be translated and applied in clinical practice to enable visualization of tumour heterogeneity, aid patient stratification and guide personalized therapy.

**Fig. 1:**
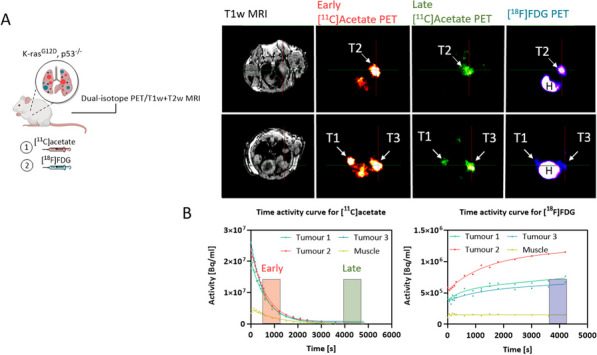
Total-body dual-isotope PET/MR imaging using [^11^C]acetate and [^18^F]FDG in the Kras^G12D/+^, p53^−/−^ mouse model of lung cancer. (A) Transversal PET/MRI images for three different tumours—PET images were acquired early between 10 and 20 min post-injection, late between 80 and 100 min post-injection for [^11^C]acetate and late for [^18^F]FDG. Tumours T1, T2 and T3 are arrowed, and H indicates the heart. (B) Time activity curves for [^11^C]acetate and [^18^F]FDG in the tumours and a muscle. Windows of optimal acquisition time are highlighted for the early and late imaging with [^11^C]acetate and [^18^F]FDG.

**References**

1. Hensley CT, Faubert B, Yuan Q, Lev-Cohain N, Jin E, Kim J, et al. Metabolic Heterogeneity in Human Lung Tumors. Cell. 2016; 164(4): 681–694.

2. Lewis DY, Boren J, Shaw GL, Bielik R, Ramos-Montoya A, Larkin TJ, et al. Late Imaging with [1-11C]Acetate Improves Detection of Tumor Fatty Acid Synthesis with PET. J Nucl Med 2014; 55(7): 1144–1149.

## P34 Signal-to-noise ratio of ^89^Zr monoclonal antibody data using a long axial field of view PET/CT for different maximum ring difference reconstructions

### Philipp Mohr*, Laura Providência, Joyce van Sluis, Adriaan A. Lammertsma, Adrienne H. Brouwers and Charalampos Tsoumpas

#### Medical Imaging Center, University Medical Center Groningen, University of Groningen, Groningen, Netherlands

##### **Correspondence:** Philipp Mohr (p.mohr@umcg.nl)

*EJNMMI Physics* 2023, **10(1):**P34

**Background**

Zr-89 immunoPET images suffer from high noise due to low count rates associated with the long half-life of Zr-89 (T_1/2_ = 78.4 h), needed to match the slow kinetics of monoclonal antibodies (mAbs). Long axial field-of-view (LAFOV) PET systems have shown an improvement in signal-to-noise ratios. Fully 3D reconstructions can maximize these benefits, but also result in a non-uniform sensitivity profile across the axial FOV [1]. The purpose of this study was to compare noise and semi-quantitative parameter values between images reconstructed with a maximum ring difference (MRD) of 322 and of 85 for different scan durations.

**Materials and methods**

A patient with metastatic breast cancer was scanned on day 4 after injection of 37 MBq Zr-89-mAb. List mode PET data was reconstructed offline (E7Tools, Siemens Healthineers) with 1.65 mm isotropic voxels for three scan durations (30, 10 and 3 min) and two MRD settings (MRD85, MRD322). A 3 cm diameter sphere was placed in the liver to assess the coefficient of variation (COV). Two lesions, corresponding to cervical lymph nodes (Fig. 1), were segmented with a semi-automatically method A50P, a contrast corrected threshold for local tumor-to-background activity at 50% of the lesion’s SUVpeak, to investigate differences in SUVmax and SUVpeak.

**Results**

SUVmax values of the lesions were higher with MRD85 than with MRD322 for all scan durations, while SUVpeak was similar (Table 1). The COV in the liver was 10.3 and 7.9% (30 min), 18.8 and 14.1% (10 min), and 33.6 and 23.4% (3 min) for MRD85 and MRD322, respectively.

**Conclusions**

Fully 3D reconstruction of LAFOV PET data reduces noise levels in the liver (centre of FOV) by 25–30%.. Using the LAFOV PET scanner, even a 3 min unfiltered image provides reasonable image quality for lesion identification in immunoPET, when using fully 3D reconstruction with MRD322 (Fig. 1).

**Table 1:** SUVmax, SUVpeak and SUVmean of two lesions using a 41% SUVmax iso-contour. L1: cervical lymph node, L2: sacrum.

Scan duration		30 min		10 min		3 min	
SUV		Max	Peak	Max	Peak	Max	Peak
L1	MRD85	15.1	6.1	15.1	6.1	15.5	6.1
	MRD322	14.0	5.9	13.6	6.0	14.7	5.8
L2	MRD85	11.7	4.1	14.0	4.3	13.6	4.5
	MRD322	10.6	4.1	12.5	4.3	14.1	4.5

**Fig. 1:**
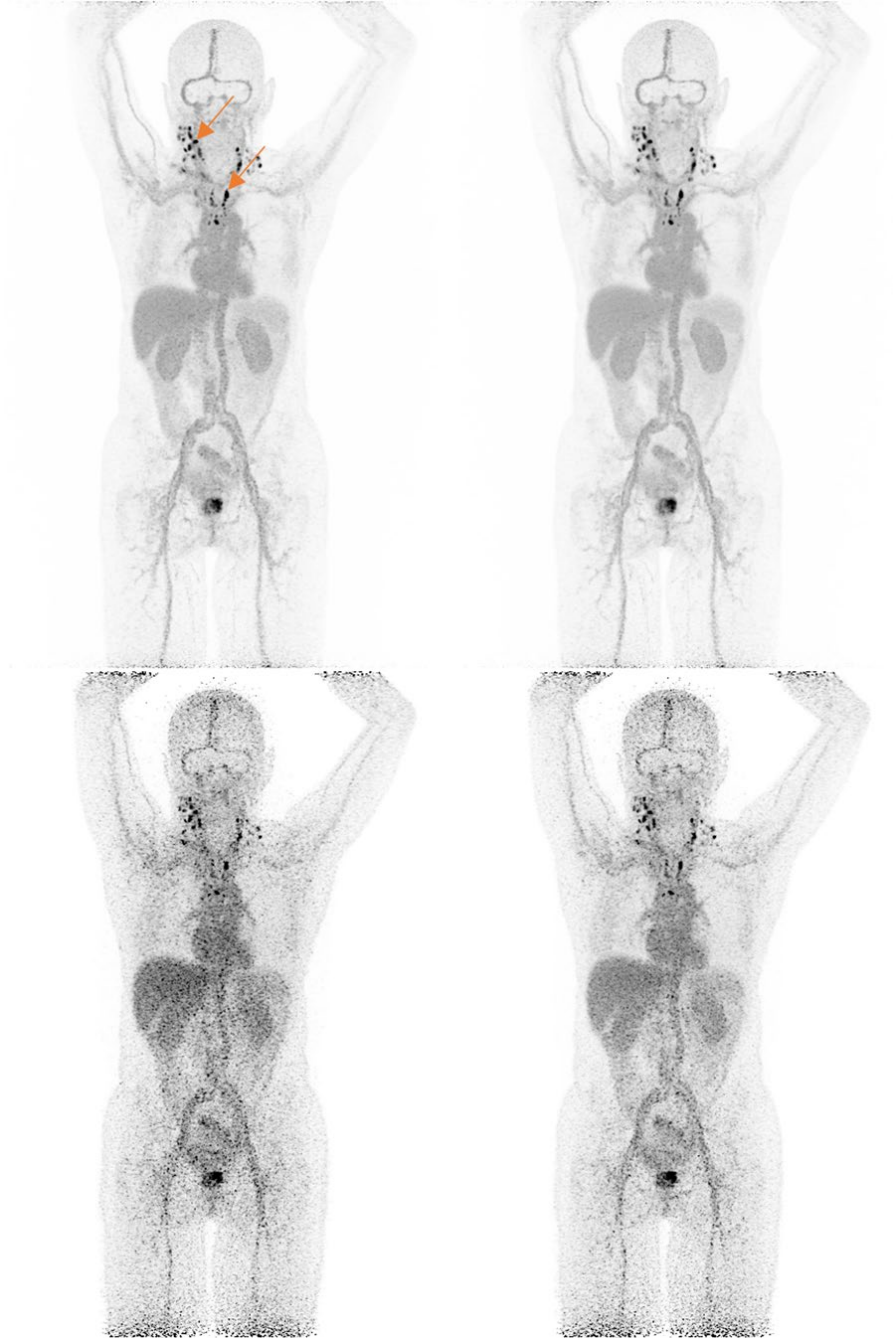
Maximum intensity projections for 30 min and 3 min scan durations (top and bottom), and MRD85 and MRD322 reconstruction settings (left and right). The two quantified lesions are indicated by arrows.

**References**

Prenosil, G.A., Sari, H., Fürstner, M. et al. Performance Characteristics of the Biograph Vision Quadra PET/CT System with a Long Axial Field of View Using the NEMA NU 2-2018 Standard. J Nucl Med 63, 476–484 (2022).

## LB1 Parameterised Geant4 simulation for total body PET research

### Benjamin M Wynne^1*^, Matthew Needham^1^, Adriana S Tavares^2,3^

#### ^1^School of Physics and Astronomy, University of Edinburgh, Edinburgh, UK; ^2^Edinburgh Imaging, University of Edinburgh, Edinburgh, UK; ^2,3^BHF-University Centre for Cardiovascular Science, University of Edinburgh, Edinburgh, UK

##### **Correspondence:** Benjamin M Wynne (b.m.wynne@ed.ac.uk)

*EJNMMI Physics* 2023, **10(1):**LB1

**Introduction**

Total-body positron emission tomography (PET) imaging has the potential to transform medical care of a number of diseases and augment our knowledge of systems biology. Various detector designs and geometries are currently under development for total-body PET imaging of humans. This variety of PET scanner detector geometries coming to market at present, and in particular the variation in axial field-of-view (aFOV), motivates a need to compare the performance of these devices in a consistent simulated environment.

**Methods**

We present an open-source Geant4 simulation package [https://github.com/bwynneHEP/SimplePetScanner], allowing variation of relevant parameters such as detector aFOV and the radioactive tracer isotope from the Linux command line. Two simplified detector geometries based on the EXPLORER [1] and Siemens Quadra [2] models are supported with variable granularity. Intrinsic radioactivity of the detector crystals is fully simulated. The simulation can be viewed with the built-in GUI, and results are saved in a plain text format for easy analysis.

**Results**

Example Python analysis code is provided with the simulation, demonstrating calculation of the noise equivalent count rate (NECR) figure of merit using an approximation to the NEMA NU 2–2012[3] standard method (Fig. 1). Preliminary results show a dependence between the detector aFOV and the length of the source, with peak NECR plateauing as the detector extends beyond the region of interest (Fig. 2).

Additional outputs from the simulation are demonstrated, such as phantom attenuation measured using the detector intrinsic radioactivity (Fig. 3).

**Conclusion**

Simulation allows for rapid assessment of detector performance in a variety of scenarios, demonstrating a link between aFOV and test source length that was not clear from published experimental data. Further studies with low source activity, or detector intrinsic radioactivity, may also demonstrate the importance of detector aFOV.

**Fig. 1:**
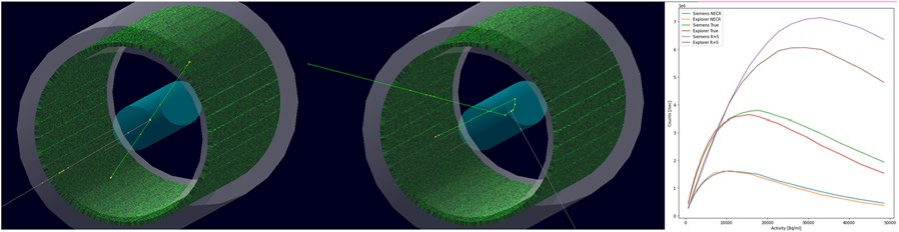
Example simulations of “true” (i.e. unscattered) and scattered positron decay events from an F18 linear source are shown in a detector based on the Siemens Quadra model. NECR is calculated with decaying source activity, showing very similar performance for the Siemens Quadra and EXPLORER detectors with a 700 mm linear phantom.

**Fig. 2:**
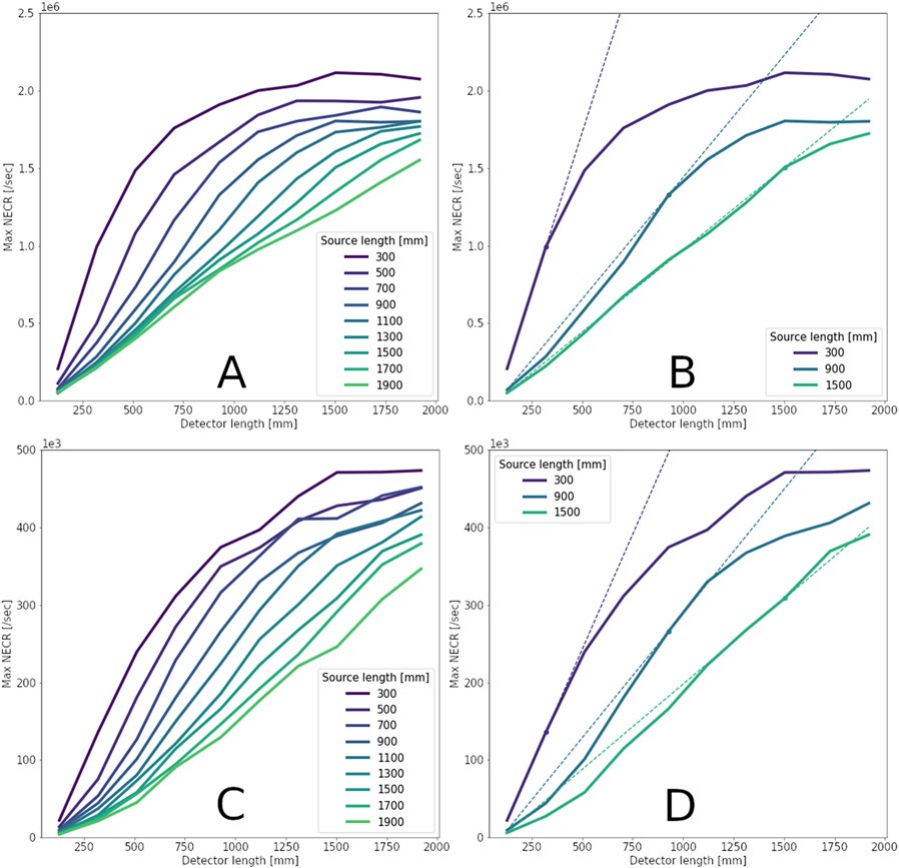
Peak NECR as the source activity decays is investigated for varying detector aFOV and length of the linear source. Results for an initial F18 source activity of 1100 MBq are shown in subfigures **A** and **B**, and for 20 MBq initial activity in subfigures **C** and **D**. In each case we see an approximately linear improvement in peak NECR with detector aFOV until the point where detector aFOV equals source length, when the improvement begins to plateau.

**Fig. 3:**
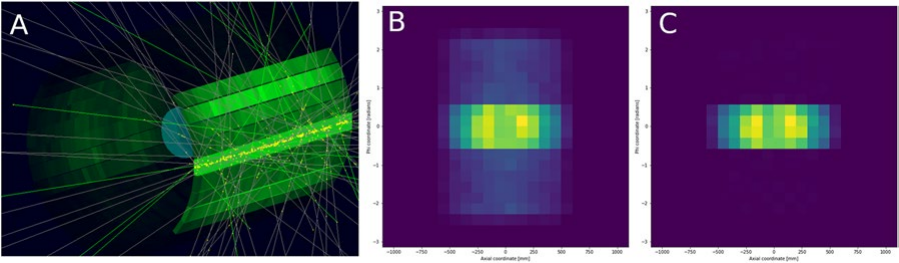
Example measurement of photon attenuation in a cylindrical phantom, using the intrinsic radioactivity of Lutetium in the detector crystals. Total deposited energy is presented within the rendered simulation (**A**) and as flattened histograms with photon energy windows of 100–900 keV (**B**) and 300–600 keV (**C**). These results use 2 × 10^7^ simulated events, corresponding to ~ 1.3 s activity. Attenuation due to the 100 mm phantom is visible in the central region.

**Acknowledgements**

This research is supported by a grant from the STFC cancer diagnosis network.

**References**

[1] Spencer BA, Berg E, Schmall JP, Omidvari N, Leung EK, Abdelhafez YG, Tang S, Deng Z, Dong Y, Lv Y, Bao J. Performance evaluation of the uEXPLORER total-body PET/CT scanner based on NEMA NU 2-2018 with additional tests to characterize PET scanners with a long axial field of view. Journal of Nuclear Medicine. 2021 Jun 1;62(6):861–70.

[2] Prenosil GA, Sari H, Fürstner M, Afshar-Oromieh A, Shi K, Rominger A, Hentschel M. Performance Characteristics of the Biograph Vision Quadra PET/CT System with a Long Axial Field of View Using the NEMA NU 2-2018 Standard. Journal of nuclear medicine. 2022 Mar 1;63(3):476–84.

[3] Nema NU. NU 2-2012: Performance Measurements of Positron Emission Tomographs. Rosslyn, VA: National Electrical Manufacturers Association. 2012.

## LB2 The effect of different surface finishes on Cerenkov and scintillation photon collection efficiency in pixelated and monolithic BGO and L(Y)SO detectors

### Jens Maebe*, Stefaan Vandenberghe

#### Department of Electronics and Information Systems, Ghent University, Ghent, Belgium

##### **Correspondence:** Jens Maebe (Jens.Maebe@UGent.be)

*EJNMMI Physics* 2023, **10(1):**LB2

**Background**

The surface finish of PET scintillation crystals can have a large effect on the optical photon transport and collection efficiency, thereby affecting the timing, the energy resolution and for monolithic detectors, positioning accuracy of gamma interactions. Cerenkov enables TOF in BGO, but timing is based on a small number of emitted photons and therefore it becomes critical to optimize the surfaces.

**Materials and methods**

GATE v9.2 was used to simulate 511 keV gamma photon interactions in different PET detectors. It further tracks the optical (scintillation and Cerenkov) photon transport to the detector face, with surface reflections modelled by the LUT Davis Model [1]. We investigate different detector geometries (monolithic 50 × 50 × 16 mm^3^ and pixelated 3 × 3 × 20 mm^3^), materials (LYSO and BGO), side surfaces and detector surfaces and compare their collection efficiencies.

**Results**

Figure 1 shows a comparison of the optical photon collection efficiency for the different detectors and surfaces. Black side surfaces lead to the lowest collection efficiency while reflective side surfaces show the highest. A rough surface at the readout side leads to a larger number of detected photons. It transmits fewer of the low angle-of-incidence photons but allows for more of the high angle-of-incidence photons to pass. This results in improved collection efficiency for monolithic detectors or reflective side surfaces because in this case a relatively large amount of photons reach the detector with a high incidence angle. For monolithic BGO, Fig. 2 shows that a rough detector surface also allows the very first photons to be detected more efficiently. The effect is not as pronounced for LYSO or a pixelated detector.

**Conclusion**

The use of a rough detector surface improves the timing resolution for monolithic BGO detectors, especially for reflective surfaces. The trade-of is of course more difficult positioning, but this could potentially be resolved with deep learning.

**Fig. 1:**
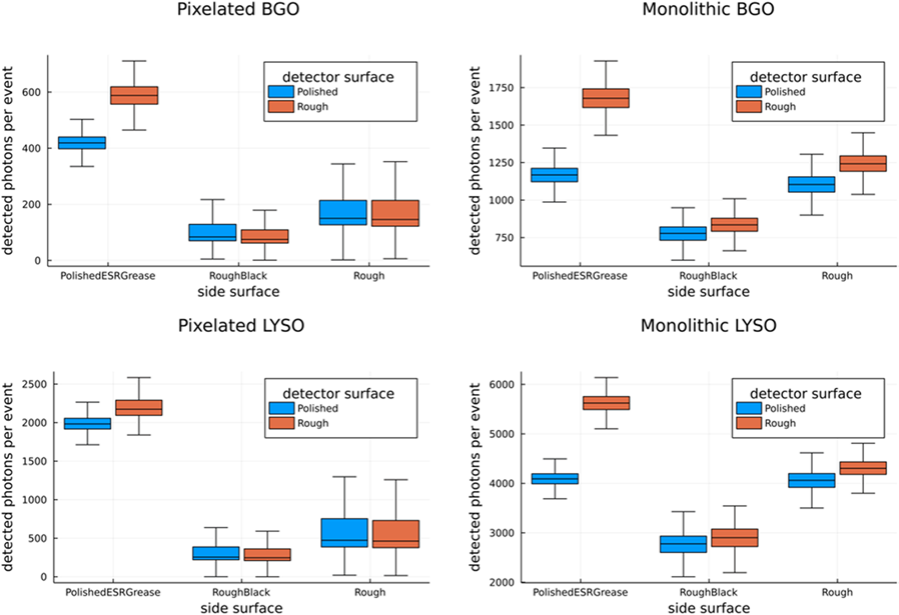
Total number of photons detected per gamma event for different detectors and surface finishes, assuming an SiPM photon detection efficiency of 50%. Both detector surfaces are coupled to the SiPM array by optical grease (refractive index = 1.5). The side surfaces are a polished surface coupled with optical grease to a specular reflector (PolishedESRGrease), a rough surface coated with black paint (RoughBlack) and a rough surface with no coating (Rough). The top surface is always PolishedESRGrease.

**Fig. 2:**
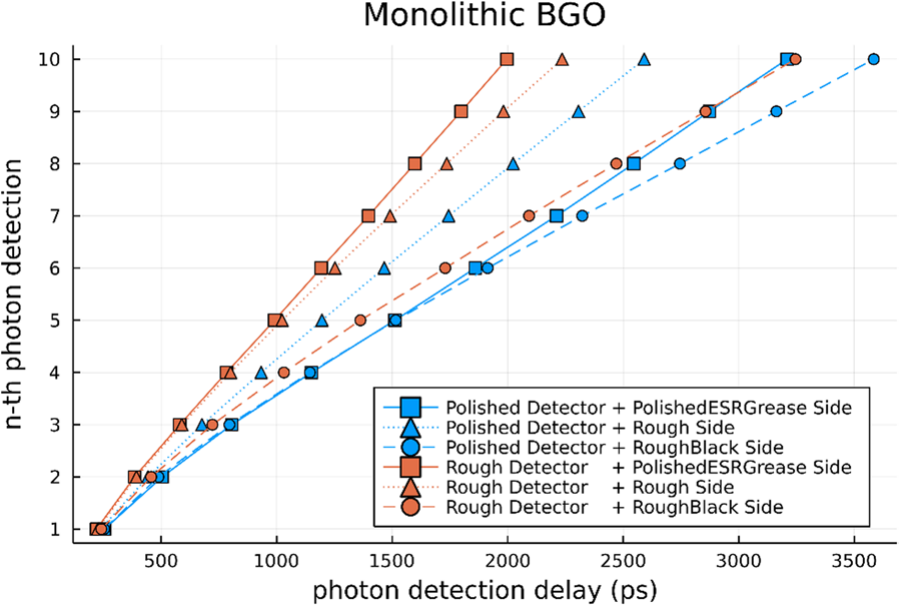
Average delay between the gamma photon entering the scintillation crystal and the detection of the n-th optical photon for monolithic BGO, with an SiPM photon detection efficiency of 50%.

**References**

[1] Roncali and Cherry. Simulation of light transport in scintillators based on 3D characterization of crystal surfaces. Phys. Med. Biol. 2013; 58:2185–2198.

## LB3 3DΠ, a novel design of TB-TOF-PET scanner, using Xenon-doped Liquid Argon detector with SiPM

### Azam Zabihi^1^*, Masayuki Wada^1^, Xinran Li^2^, Andrew Renshaw^3^, Alejandro Ramirez^3^, Michela Lai^4^, Federico Gabriele^5^, Cristian Galbiati^6,7^, Davide Franco^8^

#### ^1^Nicolaus Copernicus Astronomical Center of the Polish Academy of Sciences, ASTROCENT, Warsaw, Poland; ^2^Lawrence Berkeley National Laboratory, Berkeley, United States of America; ^3^Department of Physics, University of Houston, Houston, United States of America; ^4^Department of Physics, University of Cagliari, Cagliari, Italy; ^5^INFN Cagliari Division, INFN, Cagliari, Italy; ^6^Gran Sasso Science Institute,L’Aquila 67100, Italy; ^7^Department of Physics, Princeton University Princeton, United States of America; ^8^APC, University of Paris, CNRS, Astroparticle and Cosmology, Paris, France

##### **Correspondence:** Azam Zabihi (azabihi@camk.edu.pl)

*EJNMMI Physics* 2023, **10(1):**LB3

**Background**

This project is a medical imaging application of the ongoing R&D from the DarkSide collaboration, a direct dark matter particle search via Liquid Argon (LAr) targets, where LAr time projection chambers are used to identify particle interactions for WIMP detection. The collaboration has demonstrated the true power of the advancing LAr detector technology. They are also making significant strides in low-radioactivity argon procurement and cryogenic photosensor development and fabrication. With these advances in hand, the principle of 3DΠ has been developed.

**Materials and methods**

The 3DΠ is a Total-Body (TB), Time Of Flight (TOF), Positron Emission Tomography (PET) silicon photomultiplier–based scanner. It utilizes a Xenon-doped LAr (LAr + Xe) scintillator, with an axial field-of-view (AFOV) of 200 cm and 9 double-sided concentric rings of SiPM panels. The 3DΠ Monte Carlo simulation package has been derived from the DarkSide simulation package based on the Geant4 toolkit. The analysis was conducted via ROOT. Our purpose was to evaluate the performance of the 3DΠ scanner using the NEMA NU 2–2018 standards for spatial resolution, sensitivity, image quality, count rate performance, and timing resolution.

**Results**

The spatial resolution at full width at half maximum at 1 cm and center AFOV, in the radial, tangential, and axial directions are 9.18, 9.53, and 7.66 mm, respectively. The sensitivity is 510.39 kcps/MBq at the center and 439.95 kcps/MBq at a 10 cm offset. The peak noise-equivalent count rate (NECRs) is 6.93 × 10^4^ kcps, for an activity of 176.02 MBq and the respective scatter fraction is 35%.

**Conclusions**

These preliminary results demonstrate that our scanner's system performance is comparable to, if not better than other, commercial scanners.

## LB4 Rigid body motion analysis in walk-through total body PET scanner based on real-time motion tracking with cameras: comparative study between free-breathing and breath-hold

### Florence Marie Muller^1^, Jens Maebe^1^, Meysam Dadgar^2^, Nadia Withofs^3^, Christian Vanhove^1^, Stefaan Vandenberghe^1^

#### ^1^Medical Image and Signal Processing (MEDISIP), Ghent University, Ghent, Belgium; ^2^Faculty of Physics, Astronomy and Applied Computer Science, Jagiellonian University, Krakow, Poland; ^3^Division of Nuclear Medicine and Oncological Imaging, Department of Medical Physics, CHU de Liège, Liège, Belgium

##### **Correspondence:** Florence Marie Muller (florencemarie.muller@ugent.be)

*EJNMMI Physics* 2023, **10(1):**LB4

**Background**

Total Body (TB) PET systems have become so sensitive that 30-s body acquisitions seem feasible. Practical patient throughput is however limited mostly by patient positioning on the bed. A new flat panel high-resolution Walk Through (WT) TB-PET design with patients in upright position was therefore proposed [1]. To investigate the extent of patient motion (which can be larger in such a design) a WT-TB-PET mock-up was built (Fig. 1). Motion analyses compared the impact of free-breathing (normal) vs breath-hold induced body motion in the WT-TB-PET.

**Materials and methods**

The study included 15 ‘healthy’ participants. The subject wore a body-tight surf shirt (to accurately detect breathing motion at chest and abdomen), a cutting collar around the neck with two markers at the shoulders and glasses with patterns (for head motion). To estimate chest and abdominal movement information (with/without breath-hold), surface motion of a checkerboard sticker placed on the body was tracked and analysed. A 30-s ‘acquisition’ with real-time motion tracking was performed using four webcams (Fig. 1).

**Results**

Figure 2 shows that shoulder motion is negligible, while head motion is most significant and will need to be compensated for when including the brain in the field-of-view. It was observed that, on average, breath-hold reduces rigid body motions (as further exemplified by the positioning curves shown in Fig. 3). Three participants had larger average motions during breath-hold than with free-breathing, suggesting difficulties in holding breath for 30 s or related to variations in breath-hold techniques.

**Conclusions**

Motion of subjects standing upright in the WT-TB-PET is limited (and largest for the head) and close to the expected spatial resolution of 2 mm. The comparative study demonstrates that a 30 s breath-hold is feasible and minimizes body motion. Further testing with PET-specific patient groups will be performed to assess breath-hold techniques and potential motion compensation techniques.

**Fig. 1:**
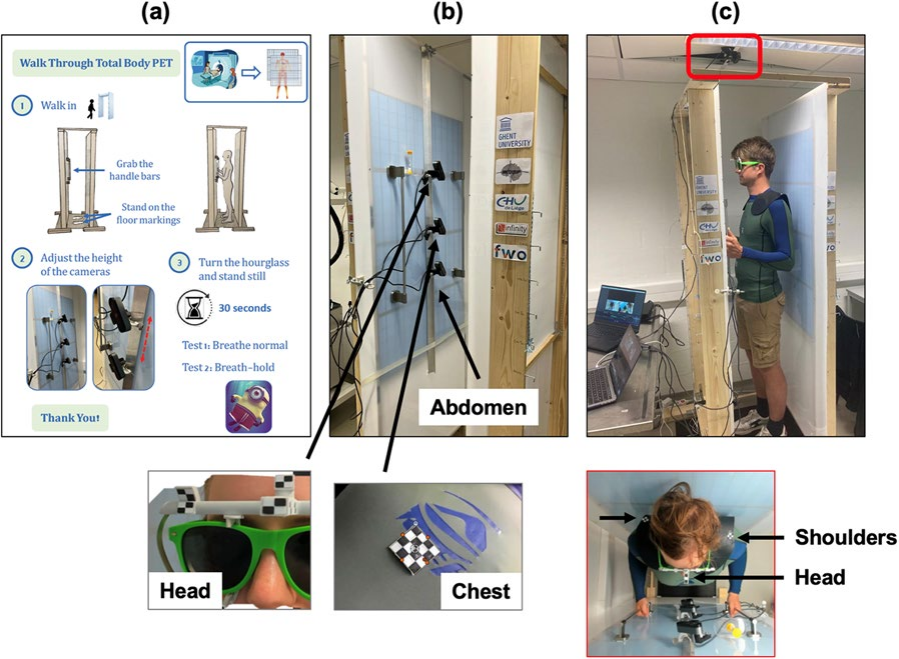
(**a**) Pictogram instruction sheet given to the participants. (**b**) Three web cameras facing the front of the person and centered to the height of the glasses (for head motion), chest and abdomen (to detect breathing motion through surface movement tracking of a checkerboard pattern). (**c**) One web camera placed at the top to track shoulder and head motion.

**Fig. 2:**
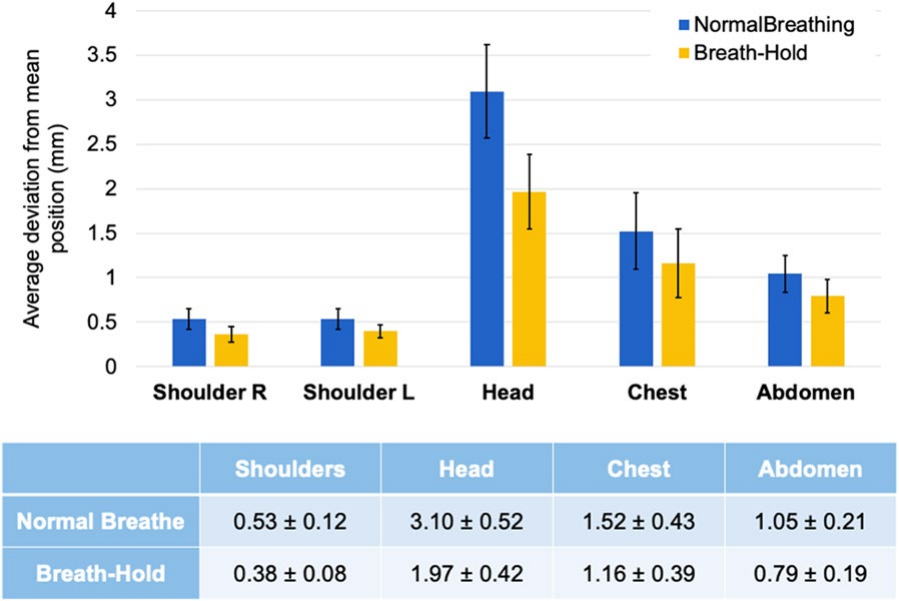
Motion analysis results shown as bar graphs. The deviation (distance) from the mean position (± standard deviation) averaged across all 15 participants is reported. Results are compared between free-breathing (normal) and breath-hold.

**Fig. 3:**
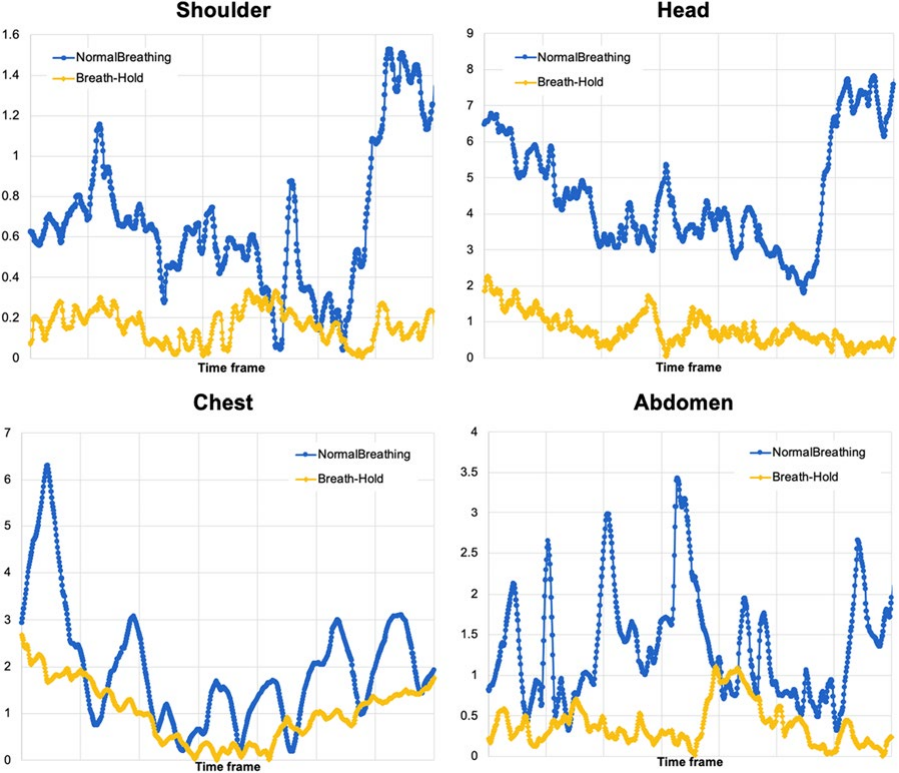
Using the Apple Motion software, tracker points were placed at the respective positions of interest and here examples of recorded tracking (positioning) curves are shown. Deviation from mean position is plotted over the whole ‘acquisition’ time of 30 s. Results are compared between free-breathing (normal) and breath-hold.

**Reference**

[1] Vandenberghe S. High throughput cost-efficient Flat panel monolithic Walk Through PET (https://indico.koza.if.uj.edu.pl/event/7/contributions/682/). 4th Jagiellonian Symposium on Advances in Particle Physics and Medicine. Krakow, Poland; July 2022.

## LB5 Kinetic modelling of dynamic ^18^F-FDG datasets from a long axial field-of-view PET scanner using model selection criteria with deep learning-based organ segmentations

### Hasan Sari^1,2^*, Vijay Shah^3^, Clemens Mingels^2^, Ian Alberts^2^, Maurizio Conti^3^, Lars Eriksson^3^, Kuangyu Shi^2^, Paul Cumming^2,4^, Axel Rominger^2^

#### ^1^Advanced Clinical Imaging Technology, Siemens Healthcare AG, Lausanne, Switzerland; ^2^Department of Nuclear Medicine, Inselspital, Bern University Hospital, Bern, Switzerland; ^3^Siemens Medical Solutions, USA Inc., Knoxville, Tennessee, USA; ^4^School of Psychology and Counselling, Queensland University of Technology, Brisbane, Australia

##### **Correspondence:** Hasan Sari (hasan.sari@siemens-healthineers.com)

*EJNMMI Physics* 2023, **10(1):**LB5

**Background**

PET protocols involving dynamic acquisition and reconstruction of PET emission data allow for full quantification of tracer kinetics. With the introduction of long axial field-of-view (LAFOV) PET systems, tracer kinetics of multiple organs of interests can be studied in a single bed position without any need for scanner bed movement [1,2]. These advances enable use of non-linear compartmental kinetic models for estimation of kinetic microparameters from different structures of interest. However, the irreversible 2-tissue-compartment (2TC-3k) might cause artefacts in parametric images of some organs when applied to whole-body ^18^F-FDG datasets [3]. Here, we propose a method that utilizes deep learning-based organ segmentations to select an appropriate kinetic model for each organ of interest.

**Materials and methods**

Twenty-six oncological patients with a heterogenous set of tumour types received dynamic ^18^F-FDG scans lasting 65 min using Biograph Vision Quadra LAFOV PET/CT scanner. A deep learning-based software prototype for organ segmentation was used to obtain automatic segmentations of liver, lungs, kidneys, whole heart, aorta, spleen, bone, and whole brain. A qualified nuclear medicine physician segmented tumour lesions from 48 lesions. TACs were extracted from these regions and were fitted using three different models: blood volume model (BV), 1-tissue-compartment model (1TC-2k), and 2-tissue-compartment model (2TC-3k). Akaike information criterion (AIC) and corrected AIC (AICc) were used for evaluation of models.

**Results**

According to AIC criteria, vascular structures favoured BV model, liver, lungs, and whole heart favoured the 1TC-2k model, and kidneys, spleen, bone and whole brain favoured the 2TC-3k model (Table 1). The 2TC-3k was favoured by 92% of tumour lesions. There was a strong agreement between AIC and AICc findings. Comparison of parametric images generated using the proposed method and the standard 2TC-3k model are shown in Fig. 1.

**Conclusion**

Tissue-specific kinetic models are required for appropriate compartmental modelling of whole-body dynamic PET data from LAFOV PET scanners. The proposed method can serve to improve the accuracy of microparameter estimation and enhance whole body parametric imaging.

**Table 1:** Model preference for different organs and tumour lesions according to AIC.

	Liver (%)	Lungs (%)	Kidneys (%)	Heart (%)	Spleen (%)	Blood (%)	Bone (%)	Whole brain (%)	Tumour lesions (%)
BV	0	4	0	50	0	100	0	0	2
1TC-2 k	88	96	0	50	35	0	0	0	6
2TC-3 k	12	0	100	0	65	0	100	100	92

**Fig. 1:**
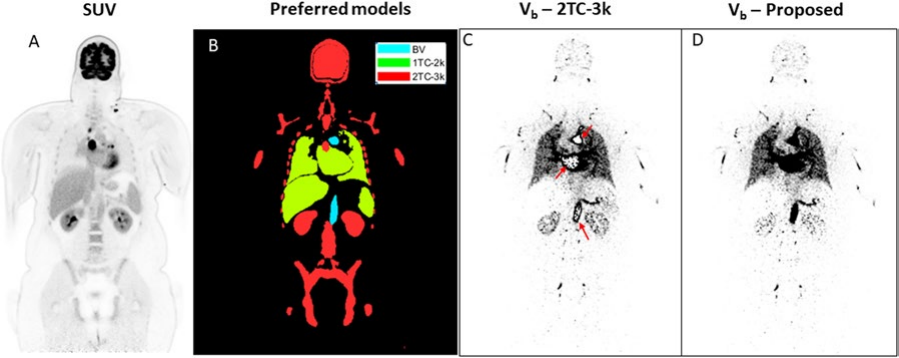
Coronal slices of SUV image (**A**), and label image showing the preferred kinetic models by tissue segment (blue: BV, green: 1TC-2 k, red: 2TC-3 k) (**B**). Parametric V_b_ (blood volume) images generated with 2TC-3 k applied to each voxel (**C**) and with the proposed method (**D**). Red arrows indicate artefacts in heart and vascular structures when the 2TC-3 k model was used to fit all voxels.

**References**

1. Wang G, Parikh M, Nardo L, et al. Total-body dynamic PET of metastatic cancer: First patient results. *J Nucl Med*. 2020;61:1–6.

2. Sari H, Mingels C, Alberts I, et al. First results on kinetic modelling and parametric imaging of dynamic 18F-FDG datasets from a long axial FOV PET scanner in oncological patients. *Eur J Nucl Med Mol Imaging*. 2022;49(6):1997–2009¨

3. Wang G, Nardo L, Parikh M, et al. Total-Body PET Multiparametric Imaging of Cancer Using a Voxel-wise Strategy of Compartmental Modeling. *J Nucl Med*. 2021;63(8)

## LB6 Total plant and veterinary animal physiology imaging simulation in a Monolithic High resolution Walk-through Flat Panel PET

### Stefaan Vandenberghe^1^*, Meysam Dadgar^2^, Maya Abi Akl^1,3^, Jens Mincke^1,4^, Kathelijne Peremans^5^, Emmelie Stock^5^, Christian Vanhove^1^, Kathy Steppe^4^

#### ^1^Medical Image and Signal Processing, Ghent University, Ghent, Belgium; ^2^Faculty of Physics, Astronomy and Applied Computer Science, Jagiellonian University, 30-348 Cracow, Poland; ^3^Science Program, Texas A&M University at Qatar; ^4^Faculty of Bioscience Engineering, Ghent University, Ghent, Belgium; ^5^Faculty of Veterinary science, Ghent University, Ghent, Belgium

##### **Correspondence:** Stefaan Vandenberghe (stefaan.vandenberghe@ugent.be)

*EJNMMI Physics* 2023, **10(1):**LB6

**Background**

Monolithic instead of pixelated detectors have the advantage of much better spatial resolution (1–1.5 mm), 6-layer DOI [1,2]. By using these detectors in a vertical flat panel TB-PET [3] both sensitivity and spatial resolution (reduced acolinearity) are optimized. This design will be used to extend our research on effects of drought and climate change (now performed on small parts of plants in Molecubes Beta-Cube PET) and cats and dogs (aggression/brain receptors and oncology) towards total subject imaging at higher resolution and sensitivity. In this work we simulate the potential such a configuration for imaging plants and veterinary animals.

**Methods**

The spatial resolution was evaluated using back-to-back photon Gate simulation of 8 point sources (Fig. 1.) A high-resolution CT scan of a large tomato plant (about 1 m high) was obtained on a clinical Siemens mCT (70 keV, sequential CT with 0.6 mm slices). These data were converted into a virtual PET with different levels of activity (lowest for leaves, middle for stem and highest for tomatoes itself) and used as input for Gate. The plant is placed upright. Spatial resolution, sensitivity and scatter fraction are compared to another Total Body PET system, Siemens Quadra [4]. The same process is repeated for a previously acquired high resolution CT scan (acquired on the same Siemens mCT) of a cat and beagle dog.

**Results**

Spatial resolution ranged from 0.9 to 1.35 mm in all positions and directions, which should result in sub 2 mm resolution if positron range and acolinearity are taken into account. As the objects are small (cats and dogs) and have low density (plants) attenuation and scatter is also significantly lower than in humans, which also enables to use the full acceptance angle (and sensitivity increase) of TB-PET as there is low negative impact of attenuation and scatter on oblique LORs.

**Fig. 1:**
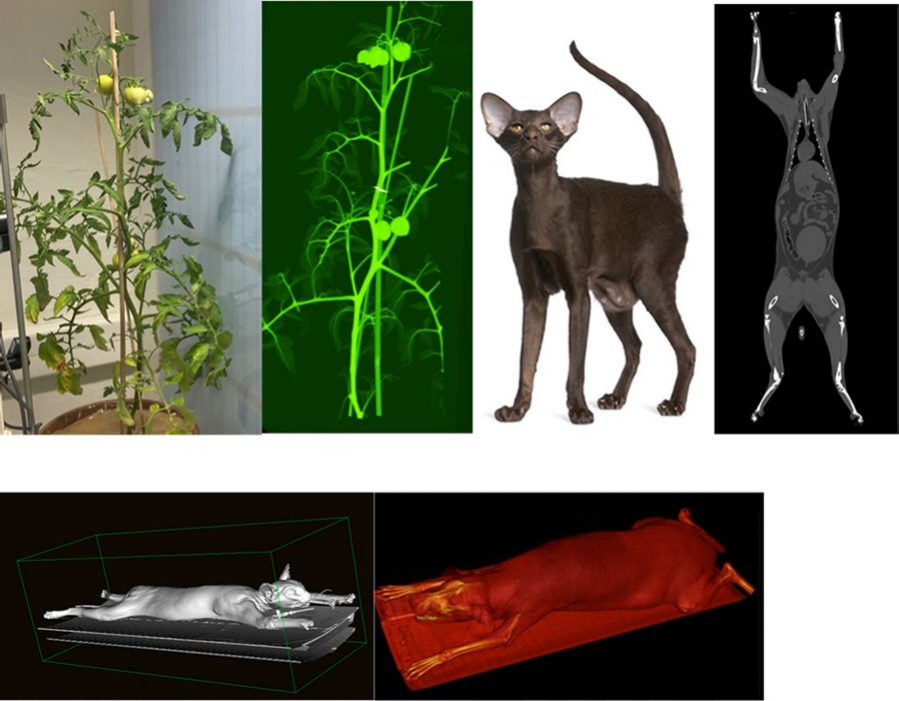
MIP rendering of acquired Full tomato plant CT (0.6 mm/70 keV) and cat-CT scan (slice and rendering) and dog render.

**Fig. 2:**
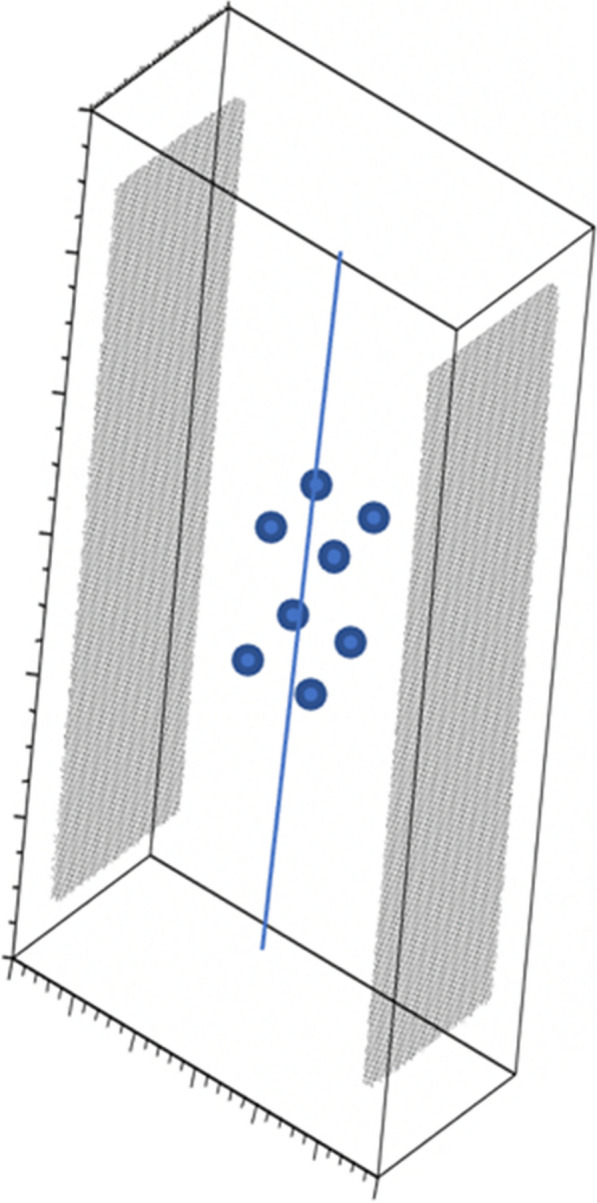
Positioning of 8 point sources in gantry of walk through PET.

**References**

1. Mariele Stockhoff, Milan Decuyper, Roel Van Holen, Stefaan Vandenberghe. High-resolution monolithic LYSO detector with 6-layer depth-of-interaction for clinical PET. Phys Med Biol. 2021; 66(15):10.1088/1361-6560.

2. P. Carra, M.G. Bisogni, E. Ciarrocchi, M. Morrocchi, V. Rosso, G. Sportelli, N. Belcari. Performance of monolithic BGO-based detector implementing a Neural-Network event decoding algorithm for TB-PET applications, presentation at Elba PSMR-TB-PET conference 2022¶

3. Vandenberghe S, Moskal P, Karp JS. State of the art in total body PET. EJNMMI Phys. 2020; 7(1):35.

4. Prenosil GA, Sari H, Fürstner M, Afshar-Oromieh A, Shi K, Rominger A, Hentschel M. Performance Characteristics of the Biograph Vision Quadra PET/CT system with long axial field of view using the NEMA NU 2-2018 Standard. J Nucl Med. 2022; 63(3):476484.

